# Insights into enhanced, divergent, and additive responses to single and combined hypoxia-salt stress

**DOI:** 10.1186/s12870-026-08595-7

**Published:** 2026-03-27

**Authors:** Angelina Jordine, Julia Alt, Christina Bonn, Pia Nolte, Joost T. van Dongen, Lisa Fürtauer

**Affiliations:** 1 Plant Molecular Systems Biology, Institute of Biology III, RWTH Aachen University, Worringer Weg 1, 52074 Aachen, Germany; 2 Molecular Ecology of the Rhizosphere, Institute of Biology I, RWTH Aachen University, Worringer Weg 1, 52074 Aachen, Germany; 3 Center for Computational Life Sciences, RWTH Aachen University, Aachen, Germany

**Keywords:** *Salicornia europaea*, Combined tolerance, Hypoxia, Salt, Halophyte, Stress response, RNA sequencing

## Abstract

Plants frequently encounter simultaneous stressors, requiring complex adaptive responses across regulatory levels. Although morphological and metabolic effects of combined hypoxia-salt stress are known in halophytes, the transcriptional mechanisms underlying these adjustments remain largely unexplored. In this study, we aimed to determine how the naturally adapted halophyte *Salicornia europaea* integrates hypoxia- and salt-specific responses at the transcriptional level to draw physiological conclusions about simultaneous stress interactions. To address this, we performed RNA sequencing of shoots and roots exposed to single and combined stress conditions. Our analysis revealed that 16% of genes were exclusively regulated under combined hypoxia-salt conditions, indicating distinct transcriptional reprogramming. Enhanced, divergent, and additive effects occurred across functional pathway categories, with divergent regulation predominating in both tissues. Pathway-level analysis uncovered unexpected strategies: up-regulation of sucrose biosynthesis genes, *SUCROSE SYNTHASE* (*SUS*), and *TREHALOSE-6-PHOSPHATE PHOSPHATASE* (*TPP*) suggested enhanced sucrose and trehalose metabolism, while down-regulation of invertases indicated strategic carbon flux redistribution. In addition, enhanced proline biosynthesis under combined stress indicated a shift towards improved osmoprotection. By combining transcriptomics with a physiological framework, we show how *Salicornia* employs non-additive and sometimes counterintuitive transcriptional strategies to coordinate carbon allocation, respiration, and osmotic balance. These findings highlight unique transcriptional adjustments that contribute to resilience under simultaneous stress.

**Supplementary Information:**

The online version contains supplementary material available at 10.1186/s12870-026-08595-7.

## Introduction

Plants established themselves in all terrestrial ecosystems by adapting to particular environmental conditions and developing tolerance mechanisms [1]. These mechanisms allow plants to survive an thrive in extreme habitats, like hot and cold deserts, alpine regions and coastal areas [2–4]. Consequently, in natural habitats, plants are often exposed to multiple abiotic stress factors simultaneously rather than being exposed to a single one. Survival in such conditions necessitates complex adaptation and tolerance mechanisms that include genome adaptations and integrate signaling transduction networks, transcriptional as well as metabolic adaptations upon abiotic stress [5–8]. Consequently, research on simultaneous stress factors in various combinations has increased rapidly in recent years [6, 8–16].

Examinations of combinations of drought, heat, high light and salinity stress simultaneously suggested that the adaptive responses to these combined factors cannot be directly predicted from how plants respond to each individual condition [9, 17, 18]. A “dominant stressor” may dictate the combined response based on severity [19]. Simultaneous stresses lead to complex interactions within their metabolic responses and reprogramming processes, involving intricate regulatory strategies such as signaling events that lead to transcriptional regulation and modified enzyme activities. Being exposed to multiple stressors, can lead to an overall additive, enhanced or divergent effects for the plant. Enhancement occurs when combined stressors have a greater effect than the sum of their individual impacts. In contrast, divergent interactions reduce the overall effect compared to the sum of individual stressors, while additive effects result in an impact equal to their sum [14, 20].

Enhanced, divergent, and additive stress response concepts have mainly been used to describe the overall effect of simultaneous stress factors, but are seldom applied to metabolic or gene expression changes [14, 20]. Hypoxia and salinity stress often co-occur in coastal regions, where plants are naturally exposed to fluctuating environmental conditions. In these dynamic habitats, especially in salt marshes, tides and freshwater input *via* rainfall create frequent changes in oxygen availability and salinity levels [21]. While the combination of hypoxia and salt stress has been studied at the morphological and metabolite levels in tolerant plant species such as *Salicornia europaea* [16], *Suaeda maritima* [5], *Phragmatis australis* [22] and *Beta vulgaris* [23], this study focuses explicitly on transcriptional reprogramming under single and combined hypoxia-salt conditions in *Salicornia europaea*.

Hypoxia and salinity independently trigger distinct metabolic responses in plants, affecting fundamental processes such as i) carbohydrate metabolism, ii) amino acid (AA) metabolism and iii) respiration and energy production. For example, cellular respiration is generally reduced under low oxygen conditions [24]. During hypoxia, transcriptional reprogramming occurs in parts of the carbohydrate metabolism (trehalose metabolism) that controls the sugar influx into glycolysis [25, 26]. Glycolysis itself and fermentation are also reprogrammed resulting in anaerobic metabolism [26, 27]. Additionally also amino acid metabolism, ethylene signaling and nitrogen usage are significantly altered under hypoxia [25, 26]. All of these adaptive mechanisms are focused on energy conservation to prevent the plant from energy shortage [28, 29].

Salt stress on the other hand, leads to ionic imbalance and osmotic stress, disturbances in cellular homeostasis, variations in respiration rates and energy production, generation of reactive oxygen species (ROS) and reduced water availability [30–32]. For example, some respiratory pathways like the tricarboxylic acid (TCA) cycle or mitochondrial electron transport chain (mETC) use amino acid degradation products as alternative substrates among others. Amino acid degradation products serve as alternative substrates that feed into carbohydrate metabolism, supporting energy homeostasis and recovery under salt stress [33, 34].

Under hypoxic conditions, energy production *via* aerobic respiration is reduced, causing a metabolic shift toward anaerobic fermentation. This change leads to enhanced sugar consumption, mobilization, and transport to maintain energy supply. However, sucrose synthesis is down-regulated, resulting in no net accumulation of sugars [28, 35]. In contrast, under salt stress, adenosine triphosphate (ATP) production remains largely unaffected, but energy demand increases due to the activation of stress-related processes. Despite a general inhibition of sugar consumption, mobilization, and transport, sucrose synthesis itself is enhanced. This leads to the accumulation of soluble sugars, acting mainly as osmoprotectants against osmotic imbalance from salinity [36].

Moreover, contrasting responses for individual hypoxia and salt stress are reported for amino acid metabolism. Glutamate (Glu) is degraded *via* GLUTAMATE DEHYDROGENASE (GDH) to recycle ammonium and conserve energy during hypoxia. Additionally, the conversion of pyruvate to alanine (Ala) is a key mechanism for energy-efficient nitrogen recycling [37]. In contrast, salt stress suppresses alanine metabolism, as indicated by reduced levels of Ala, asparagine (Asn), and glutamine (Gln) [36]. Furthermore, GLUTAMINE SYNTHETASE activity is up-regulated in roots under salt stress to support ammonium assimilation [38]. Proline strongly accumulates as an osmoprotectant, and this response is supported by the salt-induced activation of amino acid transporters (e.g. AMINO ACID PERMEASE 1, AAP1), which facilitate proline uptake from external sources [39, 40]. In contrast, under hypoxic conditions, proline does not accumulate, and no specific transporters for its uptake are induced. Under oxygen deficiency, respiration in the root tissue of *Pisum sativum* (pea) decreases, while salt stress generally either increases respiration or has no effect, depending on the species [24, 41].

Combined hypoxia-salt stress lead to increased proline accumulation, alterations in carbohydrate levels, elevated osmotically relevant metabolites, and modifications in the TCA cycle, indicating significant metabolic adaptation [5, 22, 23]. Although these studies provide important insights into the physiological response to simultaneous hypoxia and salt stress, they have only been a limited number of studies on the transcriptional level [7]. The investigation of combined salt-hypoxia conditions using a naturally tolerant species like *Salicornia europaea*, which occurs in salt coastal marshes, can reveal insights and enhance our understanding of these conditions. This species serves as an ideal model plant for studying adaptive mechanisms to combined hypoxia and salt stress, as demonstrated by our previous research on selected transcriptional responses under simultaneous salinity and flooding, as well as sequential salinity and hypoxia [16]. Notably, the expression patterns of hypoxia-responsive genes were altered under sequential salinity and hypoxia compared to individual stress treatments. These findings underscore the potential of *Salicornia europaea* as a valuable model to dissect gene regulatory mechanisms under combined hypoxia and salinity stress [16].

In the present study, we aimed to identify hypoxia-salt specific gene expression changes in hypoxia or salt related pathways in the adapted plant *Salicornia europaea* to draw physiological conclusions regarding simultaneous stress interactions. We elucidated the systemic hypoxia-salt specific gene expression changes on a larger scale *via* RNA sequencing during single and simultaneous stress in both shoot and root. Utilizing a comprehensive data analysis approach, we demonstrate that simultaneous hypoxia-salt stress induced an enhanced unique stress response. This unique reaction mainly involved altered expression patterns in already known hypoxia or salt responsive pathways. Thereby, novel additive, enhanced and divergent regulations were found. Driven by data, we focused on i) carbohydrate metabolism, ii) cellular respiration/fermentation, and iii) amino acid pathways to compare gene expression level changes and determined individual divergent, enhanced and additive effects.

## Material and methods

### Plant material and cultivation

*Salicornia europaea* seeds were obtained from a seed distributer (Rühlemann´s Kräuter und Duftpflanzen, Horstedt, Germany). The cultivation was conducted in a growth chamber under controlled conditions with 16 h light (290 µmol photons m^−2^ s^−1^) at 22$^{\circ }$C. Seeds were sown on square plates with wet filter paper (Suppl. Fig. S1A) for germination. After two weeks, the seedlings were transferred into 1.5 ml reaction tubes, half filled with solid ½ Hoagland medium (2.5 mM KNO_3_, 2.5 mM Ca(NO_3_)_4_, 0.5 mM KH_2_PO_4_, 0.5 mM MgSO_4_, 50 µM KCl, 25µM H_3_BO_3_, 2.25 µM MnCl_2_, 1.9 µM ZnSO_4_, 1.5 µM CuSO_4_, 0.05 µM (NH_4_)_6_Mo_7_O_24_, 40 µM Fe-EDTA and 0.5% (w/v) Agarose) and pre-cultivated floating in liquid, aerated 1/2 Hoagland medium (Suppl. Fig. S1B). After three weeks, plants were transferred to 50 ml reaction tubes and acclimated for 1 week in hydroponic culture containing liquid ½ Hoagland medium (main culture, Suppl. Fig. S1C). Salinity treatment started with 6 week old plants.

### Salinity and hypoxia treatment

First, six-week-old *S. europaea* plants were acclimated to salt. Therefore, continuously 100 ml NaCl (3.5 M) was added to the hydroponic culture (culture volume: 7 L) per day over a total time of 12 days to a final concentration of 500 mM. Added NaCl-solution corresponded to the evaporation of Hoagland medium in the hydroponic culture. The continuous addition was chosen to avoid a salt shock reaction and ensure salt acclimatization reactions [42]. The final concentration of 500 mM NaCl was intended to represent a clear and physiologically relevant salt stress while remaining within the known tolerance range of *Salicornia* [16]. Two days after reaching the final NaCl concentration (8 week old plants), the hypoxia treatment (1% O_2_ (v/v)) was performed where plants were kept dark to avoid photosynthetic O_2_ generation during hypoxic conditions. The starting time point was the middle of the day (8 hours of light). After 2 h of hypoxia treatment the shoots and roots were immediately frozen into liquid nitrogen, and stored at −80$^{\circ }$C. The 2 h hypoxia duration was chosen as a time point shortly after immediate reactions and before the onset of secondary or downstream effects, according to previous time series experiments [16]. Further, plants only exposed to salt or hypoxia were harvested as well as control plants not subjected to salt or hypoxia at the same time point. For every condition (control (C), salt (S), hypoxia (H) and hypoxia-salt (HS)) four samples of shoots and roots, consisting of each three plants were harvested.

### RNA sequencing

The frozen plant material was ground in liquid nitrogen and RNA was isolated as described previously [16]. Total RNA was quantified with the RNA Broad Range Assay Kits on an Invitrogen Qubit 4 Fluorometer (Thermo Fisher scientific Inc, Germany), according to manufacturer’s instructions. Additionally, the integrity of the RNA (RIN value > 8) was certified in an Agilent 2100 Bioanalyzer (Agilent Technologies Inc., Germany) using the RNA 6000 Nano Kit. For sequencing the RNA concentration was set to 40 ng/µl. Paired end sequencing was performed by Eurofins Genomics (Eurofins Genomics GmbH, Germany) including strand-specific cDNA library preparation and Illumina sequencing on the NovaSeq platform (2 x 150 sequence mode). The raw RNA-Seq data sets from this study can be accessed through NCBI under Bioproject ID PRJNA1256208 for shoot data and PRJNA1256210 for root data. To confirm RNA sequencing, real time quantitative PCR (RT-qPCR) was performed, therefore samples were processed as described in Jordine et al. [16]. Oligonucleotides used for RT-qPCR are listed in Table S1. RT-qPCR was performed as described previously [16] and evaluated according to $2^{-\Delta \Delta C_T}$ method [43].

### Raw RNA sequence processing

Raw sequencing data was processed *via* the Anaconda v23.11.0 environment (Suppl. Fig. S2) [44]. Quality control of the raw reads was performed with FastQC v0.12.1 [45], then each sample was cleaned from low-quality reads and adapter fragments (Trimmomatic v0.39) [46]. The alignment to reference transcripts [16] was performed with HISAT2 v2.2.1 [47]. SAM files were converted into BAM files (SAMtools v1.13) [48] and featureCounts v2.0.1 was used to summarize the counts of all samples into one read count table [49]. All downstream analysis were performed in RStudio v4.3.3 [50]. A schematic depiction of further analysis steps can be found in Supplemental Fig. S3, with detailed session information in the Supplemental Fig. S4. For further analysis the read count table was filtered for genes with reads in at least 3 out of the 4 replicates. Mercator4 [51] was used for annotation of the reference transcriptome. Subsequently gene isoforms were cumulated into SuperTranscripts [52, 53] which were used for the alignment of the reads.

### Sample similarity and differential analysis

The differences between RNA sequencing samples are primary driven by the genes with the highest counts, as they exhibit the most significant absolute differences between samples. Therefore regularized-logarithm transformation (rlog transformation) DESeq2 v1.42.1 [54] was applied on the reads to analyze the sample similarity. A principal component analysis from the individual shoot and root data set and the combined data set was performed. For the differential expression between the conditions, counts were normalized using the median of ratios normalization (DESeq2 v1.42.1). Pairwise-comparison was then conducted with the control samples (normoxia without salt) as reference. The *p*-values were Benjamini-Hochberg corrected [55] with a statistical cutoff for false discovery rate of $\alpha =0.05$. Subsequently the log2FoldChange (log2FC) of genes with high dispersion was corrected using apeglm [56]. Finally, a gene was classified as differentially expressed gene (DEG) if the adjusted *p*-value was < 0.01 (significant DEG, sDEG). The Venn analysis (VennDiagramm v1.7.3) was conducted based on sDEG sets from salt, hypoxia and hypoxia-salt with additionally abs(log2FoldChange) > 2. All sDEGs identified through variance and Venn analyses were merged into a gene list (Suppl. Table 2). This combined list served as the basis for a non-targeted analysis aimed at uncovering unknown patterns and pathways.

### Gene categorization and pathway analysis

Functional categorization of the genes was conducted with Mercator4 v2.0 [51]. Afterwards, MapMan was used for pathway analysis and visualization of DEGs [57]. A Wilcoxon rank-sum test, followed by Benjamini–Hochberg correction [55] for *p*-values, was applied to determine whether the combined expression values in a specific functional BIN, which describe biological contexts, differ significantly from the expression changes observed in the collection of genes from all other BINs.

### Analysis of additive, enhanced and divergent effects

To determine the additive effect for each gene, we analyzed the relationship between the observed log2FoldChanges under simultaneous hypoxia-salt (HS) and the combined effect of salt stress (S) and hypoxia (H). First, log2FC values were converted into linear FoldChanges ($FC = 2^{log2FC}$ and $FC = (-1)\cdot 2^{abs(-log2FC)})$ and the expression changes in percent ($pEX$) were calculated, by adding one to negative FoldChanges or subtracting one from positive FoldChanges ($pEX :=-FC+1$ and $pEX: =+FC-1$). To quantify the additive effect of S and H, the percentages of these conditions were summed ($pEX_{{\textbf {H+S}}}=pEX_{{\textbf {H}}}+pEX_{{\textbf {S}}}$). Genes were classified based on their percentage HS change relative to the sum of S and H percentages as i) additive if within ±0.5 of this sum: $pEX_{{\textbf {HS}}}$ $\in$ [$pEX_{{\textbf {H+S}}}-0.5$, $pEX_{{\textbf {H+S}}}+0.5$] ii) enhanced if greater than this range: $pEX_{{\textbf {HS}}}$ > $pEX_{{\textbf {H+S}}}+0.5$ or iii) divergent if smaller than this range: $pEX_{{\textbf {HS}}}$ < $pEX_{{\textbf {H+S}}}-0.5$. For visualization the percentage changes were transformed back to linear FoldChange ($FC= pEX_{{\textbf {H+S}}}\pm1$) and finally log2FoldChange ($log2(FC)$). Log2FoldChange of HS was plotted against the log2FoldChange of the sum of S and H, enhanced and divergent genes were highlighted. We selected for initial analysis known hypoxia or salt-responsive pathways (amino acid metabolism, carbohydrate metabolism and cellular respiration). In addition to the pathways known to be involved, all pathways with a high probability of involvement under at least one of the conditions were also examined. Subsequently, a list of all enhanced and divergent genes was created from this analysis.

## Results

### Gene expression patterns from single and simultaneous hypoxia-salt stress cluster distinctly

For investigation of the fast transcriptional effects of *Salicornia europaea* during simultaneous 2-hour hypoxia combined with/without salt-adaptation, we conducted an RNA sequencing analysis on shoot and root material. RNA sequencing yielded 28–34 million cleaned reads per shoot sample, which were aligned to the reference transcriptome [16] with an alignment rate of 63–69%. For root samples, RNA sequencing resulted in 17–26 million cleaned reads and a slightly reduced alignment rate of about 54–63% (Suppl. Fig. S5). The data set resulted in a total of 13086 shoot and 13231 root genes with open reading frames, excluding isoforms (Suppl. S6A). Out of these, we successfully annotated 9405 shoot and 9524 root genes (Suppl. Fig. S6B).

**Fig. 1:**
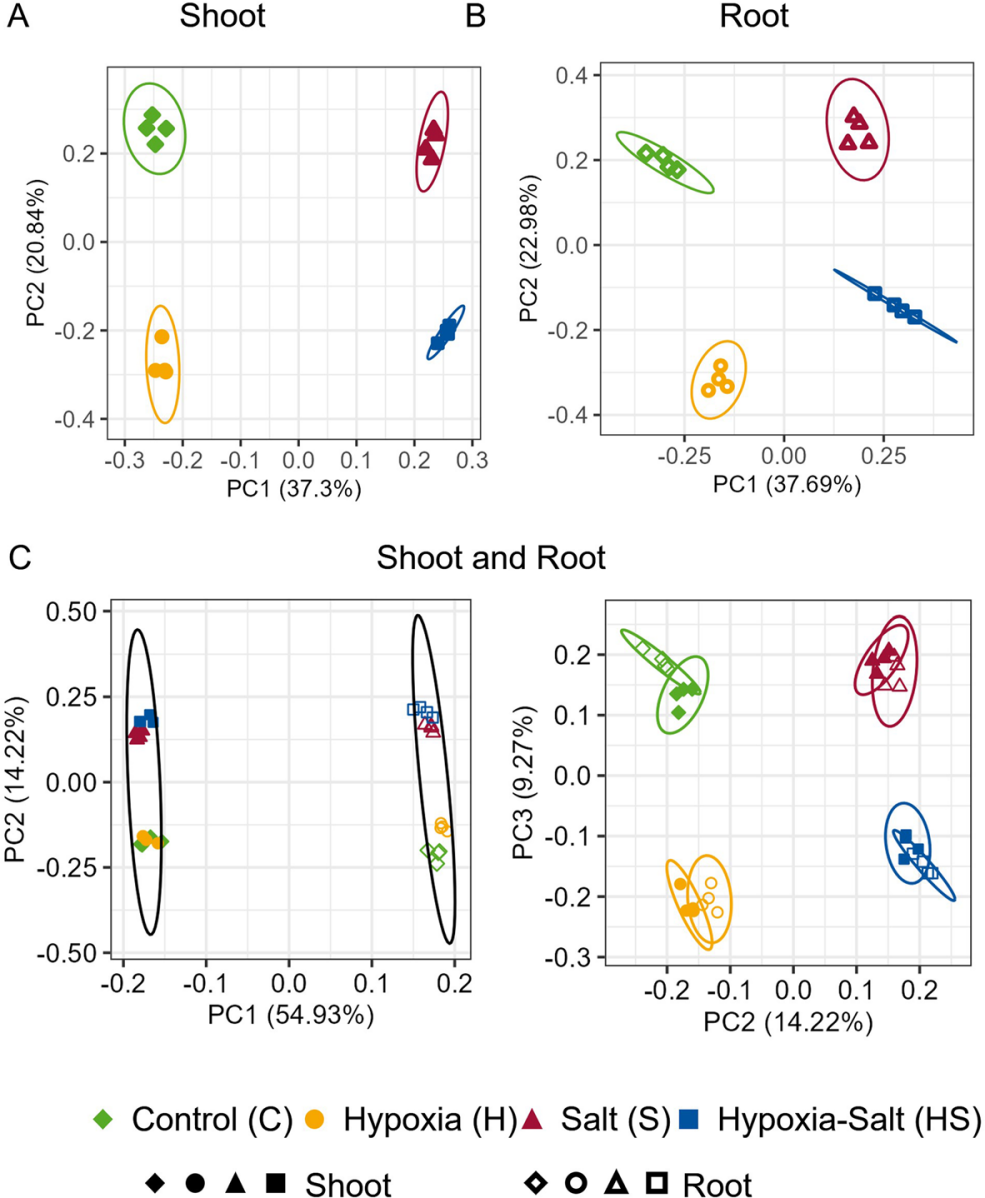
Principle Component Analysis (PCA) of All Annotated Genes Following RNA Sequencing. Counts were transformed using regularized log (rlog) transformation, analyzed separately for (**A**) shoot and (**B**) root samples and (**C**) their combined dataset. Different experimental conditions are depicted by distinct symbols and colors- filled shapes: shoot samples; empty shapes: root samples; green-diamond: control; yellow-cycle: hypoxia; red-rectangle: salt; blue-square: hypoxia salt

Principal Component Analysis (PCA) of annotated genes revealed four distinct clusters based on conditions for both shoots (Fig. 1A) and roots (Fig. 1B). In both shoot and root, a clear separation of the stress conditions was determined, with a variance explained for shoot of ~58% and root ~61% in principal components 1 (PC1) and 2 (PC2). In both PC1 (> 37%), plants treated with salt (red-triangles and blue-squares) were clearly separated from non-salt-treated ones (green-diamonds and yellow-cycles). In both PC2 (> 20%) normoxic and hypoxic conditions were separated. This separation persisted when non-annotated genes were included in the analysis (Suppl. Fig. S7). For all four conditions, only a small variation was detected between the biological replicates. Combined analysis of shoot and root data showed clear separation by PC1 (> 54%), distinguishing root data (empty shapes) from shoot data (filled shapes) (Fig. 1C left, PC1-PC2) and PC2 separated again salt treatment. Additionally, analysis of PC2 and PC3 (23%, Fig. 1C right) clustered the conditions similar to single data sets. Overall, ~78% of the variance could be explained by PC1, PC2 and PC3 in this combined analysis. These findings demonstrate that the combination of two stress factors lead to a distinct gene expression pattern compared to single stress treatments, with clear separation observed not only between stress conditions but also between plant organs.

### Hypoxia-salt stress drives extensive reprogramming in various metabolic pathways

**Fig. 2:**
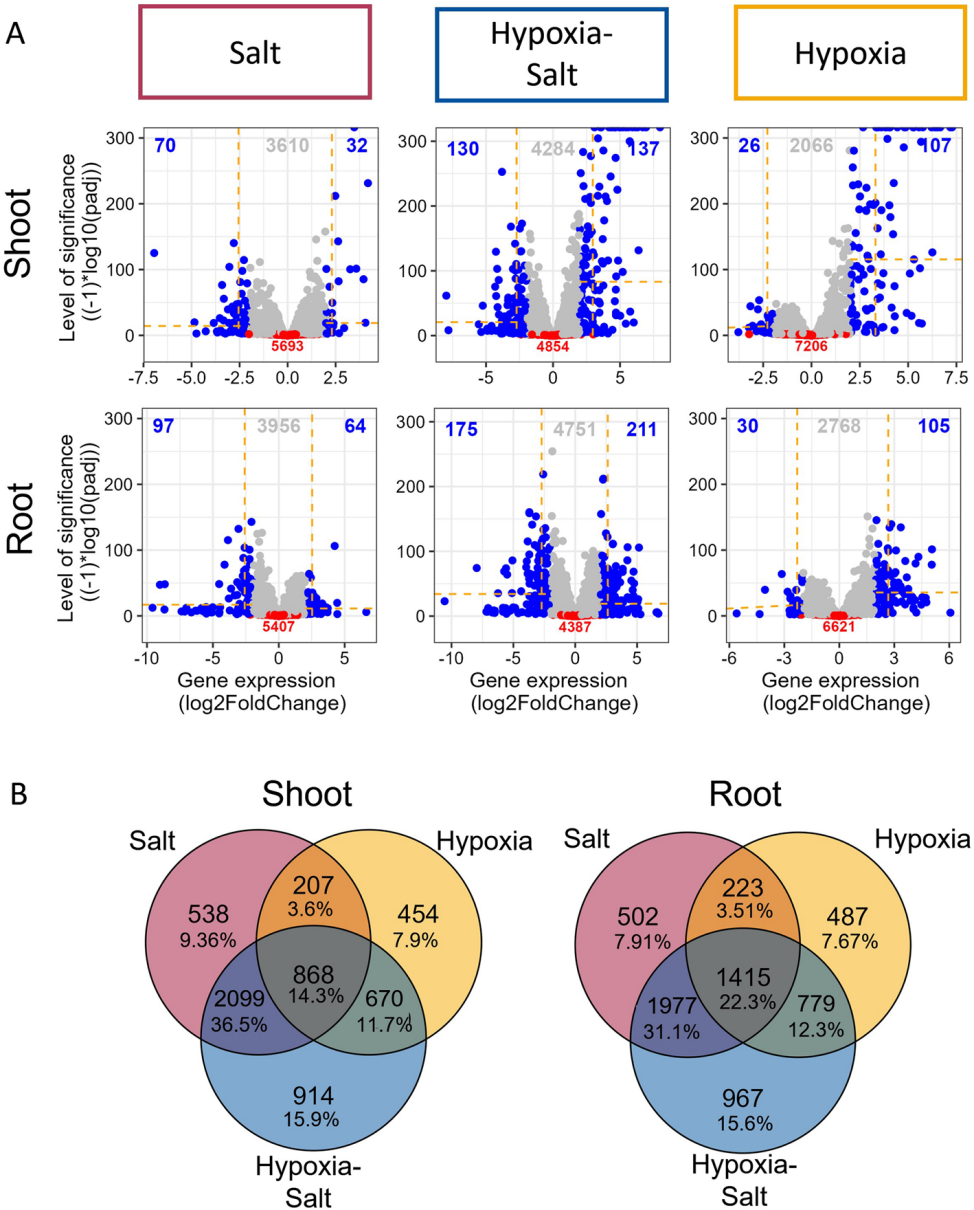
Differential Gene Expression Analysis under Salt, Hypoxia-Salt and Hypoxia in Shoots and Roots. **A** The log2FoldChanges (log2FC) of gene expression for shoots and roots were plotted against their significance level (-log10 of the adjusted *p*-values) across different conditions in both tissues. Blue dots: significant differentially expressed genes (sDEGs) with high log2FC values (*p*-value < 0.01 and $abs(log2FC)> 2$). Grey dots: sDEGs with a lowered log2FC (*p*-value <0.01 and $abs(log2FC) < 2$). Red dots: genes without significant changes (*p*-value > 0.01). Colored numbers indicate the number of genes in their corresponding category. Yellow dashed lines mark the median of the gene expression change (log2FC, vertically) and the median of the significance level ($$\begin{aligned} \left(-1\right)*log10\left(padj\right) \end{aligned}$$, horizontally) for highly regulated genes (blue dots). **B** The overlap of sDEGs (significant differentially expressed genes, *p*-value < 0.01) among individual hypoxia, salt, and simultaneous hypoxia-salt stress. Each circle represents the sDEGs set for one condition, with overlapping areas indicating shared genes between conditions, while unique genes are shown in non-overlapping sections. Abbreviations: abs:= absolute; log2FC:= logarithmic fold change in gene expression; DEGs:= differentially expressed genes; sDEGs:= significant differentially expressed genes; *p*-value:= significance value; padj:= adjusted *p*-value

In order to investigate how combined exposure to hypoxia-salt reprogram gene expression compared to individual stress conditions, differential gene expression analysis was conducted. Genes were classified as significantly differentially expressed (sDEG) relative to control samples if they had an adjusted *p*-value < 0.01 (see Material and methods, sDEGs). Across all conditions, roots exhibited a higher total number of sDEGs compared to shoots (Fig. 2A, blue and grey numbers, Suppl. Fig. 6B). Simultaneous hypoxia-salt treatment yielded the highest number of sDEGs, with 3370 genes affected in shoots and 3877 in roots, surpassing other conditions. Subsequently, these substantial numbers indicate extensive gene reprogramming under hypoxia-salt, as approximately half of the genes were significantly changed (in shoots ~48% and in roots ~53%). For the individual stresses, salt affected 39-30% (shoot-root), while hypoxia influenced 23–43% (shoot-root) genes (Suppl. Fig. S6B, Fig. 2A, blue and grey numbers). In addition, comparison of up-regulated versus down-regulated transcripts revealed nearly balanced numbers, with slightly more up-regulation under hypoxia-salt and hypoxia treatments (Suppl. Fig. S6B). Subsequently, comparing numbers of highly up- and down-regulated gene expressions ($abs(log2FC)> 2$ ) revealed a higher number of up-regulation in hypoxia-salt and hypoxia compared to down-regulation (Fig. 2A, blue).

Genes exhibiting high log2FCs in their expression were analyzed and filtered from the data (Suppl. Table 2, Sheet ‘Volcano_highly_regulated_DEGs’, Suppl. Figs. S8 and S9). In both tissues during hypoxic conditions (H, HS), highly up-regulated transcripts included genes like *SUCROSE SYNTHASE (SUS), ETHYLENE RESPONSIVE FACTORS group VII* (*ERFVII; RAP2.12*), *PLANT CYSTEINE OXIDASE (PCO) *and* PYRUVATE DECARBOXYLASE (PDC)* (Suppl. Figs. S8 and S9). During salt treatments (S and HS), *SUGAR WILL EVENTUALLY BE EXPORTED TRANSPORTER (SWEET)* paralogs exhibited both strong up-regulation and down-regulation across tissues. Under hypoxia (H) alone *SWEETs* were not impacted severely in expression changes (Suppl. Figs. S8 and S9). Further carbohydrate metabolism related genes varied in expression based on tissue type and condition. For example, *GLUCOSE-6-PHOSPHATE DEHYDROGENASE (G6PDH)* and *ALDEHYDE DEHYDROGENASE 2 (ALDH2)* were strongly down-regulated in their expression under saline conditions (S and HS) in shoots. Additionally we confirmed the expression pattern of selected hypoxia and salt responsive genes *via* RT-qPCR (Suppl. Figs. S16 and S17) and compared them to the RNA sequencing results. All tested genes showed similar expression patterns between RT-qPCR and RNA sequencing analysis.

Under simultaneous stress (HS) paralogs of *TREHALOSE-6-PHOSPHATE PHOSPHATASE (TPP)* displayed both high up- and down-regulation in the gene expression of the shoots, and were up-regulated in roots. *VACUOLAR/CELL WALL INVERTASE INHIBITOR (VIF2)* expression was down-regulated in shoots and *FRUCTOSE-1,6-BISPHOSPHATE ALDOLASE (FBA1)* expression was up-regulated in roots under simultaneous stress. Independent of tissue or conditions, the expression of branched-chain amino acid degradation genes were down-regulated, with the exception that no amino acid-related gene was highly regulated in shoots under hypoxic conditions. Genes involved in GABA (gamma-aminobutyric acid) and methionine degradation were down-regulated under simultaneous hypoxia-salt in both tissues, as well as under salinity (S) alone in the roots. Interestingly, *GLUTAMATE DECARBOXYLASE (GAD)* (Suppl. Figs. S8 and S9) a gene involved in the biosynthesis of GABA was up-regulated in the shoots under hypoxic conditions (H and HS), and in the roots under simultaneous hypoxia-salt.

Additionally, in regard of energy metabolism, a hypoxia specific up-regulation of *SUCROSE NON-FERMENTING-1-RELATED PROTEIN KINASE 3* (*SnRK3*) was observed in shoots (H and HS), and in roots under H. Overall, the reprogramming after simultaneous hypoxia-salt treatment revealed the highest significant changes in differential gene expression patterns independent of the examined tissue, indicating a unique effect that enhances the simultaneous response compared to each condition alone (Fig. 2A, grey and blue). To evaluate whether simultaneous stress affects specific functional categories, we conducted an enrichment analysis using MapMan across all metabolic pathways [57]. Enriched categories represent metabolic pathways that are over- or under-represented in experimental conditions compared to a reference group. Key enriched categories (*p*-value < 0.05) in shoots under all conditions included i) coenzyme metabolism, ii) photosynthesis, iii) protein biosynthesis, and iv) vesicle trafficking (Suppl. Fig. S10). Under hypoxia-salt conditions, no unique gene categories were significantly enriched beyond those impacted by individual stresses in both shoots and roots. Notably, protein modification and secondary metabolism were enriched in shoots under single stressors but not combined stress, suggesting potential divergent effects. In roots hypoxia enriched categories were not enriched under simultaneous hypoxia-salt, while all salt enriched categories were also enriched under simultaneous hypoxia-salt.

Next we explored the extent to which the differential gene expression under simultaneous stress conditions reflects the combined effects of individual stresses (Fig. 2B). Overall, ~14% (868 sDEGs) of shoot genes and ~22% (1415 sDEGs) of root genes showed significant changes regardless of the conditions type (Fig. 2B, total overlap). As expected due to experimental setup and PCA analysis (Fig. 1) in shoots and roots, salt treatments (S, HS) shared the highest proportion of sDEGs, with 2099 shoot genes (~36%) and 1977 root genes (~31%). The overlap between hypoxia and hypoxia-salt was lower with ~12% in shoot and in root. The lowest proportion of sDEGs was shared between salt and hypoxia with ~4% from shoots and roots. Unique sDEGs proportions varied across conditions for shoot and root in i) salt ~8-9% ii) hypoxia-salt ~15-16% and iii) hypoxia ~8% (Fig. 2B). Additionally, up- and down-regulated genes were analyzed separately (Suppl. Fig. S11). As HS was most impacted, there nearly equal proportions of shoot sDEGs were either up- (~15%) and down-regulated (~15%) uniquely (Suppl. Fig. S11). In the root HS, slightly higher proportions of up-regulated genes (~17%) than down-regulated sDEGs (~12%) were found to be unique for hypoxia-salt (Suppl. Fig. S11). Taken together, hypoxia-salt (HS) led to the most unique significantly differentially expressed genes, highlighting that the combination of both conditions impacts gene expression distinctly.

### Hypoxia-salt responses can be driven by enhanced but also divergent effects of hypoxia and salt responsive pathways

To investigate whether the combined hypoxia-salt response arises from enhanced or divergent interactions, we compared transcriptional changes under single and combined stress conditions.

We analyzed how the summed individual stress responses equal the combined hypoxia-salt changes ($log2FC(H+S) = log2FC(HS)$). Deviations from expected additive outcomes were classified as either enhanced or divergent effects. To evaluate whether these effects are predominantly influenced by uniquely hypoxia-salt specific genes or highly expressed genes, we examined their respective proportions.

**Fig. 3:**
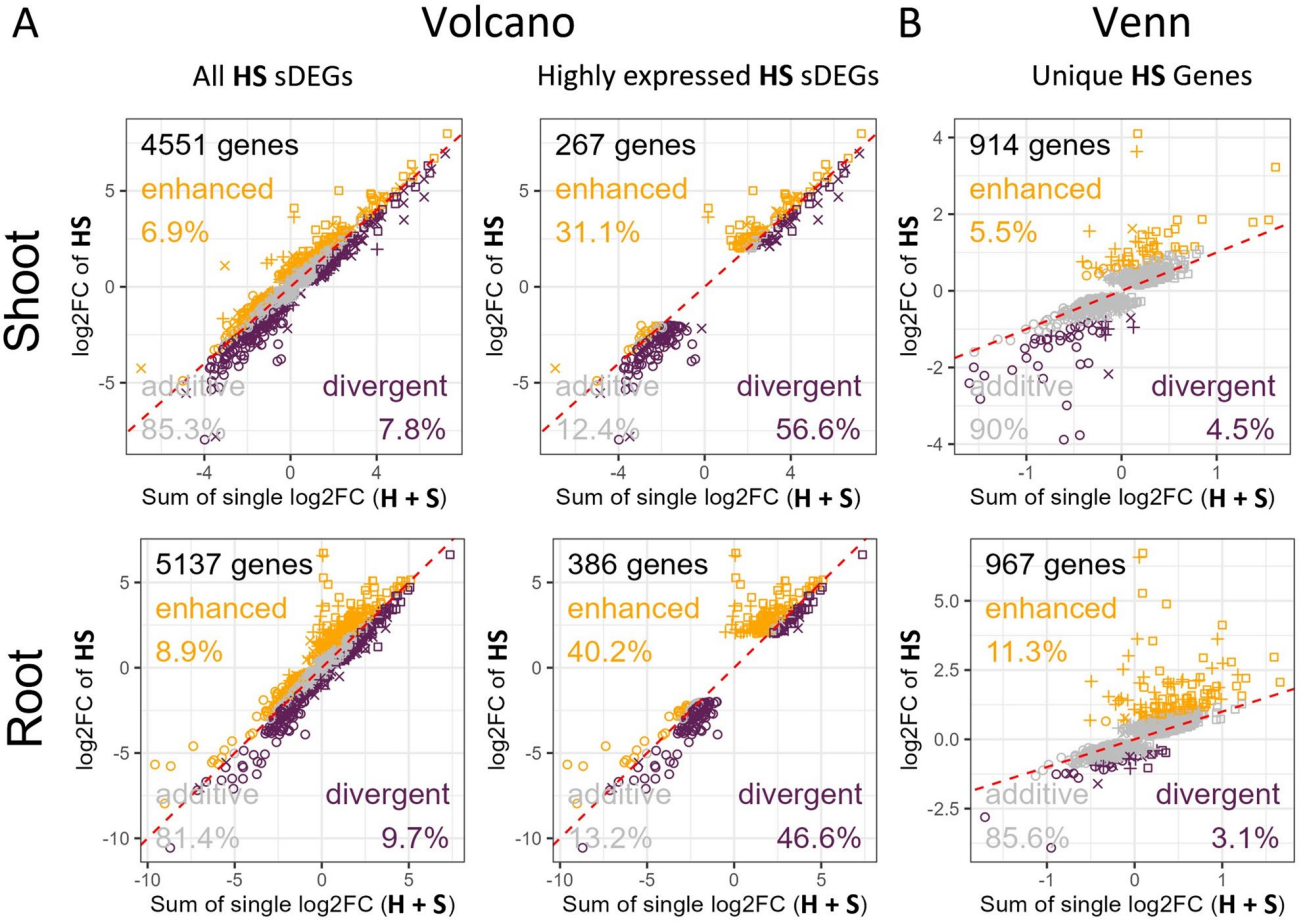
Deviation of Significant Gene Expression Responses from Additive Effects Under Combined Hypoxia-Salt Stress. The relationship between the summed log2FoldChanges (log2FC) of individual stress responses (salt and hypoxia) and the log2FC under simultaneous hypoxia-salt (HS) stress in both tissues for (**A**) all significant differentially expressed genes (sDEGs) and highly expressed sDEGs of the hypoxia-salt response (Volcano analysis, Fig. 2A grey and blue dots) as well as for (**B**) uniquely (only in HS) sDEGs hypoxia-salt genes (Venn analysis, Fig. 2B). Grey markers denote genes with additive effects, while orange and violet markers indicate genes with enhanced or divergent effects, respectively. Additive effects were defined as follows: when the sum of individual stress responses matches the HS response $FC(HS)=FC(H+S)\pm FC(0.5)$ confidence interval). Enhanced effects were defined when HS expression levels exceed the sum by at least 0.5 FC, and divergent effects, when HS was below the sum by 0.5 FC threshold. The red diagonal denotes the expected trend for additive responses. Symbols represent the sign of log2FC of the individual stress (circle: both (H and S) negative; square: both (H and S) positive; +: H negative and S positive; $\times$: H positive and S negative). Abbreviations: FC:= Fold change; DEGs:= Differentially expressed genes

In the volcano plot analysis of hypoxia-salt related significant differentially expressed genes (sDEGs) (Fig. 3A), over 82% were classified as additive in both shoots and roots. enhanced and divergent effects each represented ~7% of sDEGs in shoots, and in roots these proportions increased slightly to ~9%. Interestingly, highly expressed genes showed predominant enhanced or divergent effects, collectively accounting for over 85% of sDEGs across both tissues. In contrast, genes uniquely expressed under hypoxia-salt stress (Fig. 3B) exhibited less than 15% involvement in enhanced or divergent expression within either tissue type.

**Fig. 4:**
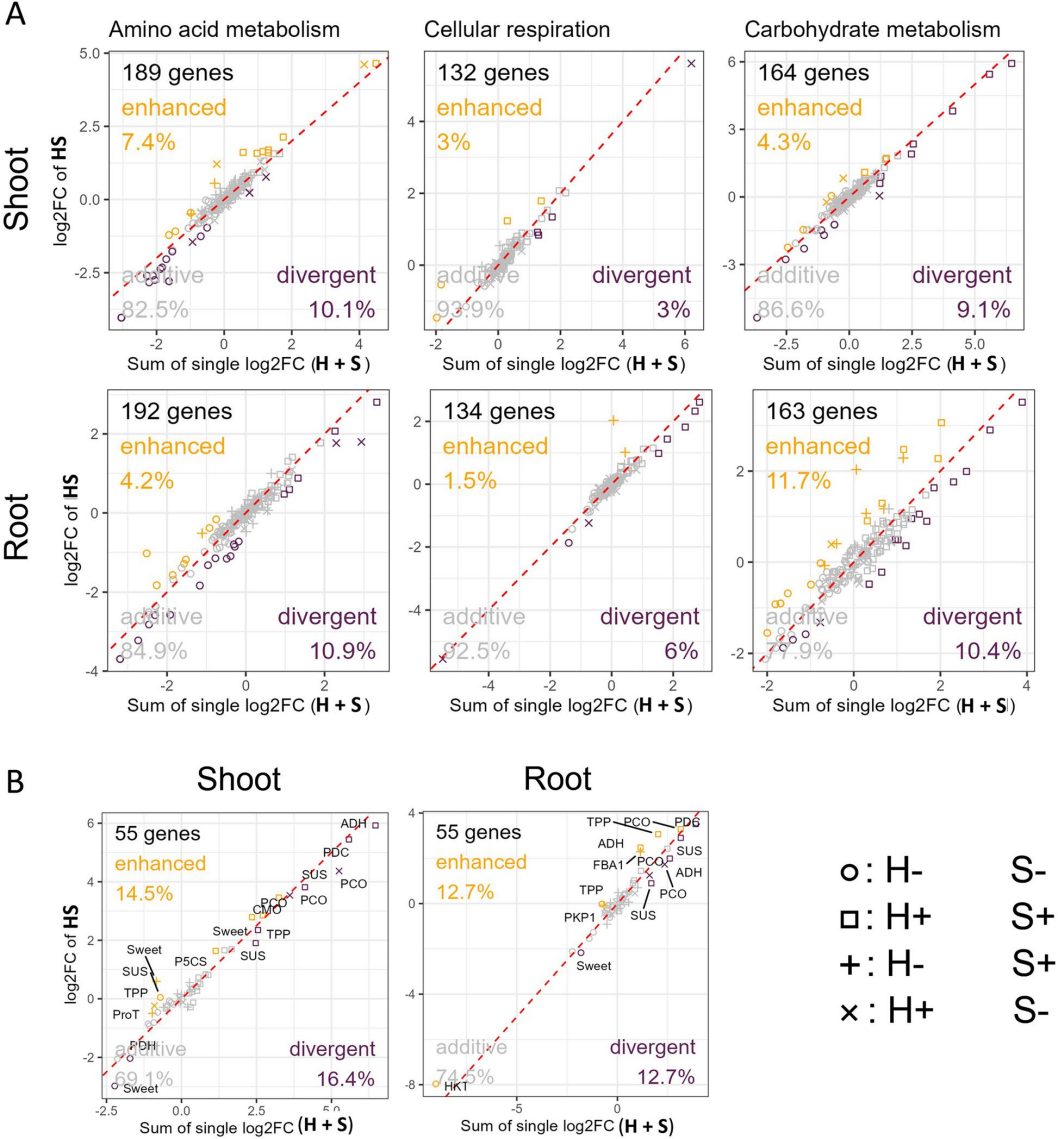
Deviation of Significant Gene Expression Responses from Additive Effects Under Combined Hypoxia-Salt Stress in Hypoxia and Salt Responsive Pathways. The relationship between the summed log2FoldChanges (log2FC) of individual stress responses (salt and hypoxia) and the log2FC under simultaneous hypoxia-salt (HS) stress in both tissues for genes of (**A**) amino acid metabolism, carbohydrate metabolism, and cellular respiration pathways as well as for (**B**) known 56 genes to be involved in hypoxia or salt responses [26, 58, 59] (Suppl. Table 2 Sheet ‘AddEff_HRG_SRG’). Grey markers denote genes with additive effects, while orange and violet markers indicate genes with enhanced or divergent effects, respectively. Additive effects were defined as follows: when the sum of individual stress responses matches the HS response $FC(HS)=FC(H+S)\pm FC(0.5)$, confidence interval). enhanced effects were defined when HS expression levels exceed the sum by at least 0.5 FC, and divergent effects, when HS was below the sum by 0.5 FC threshold. The red diagonal denote the expected trend for additive responses. Symbols represent the sign of log2FC of the individual stress (circle: both (H and S) negative; square: both (H and S) positive; +: H negative and S positive; $\times$: H positive and S negative). Abbreviations: FC:= Fold change; PCO:= Plant cysteine oxidase; PDC:= Pyruvate decarboxylase complex; TPP:= Trehalose phosphatase; SUS:= Sucrose synthase; Sweet:= sugars will eventually be exported transporters; CMO:= Choline monooxygenase; P5CS:= Pyrroline-5-carboxylate synthase; ProT:= Proline transporter; LDH:= Lactate dehydrogenase; FBA:= Fructose-1,6-bisphosphate aldolase; PK:= Pyruvate kinase; HKT:= Potassium/sodium cation transporter

Only a small number (< 30) of shoot- and root-specific uniquely sDEGs were classified as highly differentially expressed enhanced or divergent genes (Examples in: Suppl. Figs. S12 and S13, Suppl. Table 2, Sheet ‘Overlap_highVolcano_UniqueHS’), with most exhibiting low expression levels (counts) and high variance across the four replicates. Most key drivers behind the hypoxia-salt response were also identified as sDEGs in one or both individual stress treatments (Suppl. Table 2, Sheet ’Overlap_highVolcano_UniqueHS’). These genes participate in pathways responsive to hypoxia or salt, such as amino acid metabolism, cellular respiration, and carbohydrate metabolism. Therefore we selected these metabolic routes to unravel the enhanced and divergent impact in depth. The majority of data points (> 80%) cluster near the additive response (Fig. 4A, dashed red line). Cellular respiration exhibited the highest additive values in both shoot and roots (> 92%), while shoot amino acid metabolism (> 82%) and root carbohydrate metabolism (> 77%) had the lowest. Genes with divergent expression levels were proportionally higher or at least equal compared to those with enhanced effects. In shoots, gene expression patterns separated as follows: i) amino acid metabolism with ~7% enhanced versus ~10% divergent, ii) carbohydrate metabolism with ~4% enhanced versus ~9% divergent, and iii) cellular respiration with ~3% enhanced versus ~3% divergent. In roots, the patterns revealed a greater divergent influence compared to shoots. Root gene expression were distributed for i) amino acid metabolism with ~4% enhanced and ~11% divergent, ii) carbohydrate metabolism with ~12% enhanced ~10% divergent and iii) cellular respiration with ~2% enhanced and ~6% divergent. We then examined 57 genes or gene families specifically known to respond to hypoxia (26 genes) or salt stress (31 genes, [26, 58, 59], Suppl. Table 2, Sheet ‘AddEff_HRG_SRG’). From these, shoots exhibited both stronger enhanced and divergent responses compared to roots (Fig. 4B). Specifically, ~15% of hypoxia- or salt-responsive genes showed enhanced effects in shoots, compared to ~13% in roots. Divergent responses were slightly more pronounced in shoots (~16%) than in roots (~13%). These findings underscore that the hypoxia-salt response involves complex interactions between stress pathways, resulting in unique transcriptional outputs that defy predictions based solely on individual stress conditions.

### Simultaneous hypoxia-salt alters central metabolic pathways

**Fig. 5:**
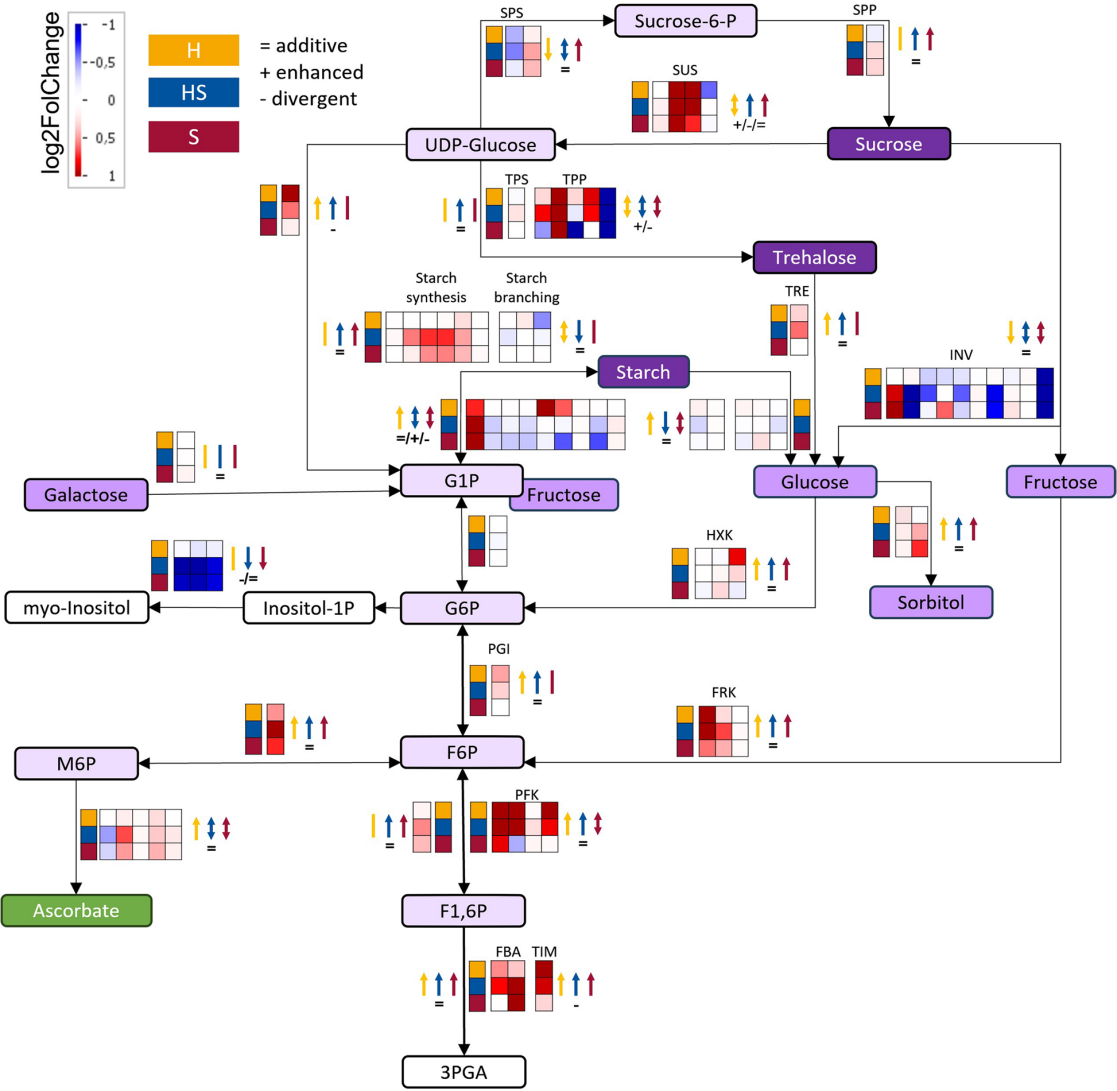
Carbohydrate Pathway Specific Transcriptional Expression Changes Under Hypoxia, Hypoxia-Salt and Salt in *S. europaea*. Expression changes (log2FC) are displayed on a custom pathway using the MapMan tool [57]), an assembled reference transcriptome was annotated using Mercator and MapMan as reference. Sugar phosphates, monosaccharides and di-/polysaccharides were marked in light to dark violet, respectively. Organic acids were highlighted in green. Black arrows between the metabolites display the enzymatic conversion. Changes in the transcripts encoding these enzymes are indicated in the boxes next to the linking arrow with positive (red) and negative (blue) log2FC. The squares indicate conditions: yellow-H: hypoxia, blue-HS: hypoxia-salt, and red-S: salt. Color coded arrows indicate up- or down-regulation of expressions for the color coded respective condition together with indications of additive (=), enhanced (+) and divergent (-) HS responses. If gene expressions were up- and down-regulated, two-headed arrows were used. Abbreviations: 3PGA:= 3-Phosphoglyceric acid; F6P:= Fructose-6-phosphate; F1,6P:= Fructose-1,6-bisphosphate; FBA:= Fructose-bisphosphate aldolase; FRK:= Fructokinase, G1P:= Glucose-1-phosphate; G6P:= Glucose-6-phosphate; HXK:= Hexokinase, Inositol-1P:= Inositol-1-phosphate; INV:= Invertase; M6P:= Mannose-6-phosphate; PGI:= Phosphoglucose isomerase; PFK:= Phosphofructokinase; SUS:= Sucrose synthase; Sucrose-6-P:= Sucrose-6-phosphate; SPP:= Sucrose phosphate phosphatase, SPS:= Sucrose phosphate synthase; TIM:= Triose-phosphate isomerase; TPS:= Trehalose-6-phosphate synthase, TRE:= Trehalase; TPP:= Trehalose-6-phosphate phosphatase

The previously uncovered enhanced and divergent responses to hypoxia and salt in shoot primary metabolic pathways were deeper examined. Carbohydrate metabolism analysis (Fig. 5) revealed transcriptional adjustments in sucrose and starch degradation, as well as trehalose metabolism.

**Fig. 6:**
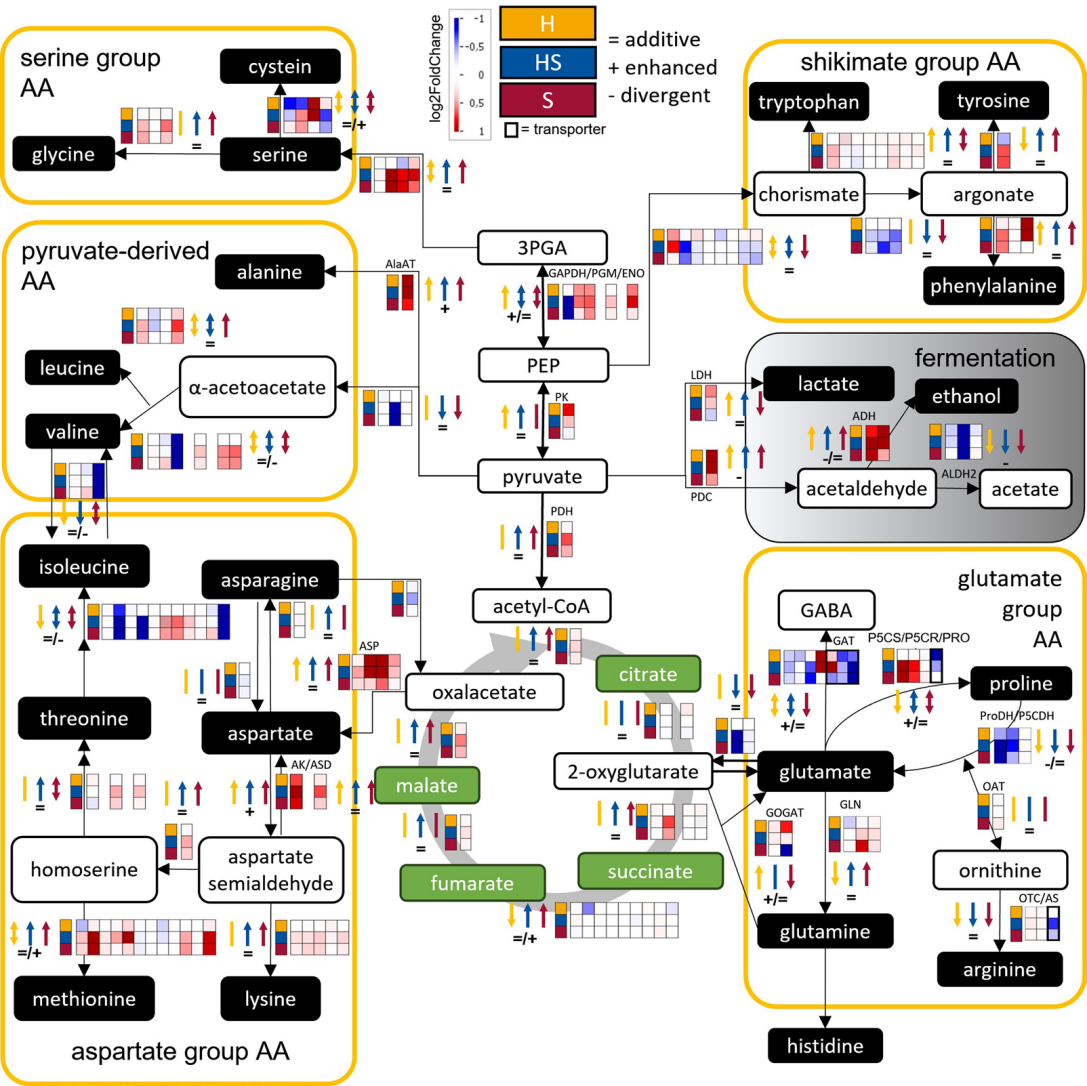
Glycolysis Parts, Tricarboxylic Acid (TCA) Cycle, Fermentation and Amino Acid Metabolism Specific Transcriptional Expression Changes Under Hypoxia, Hypoxia-Salt and Salt in *S. europaea* Shoots. Expression changes (log2FoldChange, log2FC) are displayed on a custom pathway using the MapMan tool, an assembled reference transcriptome was annotated using Mercator and MapMan as reference. Organic acids (green), amino acids and fermentation products (black) and other metabolites (white) are depicted in their metabolic routes. The yellow boxes summarize all amino acids and conversions of the respective amino acid group. Black arrows between the metabolites display the enzymatic conversion. Changes in the transcripts encoding these enzymes are indicated in the boxes next to the linking arrow with positive (red) and negative (blue) log2FC. The squares indicate conditions: yellow-H: hypoxia, blue-HS: hypoxia-salt, and red-S: salt). Color coded arrows indicate up- or down-regulation of expressions for the color coded respective condition together with indications of additive (=), enhanced (+) and divergent (-) HS responses. If genes were up- and down-regulated, two-headed arrows were used. Abbreviations: 3PGA:= 3-phosphoglyceric acid; AA:= Amino acids; acetyl-CoA:= Acetyl-coenzyme A; ADH:= Alcohol dehydrogenase; AlaAT:= Alanine Aminotransferase; ALDH2:= Aldehyde dehydrogenase; AK:= Aspartate kinase; AS:= Argininosuccinate synthase; ASD:= Aspartate-semialdehyde dehydrogenase; ASP:= Aspartate aminotransferase; ENO:= Enolase; GABA:= Gamma-aminobutyric acid; GAT:= GABA transporter; GAPDH:= Glyceraldehyde 3-phosphate dehydrogenase; GDH:= Glutamate dehydrogenase; GLN:= Glutamine synthetase; GOGAT:= Glutamate synthase; LDH:= Lactate dehydrogenase; OAT:= ornithine aminotransferase; P5CS:= Pyrroline-5 carboxylate synthetase; P5CR:= Pyrroline-5 carboxylate reductase; P5CDH:= Pyrroline-5 carboxylate dehydrogenase; PDC:= Pyruvate decarboxylase; PDH:= Pyruvate dehydrogenase; PEP:= Phosphoenolpyruvate; PGM:= Phosphoglycerate mutase; PK:= Pyruvate kinase; ProT:= Proline transporter; ProDH:= Proline dehydrogenase

During hypoxia (H) expression of *INVERTASE* (*INV*) was down-regulated, whereas *SUCROSE SYNTHASE* (*SUS*) was up-regulated (Fig. 5, yellow). Salt stress (S) exhibited similarly higher *SUS* and a bit more varied *INVs* patterns, and additional expression up-regulation linked to starch synthesis (Fig. 5, red). Reduced expression of starch branching enzymes suggested putative enhanced amylose formation. Combined hypoxia-salt stress (HS), led to an intensified down-regulation of the *INVs* sucrose degradation genes, coupled with stronger up-regulation of *SUS*, *TPP* and *TREHALASE* (*TRE*) (Fig. 5, blue). Additionally, three out of four *SUS* were regulated enhanced (1) and divergent (2) while the *INV*s showed primarily additive effects (8 out of 9 *INV* genes). Half of the *TPP* genes were enhanced or divergent regulated under simultaneous stress (Fig. 5, signs, Suppl. Table 2, Sheet ‘Genes_Carbohydrate_metabolism’). Additionally, starch synthesis was further enhanced alongside reduced branching enzyme genes once more indicating increased amylose formation. Amino acid (AA) metabolism (Fig. 6, Suppl. Table 2, Sheet ‘Genes_AS_metabolism’), revealed distinct transcriptional changes across different stress conditions. Salt stress (S) triggered induction of the serine-derived AA group, particularly glycine synthesis, whereas hypoxia (H) repressed this group by down-regulating cysteine synthesis genes and yet maintained expression for glycine synthesis.

Combined hypoxia-salt (HS) serine-derived genes showed additive induction. Shikimate pathway responses varied, salt stress down-regulated early biosynthetic gene expressions (not H) but up-regulated phenylalanine, and tyrosine synthesis genes (Fig. 6, red). Hypoxia up-regulated phenylalanine and tryptophan and down-regulated tyrosine synthesis genes (Fig. 6, yellow). Combined HS caused stronger changes, tryptophan followed the hypoxia H pattern (up), tyrosine the salt pattern (up), and phenylalanine showed a mixed response, resulting overall in additive expression across the shikimate group. Pyruvate-derived AA genes were only slightly affected by all treatments, except alanine biosynthesis (*ALANINE AMINOTRANSFERASE*, *AlaAT*), which is up-regulated under all conditions and valine biosynthesis, which seemed to be regulated under combined HS stress (Fig. 6, blue).

Gene expressions within the aspartate group AA metabolism - including methionine, threonine, asparagine, and lysine - were up-regulated under both salt (S) and simultaneous hypoxia-salt (HS) stress. *ASPARTATE AMINOTRANSFERASE* (*ASP*) showed strong induction under hypoxia and additive expression under combined stress, while *ASPARTATE KINASE* (*AK*) was activated by both single stresses and displayed enhanced effects under simultaneous hypoxia-salt treatment. Salt stress (S) induced higher expression patterns in the glutamate group by proline (*P5CS*) and glutamine *via* glutamate, while *GABA TRANSPORTER* (*GAT*), arginine transporter, and *PROLINE TRANSPORTER* (*ProT*) were lowered. This pattern was intensified under simultaneous HS stress. Under hypoxia, opposing patterns to salt stress were observed. Genes of the glutamate-derived group responded the most to all treatments. Salt and hypoxia showed contrasting patterns, with salt (S) causing higher expressions of *PYRROLINE-5-CARBOXYLATE SYNTHASE* (*P5CS*) and *PYRROLINE-5-CARBOXYLATE REDUCTASE* (*P5CR*), along with lowered expression of the *PROLINE TRANSPORTER* (*ProT*). Proline-degrading enzymes (*ProDH*, *P5CDH*) were repressed under all conditions, whereas the *GABA TRANSPORTER* (*GAT*) was down-regulated by salt (S) but induced by hypoxia (H). Under combined HS, expression patterns resembled those of salt but were more pronounced, with enhanced regulation of *P5CS*, *GAT*, and *ProT*.

Gene expression related to ethanol and lactate biosynthesis was generally up-regulated under hypoxic conditions (H and HS). Under salt stress *PYRUVATE DECARBOXYLASE* (*PDC*) and *ALCOHOL DEHYDROGENASE* (*ADH*) were also up-regulated, whereas *LACTATE DEHYDROGENASE* (*LDH*) was slightly down-regulated. Under simultaneous stress, *LDH* exhibited additive regulation while *PDC* and *ADH* showed divergent regulation.

## Discussion

Massive reprogramming and adaptation effects of the transcriptome were shown for hypoxia and salt individually for example in *Arabidopsis*, Tomato or Barley [26, 30, 60–64], which are sensitive to both stresses. Yet, the transcriptional reaction to simultaneous hypoxia and salt remains largely unexplored - a gap that limits our understanding of complex stress interactions in natural environments. We wanted to identify gene expression changes in a naturally hypoxia-salt adapted plant by examining enhanced, divergent, and additive effects resulting from individual hypoxia or salt treatments. We selected *Salicornia europaea*, known for thriving under fluctuating oxygen and salt levels [16], offering a model for investigating these dynamics. Our comprehensive transcriptome study characterized the gene expression profile shifts of *S. europaea* under hypoxia, salt, and simultaneous hypoxia-salt. Additionally, we aimed to deepen the understanding of the cross-talk between these two stress responses, enabling us to derive first physiological insights into interactions and adaptive mechanisms during simultaneous hypoxia-salt exposure. Although no full genome sequencing data was available for the alignment of the RNA sequencing reads during the time of analysis, a high alignment rate (shoot > 63% and root > 54%) was achieved with the transcriptome constructed on previously released data (NCBI: SRR822929, SRR823398, SRR944677, SRR944676). Nevertheless, it can not be excluded that some transcripts are not represented in this *de-novo* assembled transcriptome.

### Adapted *Salicornia* shows similar trends as non-adapted plants during single stress

Investigations on simultaneous stress has significantly increased over the last years. Gene expression studies have primarily focused on a combination of light, heat, cold, drought, and salinity [12, 65, 66]. However, the impact of combined hypoxia and salinity on gene expression was not thoroughly explored yet. In a controlled hydroponic system, we examined the gene expression response of *S. europaea* to both individual and simultaneous salt and hypoxia stress conditions. Principal component analysis (PCA) clustering (Fig. 1) along with higher number of significant differentially expressed genes (sDEGs) under simultaneous stress (Fig. 2), demonstrates that the combined conditions cause a unique stress response in shoots and roots. The PCA of the four conditions (control, hypoxia, salt, and hypoxia-salt) revealed distinct clustering patterns in both tissues (Fig. 1, A and B). Along PC1, salt-treated samples clustered distinctly from non-salt-treated samples ones (37–38%), whereas PC2 separated plants exposed to hypoxia from those not exposed (21–23%). This suggested that salinity may play a dominant role in shaping gene expression responses under combined hypoxia-salt conditions. The concept of a "dominant stressor" has been previously described [19]. Within this framework, the simultaneous stress response is mainly driven by the more severe individual stress. However, this interpretation should be approached with caution, as our experimental setup here, with *Salicornia* plants being acclimated to salt similar to nature to avoid salt-shock responses-still revealed distinct clustering patterns under combined hypoxia-salt stresses. This provides initial evidence supporting uniquely differentiated gene expression resulting from simultaneous stress.

Differential gene expression (DEG) analysis (Fig. 2A) reinforced these findings, revealing not only the highest numbers of sDEG under simultaneous stress but also a high significance ratio (Fig. S6B), indicating a broader transcriptional response. These data are in alignment with other studies on combined stress responses, with an altered expression during the combination compared to single stress application [65, 67]. Both PCA and DEG analysis emphasize the unique profile of the simultaneous hypoxia-salt stress response. The combined shoot-root PCA analysis (Fig. 1C), revealed a clear separation by tissue type. Similar separations between shoots and roots have been reported for *Populus* [68] and *Arabidopsis thaliana* [69]. This separation highlights the distinct molecular and physiological adaptations of both organs to their specialized developmental roles [70]. Differential gene expression analysis confirmed these differences (Fig. 2A), showing that roots consistently exhibited a higher number of DEGs across all treatments, emphasizing their role in environmental stress responses [71]. Under salt stress, transcriptional expression in shoots appeared broadly repressed, with more down- than up-regulated sDEGs. In contrast, hypoxia triggered a strong transcriptional activation, inducing a large set of genes (Fig. 2A). This contrasting pattern - transcriptional repression under salt stress and activation under hypoxia - aligns with findings from previous studies on salt- and hypoxia-sensitive plant species [26, 72] and is here validated in the adapted *Salicornia* plants.

### Simultaneous hypoxia-salt stress activates uniquely expressed genes

To further characterize the unique profile of simultaneous hypoxia-salt stress responses, we compared the differential gene expression for all three conditions and quantified the overlaps and distinctions (Fig. 2B). The analysis revealed a clear pattern of overlap and disjoin across individual and simultaneous conditions. The most substantial overlap of sDEGs occurred between salt (**S**) and simultaneous hypoxia-salt (HS) conditions, with ~36% shoot and ~32% root genes shared, indicating salt as the putative dominant stressor [19], partially surely due to our experimental setup to avoid salt-shock. Additionally, our analysis also identified single salt- and hypoxia-specific gene subsets (Fig. 2B). Of particular interest was the number of genes uniquely identified as sDEGs under simultaneous hypoxia-salt (HS) stress, comprising ~16% in both tissues. This subset suggests a unique metabolic answer in the gene expression upon combined hypoxia-salt stress that are not, or only to a minor not significant extent, changed under individual stress conditions.

### Simultaneous stress is not simply additive

As hypoxia-salt led to individual response in gene expression patters, we further delved into the analysis of additive, enhanced and divergent effects. We analyzed various subsets of determined HS responsive gene i) all DEGs and ii) all highly expressed DEGs (Fig. 2A) and iii) uniquely HS expressed genes (Fig. 2B).

Under simultaneous hypoxia-salt conditions, highly expressed genes predominantly exhibited enhanced or divergent behavior (Fig. 3A), contrasting with those uniquely regulated by hypoxia-salt alone (Fig. 3B). Most highly expressed hypoxia-salt-responsive genes were also differentially expressed under one or both single-stress scenarios. As a result, only six were categorized as uniquely HS and highly differentially expressed genes in shoots (Suppl. Table 2, Sheet ‘Overlap_highVolcano_UniqueHS’,Suppl. Fig. S12), these showed low expression levels (count level) with a high variation across replicates. These results implied that simultaneous hypoxia-salt responses were primarily driven by hypoxia and salt responsive genes and their pathways - highlighting amino acid metabolism alongside cellular respiration and carbohydrate metabolism, known to react in hypoxia and salt [73, 74], as crucial interaction nodes within broader regulatory networks impacted herein. Consequently, we analyzed how these categories are affected under simultaneous stress conditions.

The majority of genes (> 80%) exhibited additive effects across amino acid metabolism, carbohydrate metabolism, cellular respiration (Fig. 4A) as well as all other functional plant categories (Suppl. Fig. shoots S14 and roots S15). Nonetheless, non-additive effects - enhanced and divergent - were determined in all functional categories (< 20%, Fig. 4A). Notably, tissue-specific differences emerged: shoots tended to be stronger enhanced compared to roots. This may indicate that above-ground tissues might benefit more from coordinated transcriptional reprogramming. Conversely, divergent effects were more pronounced in roots. These findings suggest prioritization of reprogramming towards certain metabolic pathways essential to ensure adaptation and survival amidst hypoxic and saline soil conditions.

Cellular respiration, on the other hand, exhibited the lowest variation in both shoots and roots, possibly indicating a more conserved regulatory mechanism operative under simultaneous stress. Interestingly, the investigation of 55 genes and/or gene families with well-known responses to upon hypoxia or salt [26, 58, 59], (Suppl. Table 2, Sheet ‘AddEff_HRG_SRG’), revealed a lowered additive response with under 80% in both tissues (Fig. 4B). This implies simultaneous stress causes a higher proportion enhanced or divergent effects (> 20%), in genes well-known to be involved in either response to hypoxia or salt stress. In shoots, enhancement and divergence was higher compared to roots. Therefore, shoots may leverage combined hypoxia-salt stress effects to enhance adaptive processes.

Roots showed a greater proportion of divergent in comparison to enhanced responses, which may indicate their function as a primary site for salt uptake and sensing osmotic stress [75]. Moreover, a systemic signaling transduction from roots to shoots could account for generally higher non-additive effect in the shoots. For hypoxia such a signaling transduction mechanism was described in *A. thaliana* within the carbohydrate metabolism [76]. Notably prominent are genes such as *PLANT CYSTEINE OXIDASE* (*PCO*), *TREHALOSE PHOSPHATASE* (*TPP*), *SUCROSE SYNTHASE* (*SUS*) and *SUGARS WILL EVENTUALLY BE EXPORTED TRANSPORTERS* (*SWEET*) which possess multiple paralogs exhibiting distinct additive reactions - suggesting functional redundancy but also flexibility and adaptability during concurrent stresses. The presence of multiple paralogs enable plants to finely tune metabolic pathways and adapt seamlessly amidst varied environmental stresses and/or simultaneous exposures (*PCO* [77], *TPP* [78], *SUS* [79], *SWEET* [80]). In summary, simultaneous hypoxia-salt stress elicits unique gene expression responses, characterized by significant non-additive effects across multiple pathways, highlighting complex interactions beyond simple additive expectations.

### The combination of hypoxia-salt leads to enhanced distributions of carbon equivalents in shoots

Following our initial findings on the distinct gene expression patterns under simultaneous hypoxia-salt stress, we focused on detailed analysis of carbohydrate metabolism, glycolysis, the TCA cycle, fermentation, and amino acid metabolism due to their crucial roles in facilitating adaptive responses and energy distribution during environmental stress conditions. In the carbohydrate metabolism (Fig. 5), different adaptation mechanisms were determined in response to the different stress conditions. For example, gene expression changes for C-equivalents distribution pointed under saline (S and HS) toward sucrose and starch biosynthesis, while during hypoxia (H and HS) trehalose and glucose-1-phosphate (G1P) biosynthesis were enhanced. As a result, the combination in HS resulted in all of them being higher expressed (Fig. 5, blue arrows). This stress dependent storage and re-mobilization [81] seemed to clearly differ in the gene expression analysis between, salt, hypoxia and its combination in the adapted *Salicornia europaea*. Salty conditions (S and HS) led to higher gene expressions for starch synthesis in combination with lowered expression of starch branching, pointing towards an increased amylose formation. Gene expressions for sucrose formation during salt conditions seemed to be also slightly enhanced pointing towards a maintained balanced of partitioning of assimilates between starch and sucrose which is known to be dependent on environmental conditions [82]. Together with the up-regulation of *SUS* accompanied with the down-regulation of *INV* in all conditions, suggests a redistribution of carbon fluxes to enable more efficient energy supply and the usage of energy reserves under oxygen deficiency and salinity [13, 83] and to participate in intrinsic carbohydrate and energy signaling network like for the products and enzymes like HEXOKINASES [84], glucose and fructose [85]. The trend for enhanced hypoxic gene expression for the formation of trehalose showed, half of the hypoxia-salt *TPP*s were enhanced and divergent regulated. The four *TPP* of *S. europaea* displayed distinct expression pattern, and similar expression shifts were observed in wheat [86], pointing towards either a specific expression of the paralogs or a compartment specif expression of the *TPP*s. A compartment specific expression was described for *TPP* [87], but for *S. europaea* *TPP* no compartment-specificity was described yet, therefore observed reactions could rely on both compartment- and stress-specific reactions. Nevertheless, our data suggests an increased role of trehalose as a protective molecule against oxidative and osmotic damage [88] also during hypoxic and salt-hypoxic conditions in *Salicornia*. Taken together, our gene expression data for carbohydrates suggests a strong stress dependent re-mobilization and biosynthesis of storage metabolites as well as production of signaling and protective metabolites specific for each individual stress and their combination in the evolutionary adapted *Salicornia europaea*.

### Simultaneous stress divergently regulates ethanolic fermentation

Amino acid metabolism, along with connected primary pathways, also exhibited distinct expression patterns under different stress conditions (Fig. 6). Contrasting regulatory patterns were observed in the end of glycolysis (Fig. 6), indicating a condition dependent regulation. Under salt stress (S), the gene expression of *PK* (*PYRUVATE KINASE*) was not changed, while under hypoxia (H) the expression was enhanced and slightly diminished in hypoxia-salt hinting towards a salt-dependent overwriting of the hypoxic induction when both stresses co-occur. Protein PK is known to be an important mediator in the energy producing step under anaerobic respiration [89, 90]. This could indicate diminished responsive in hypoxia-salt conditions upon the presence of salt and a slightly more enhanced role of *PDH* (*PYRUVATE DEHYDROGENASE*) which was highest expressed in HS and slightly more in S. Gene expression patterns of the following TCA cycle were mainly unaffected under all of the tested conditions.

Fermentative genes were up-regulated under hypoxic conditions (H and HS), which confirms the expected shift toward anaerobic energy production when oxygen availability is limited. Induction of *PYRUVATE DECARBOXYLASE* (*PDC*) and *ALCOHOL DEHYDROGENASE* (*ADH*) under hypoxia (H) supports the activation of ethanolic fermentation, which contributes to NAD^+^ regeneration and maintenance of glycolytic ATP production [83, 91]. The lactate fermentation pathway, mediated by *LACTATE DEHYDROGENASE* (*LDH*) expression, showed a more stress-specific pattern, with activation under hypoxia but repression under salt stress. This slight down-regulation of *LDH* under salt stress alone (S) suggests that lactate fermentation may play a less prominent role when osmotic stress occurs without oxygen limitation, possibly reflecting a metabolic preference to avoid cytosolic acidification associated with lactate accumulation [92]. LDH, which is recycling the NADH produced by glycolysis in the absence of oxygen [93], and its product lactate could be important for *Salicornia* shoots, as similar mechanisms are described for waterlogged plants where leaves detoxify root lactate *via* LDH [94]. Supplementary, *LDH* expression is known to increase upon hypoxic condition in roots from crop plants like e.g. barley and other cereals [95] as well as in potato tubers [93]. Under combined hypoxia-salt stress (HS), the additive regulation of *LDH* expression together with divergent regulation of *PDC* and *ADH* indicates that fermentative pathways do not respond uniformly when multiple stresses are present. Instead, transcriptional control appears to fine-tune fermentative fluxes depending on metabolic and ionic limitations imposed by simultaneous stresses. Such differential regulation may reflect a need to balance energy production and redox homeostasis under complex environmental conditions [91, 96].

As a summary, the expression pattern of all fermentative genes under combined stress closely resembled that observed under hypoxia alone, suggesting that oxygen limitation remains the primary driver of anaerobic metabolism during simultaneous hypoxia-salt stress. Rather suspected, in this pathway, the dominant stressor is hypoxia, indicating that the general assumption about one stressor being dominant [19] as well as our analysis (Fig. 2) pointing towards salt, may be misleading, as dominance likely varies between metabolic pathways.

### Saline conditions profoundly influence amino acid gene expressions

The genes responsible for the biosynthesis of specific amino acids - such as proline, serine, glycine, valine, lysine, leucine, and isoleucine - which are known to accumulate in spinach during salt stress [97], exhibited increased expression under salty conditions in our study. This suggests under HS the regulation *via* the stressor salt, as some of them were equal or even lower expressed under hypoxia alone. Aspartate synthesis genes (*ASP*, *ASPARTATE AMINOTRANSFERASE*) were up-regulated in an additive manner under simultaneous stress, with being higher under single hypoxia compared to single salt conditions. Interestingly, genes for the derived amino acids were higher under salty conditions. Aspartate derived amino acids (like isoleucine, lysine) play key roles in regulatory signaling pathways and nitrogen assimilation [98]. Another important gene for nitrogen assimilation under stress is *ALANINE AMINOTRANSFERASE* (*AlaAT*) [99, 100], which is up-regulated under individual stress conditions and enhanced up-regulated under simultaneous stress. Their role may be central to coordinating the plants adaptive response under combined hypoxia-salt stress.

Additionally, proline metabolism gene expression was strongly up-regulated under S and simultaneous HS stress, including synthesis genes (*PYRROLINE-5-CARBOXYLATE SYNTHETASE* *P5CS* and *PYRROLINE-5-CARBOXYLATE REDUCTASE* *P5CR*) and in parallel reduced of the proline degradation genes (*PROLINE DEHYDROGENASE ProDH* and *PYRROLINE-5 CARBOXYLATE DEHYDROGENASE P5CDH*). Proline synthesis gene (*P5CS*) also had a enhanced effect under simultaneous stress (Fig. 4B), likewise also *ProT* (*PROLINE TRANSPORTER*) and *GAT* (*GABA TRANSPORTER*) were enhanced regulated under simultaneous stress. Hence, an enhanced proline accumulation under simultaneous stress is likely. Osmoprotection and reduction of oxidative damage would be assumed, as proline functions as an osmolyte and a reactive oxygen species (ROS) scavenger [101]. As a summary, single hypoxia gene expression responses were not enhanced for proline, but enhanced specifically for GOGAT (*GLUTAMATE SYNTHASE*) and one *GABA transporter* *GAT*. The enhanced *GAT*-Transporter for GABA could be interpreted as activation of the GABA shunt known to be active under hypoxia [102].

In summary, salt treatment determined the expression levels also under combined hypoxia-salt conditions.

## Conclusion

The investigation of *Salicornia europaea* under simultaneous hypoxia-salt stress reveals unique transcriptional responses shaped by complex interactions between metabolic pathways. Notably, 16% of differentially expressed genes are uniquely altered under combined stress conditions, underscoring the distinct metabolic adaptations involved. The detailed analysis revealed that enhanced and divergent interactions significantly impact gene expression changes across key metabolic pathways such as amino acid metabolism, carbohydrate metabolism, and cellular respiration. Although additive effects were predominant, non-additive interactions were particularly pronounced in pathways known for their roles in hypoxia or salt responses. Salt stress emerged as a dominant factor influencing amino acid metabolism, particularly enhancing proline synthesis for osmoprotection. Conversely, hypoxia primarily drives fermentative responses through anaerobic pathways. The combination of these stresses lead to altered carbohydrate metabolism and significant non-additive effects, highlighting adaptive mechanisms for energy distribution and resilience in *Salicornia europaea*. These findings deepen our understanding of plant tolerance to multiple abiotic stresses and provide valuable insights into the molecular basis of adaptation in this species and beyond. Future research should focus on elucidating the specific roles of uniquely differentially expressed genes and their associated proteins and metabolites under simultaneous stress conditions to further unravel these adaptive mechanisms. Additionally, exploring similar responses in other naturally tolerant species could expand our understanding of plant resilience against environmental challenges across diverse ecosystems.

## Supplementary Information

Supplementary Material 1.

## Data Availability

The datasets generated and analysed during the current study are available in the NCBI SRA repository, under Bioproject ID PRJNA1256208 (https://www.ncbi.nlm.nih.gov/sra/PRJNA1256208) for shoot data and PRJNA1256210 for root data (https://www.ncbi.nlm.nih.gov/sra/PRJNA1256210).
