# Supplementary material for: Insights into enhanced, divergent, and additive responses to single and combined hypoxia-salt stress
Source: BMC Plant Biol. 2026 Mar 27;26:642. doi: 10.1186/s12870-026-08595-7 (PMC13063762; doi:10.1186/s12870-026-08595-7)
Supplement: Supplementary file 1 — Supplementary Material 1. [file 12870_2026_8595_MOESM1_ESM.zip › Supplementary Material/Jordine et al_supplements.pdf]

Insights into Enhanced, Divergent, and Additive Responses to Single and Combined Hypoxia-Salt Stress

Angelina Jordine[1,2,*], Julia Alt[2], Christina Bonn[1], Pia Nolte[2], Joost T. van Dongen[2], and Lisa Fürtauer[1,3,*]

[1]Plant Molecular Systems Biology, Biology III, RWTH Aachen University, 52074 Aachen, Germany

[2]Molecular Ecology of the Rhizosphere, Biology I, RWTH Aachen University, 52074 Aachen, Germany

[3]Center for Computational Life Sciences, RWTH Aachen University, Germany

[*]corresponding authors

January 31, 2026

Corresponding Author – AJ, LF
RWTH Aachen University
Institute of Biology III/I
Worringer Weg 1
52074 Aachen, Germany
+49 (0) 241 80 26634
Email:
Angelina.Jordine@rwth-aachen.de
Lisa.Fuertauer@bio3.rwth-aachen.de

Key words: *Salicornia europaea*, combined tolerance, hypoxia, salt, halophyte, stress response, RNA sequencing

Author contribution A.J., J.T.v.D. and L.F. designed the experiments, evaluated the data. A.J. and J.A. performed experiments. C.B. and P.N. performed RT-qPCR analyses. A.J.

computationally analyzed the data. A.J. and L.F. wrote the manuscript, J.T.v.D. revised the manuscript.

Conflicts of Interest The authors declare no conflict of interest.

Declaration of Funding AJ was partially, and CB fully funded by LFs WISNA Program.

Data Availability Statement The raw RNA-Seq data sets produced in this study can be accessed through NCBI under Bioproject ID PRJNA1256208 for shoot data and PRJNA1256210 for root data.

A Supplementary information

Table S1: **List of oligonucleotides used in this study**

Gene and GeneID	Primer Name	Sequence	T	Reference
Trehalose 6P phosphatase	SeTPPD1_F	CGCGAAAGTAGAGAACCAC	60	
TRINITY_DN8917_c0_g1	SeTPPD1_R	CTTAGGCCAAGGGATTCAAG	59	
Alcohol dehydrogenase	SeADH1_F	TTTGGGTGCAACTTTGAATG	59	
TRINITY_DN10866_c0_g1	SeADH1_R	TCTTGATGCACCACTGATTC	59	
Lactate dehydrogenase	SeLDH1_F	AGTCCCGGCTAAATCTTCTC	60	
TRINITY_DN1028_c0_g2	SeLDH1_R	CCGATAACCCGATTAGAAGG	59	
Pyrroline-5-carboxylate synthase	SeP5CS1_F	TATTGCTGCCTCTTGGAATG	59	
TRINITY_DN6160_c0_g1	SeP5CS1_R	ATCCTGCACAAGGTTATCAC	59	
Trehalase	SeTRE1_F	GAGAGCTTCTCCTGGTGTAG	60	
TRINITY_DN9548_c0_g1	SeTRE1_R	CGATTGCTGTAGCAGTCTTG	60	
Pyruvate decarboxylase	SePDC1_F	TCAGGACGTGTCAACCATGT	60	[16]
TRINITY_DN2949_c1_g1	SePDC1_R	TAAGGGCGGTGTAGTTCCAG	60.1	
Hemoglobin	SeAHB1_F	GACTCGGATGTTCCTTTGGA	60	[16]
TRINITY_DN2845_c0_g1	SeAHB1_R	TCCCACCGTAACTTTTCCAG	60	
Plant cysteine oxidase	SePCO1_F	GGCCGCCACTGTACCTACTA	60.2	[16]
TRINITY_DN2472_c0_g2	SePCO1_R	CGGGAGGCTTTTCTCTCTCT	60.1	
Na+/H+ Antiporter	SeNHX1_F	GGAGAATCGTTGGATGAATGAGTC	60	[44]
TRINITY_DN884_c0_g1	SeNHX1_R	GCTTCTTTTTTACCTGGAACCCTG	60	

Table S 2: **RNA Sequencing Results in .xlsx File Format.**

Sheet 1 $'Info'$: Project summary along with an overview of the sheets contained in the document.

Sheet 2+3 $'Shoot\_Data'$ and $'Root\_Data'$: Calculated log2FoldChanges, baseMeans, lfcSE, p-value, padj for each gene under various conditions, along with annotations and additive affiliations.

Sheet 4 $'Volcano\_highly\_regulated\_DEGS'$: Genes identified as highly differentially expressed in the volcano analysis.

Sheet 5 $'Venn\_Unique\_Genes'$: Genes uniquely associated with one of the stress conditions (H, HS, S) based on Venn analysis.

Sheet 6 $'Overlap\_highVolcano\_UniqueHS'$: Overlapping genes identified as highly expressed in both volcano plot analyses and uniquely expressed under simultaneous HS stress per Venn analysis.

Sheet 7 $'AddEff\_HRG\_SRG'$: Hypoxia- and salt-responsive genes utilized in additive effect analyses.

Sheet 8 $'Genes\_Carbohydrate\_Metabolism'$: All pathway analysis-related genes from carbohydrate metabolism.

Sheet 9 $'Genes\_AA\_Metabolism'$: All pathway analysis-related genes from amino acid metabolism.

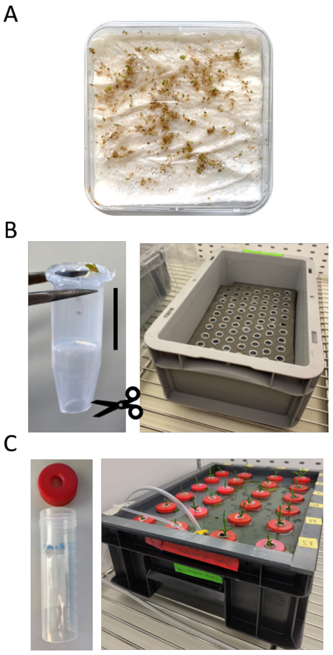

Figure S1: **Hydroponic cultivation of *Salicornia europaea.*** (**A**) Seeds of *Salicornia europaea* germinated on wet filter paper within a closed square plate for a duration of two weeks. (**B**) Seedlings were transferred to cultivate in liquid 1/2 Hoagland medium during a pre-culture phase lasting three weeks. The upper half (indicated by black bar) of each 1.5 ml reaction tube was filled with solid 1/2 Hoagland medium; tips and lids were removed for planting, allowing insertion into the solid medium for three weeks before further transfer to subsequent tubes. (**C**) Reaction tubes (50 ml) underwent preparation involving tip removal and side holes added to facilitate liquid 1/2 Hoagland medium circulation within the hydroponic system; each plant, along with its initial 1.5 ml reaction tube, was placed within a perforated lid atop these larger tubes. Plants were acclimated for one week before treatment application commenced at the six-week-old stage.

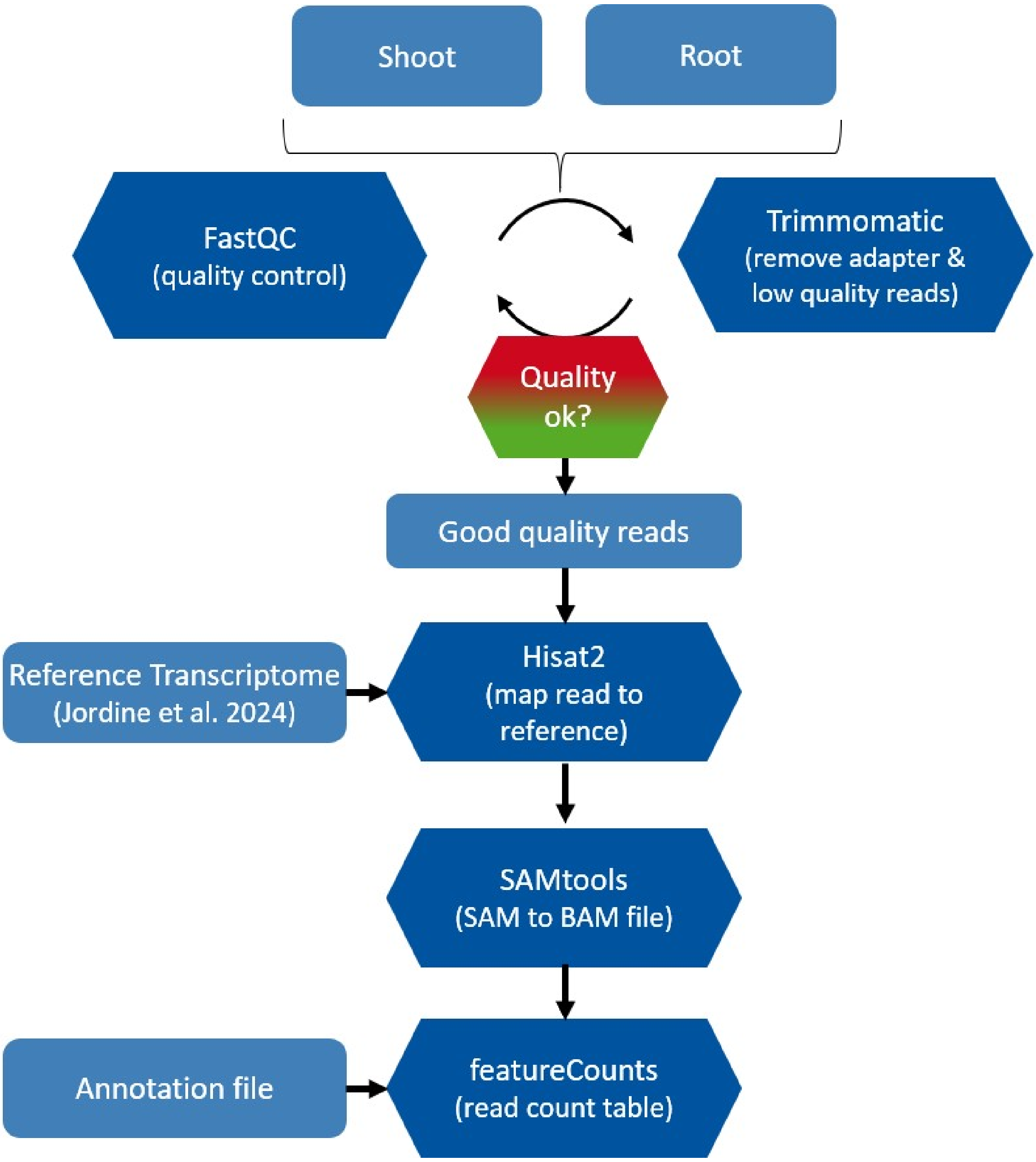

Figure S2: **Bioinformatics Workflow for the *in-silico* Processing of Raw Sequences in MiniConda.** Quality assessment of raw reads was conducted using FastQC, followed by cleaning to remove low-quality reads and adapter sequences via Trimmomatic. High-quality reads were aligned to a reference transcriptome [1] utilizing Hisat2. Aligned reads were converted from SAM to BAM files, after which a read count table was compiled with annotations using featureCounts.

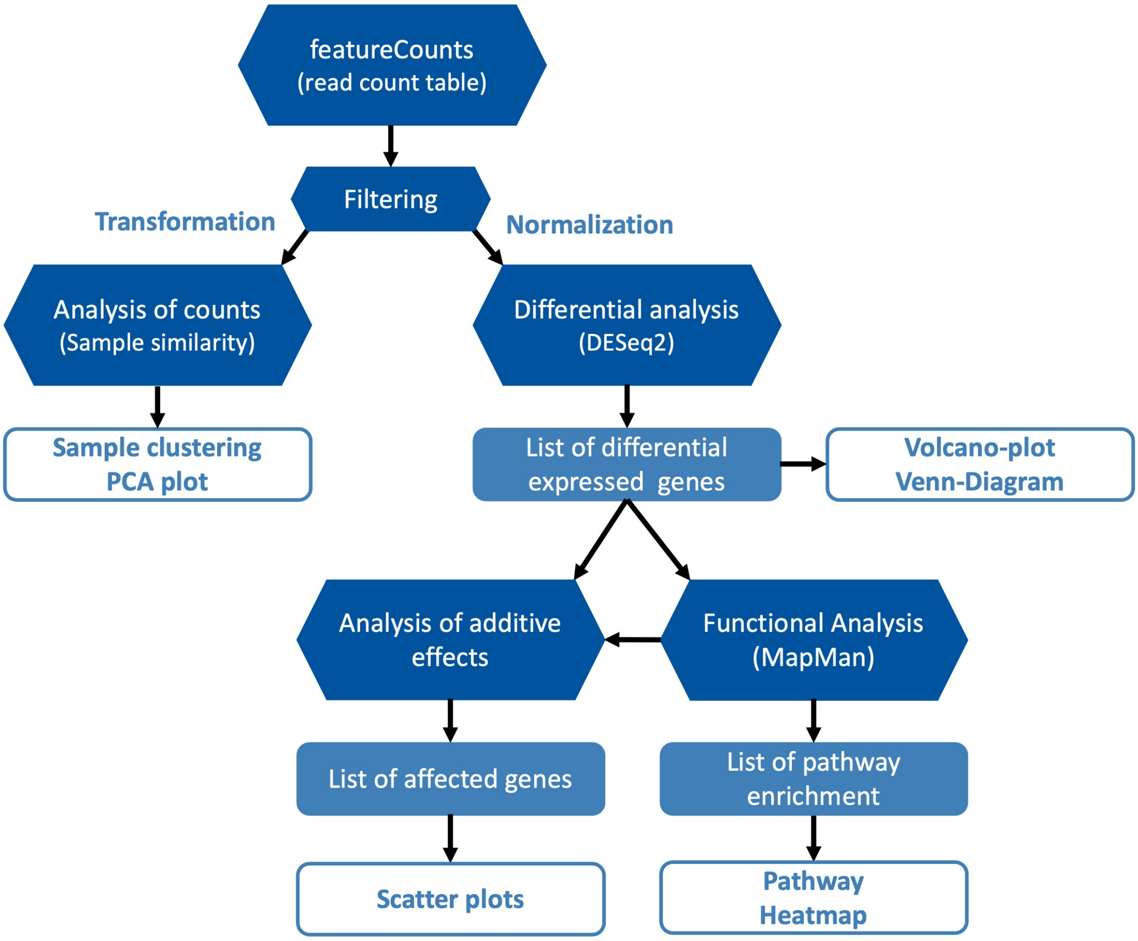

Figure S3: **Bioinformatics Workflow of the *in-silico* RNA Sequencing Analysis Conducted in R (https://www.r-project.org/).** The read count table was filtered to include counts present in at least three out of four replicate samples. Sample similarity analysis utilized rlog-transformed counts, which were subsequently applied to PCA analysis. Differential gene expression analysis involved normalizing counts using median of ratios normalization, followed by pairwise comparisons via DESeq2, yielding a list of differentially expressed genes (DEGs). Genes with an corrected p-value < 0.01 were classified as significant DEGs (sDEGs). The sDEG list underwent functional analysis using MapMan, producing a categorized gene list. Additive effect analysis was performed on annotated sDEGs within functional categories.

Abbreviations: rlog:= regularized-logarithm transformation, PCA:= Principle component analysis, DEGs:= differentially expressed genes

```
– Session info ──────────────────────────────────
setting  value
version  R version 4.3.3 (2024-02-29 ucrt)
os       Windows 10 x64 (build 19045)
system   x86_64, mingw32
ui       RStudio
language (EN)
collate  German_Germany.utf8
ctype    German_Germany.utf8
tz       Europe/Berlin
date     2024-08-05
rstudio  2024.04.1+748 Chocolate Cosmos (desktop)
pandoc   3.1.11 @ C:/Program Files/RStudio/resources/app/bin/quarto/bin/tools/ (via rmarkdown)

– Packages ──────────────────────────────────
package          * version  date (UTC) lib source
abind              1.4-5    2016-07-21 [1] CRAN (R 4.4.0)
Biobase          * 2.62.0   2023-10-24 [1] Bioconductor
BiocGenerics     * 0.48.1   2023-11-01 [1] Bioconductor
BiocParallel       1.36.0   2023-10-24 [1] Bioconductor
bitops             1.0-7    2021-04-24 [1] CRAN (R 4.3.1)
cli                3.6.2    2023-12-11 [1] CRAN (R 4.3.2)
codetools          0.2-19   2023-02-01 [2] CRAN (R 4.3.3)
colorspace         2.1-0    2023-01-23 [1] CRAN (R 4.3.2)
crayon             1.5.3    2024-06-20 [1] CRAN (R 4.3.3)
DelayedArray       0.28.0   2023-10-24 [1] Bioconductor
DESeq2           * 1.42.1   2024-03-06 [1] Bioconductor
3.18 (R 4.3.3)
digest             0.6.35   2024-03-11 [1] CRAN (R 4.3.3)
dplyr              1.1.4    2023-11-17 [1] CRAN (R 4.3.3)
evaluate           0.24.0   2024-06-10 [1] CRAN (R 4.3.3)
fansi              1.0.6    2023-12-08 [1] CRAN (R 4.3.2)
fastmap            1.1.1    2023-02-24 [1] CRAN (R 4.3.2)
generics           0.1.3    2022-07-05 [1] CRAN (R 4.3.2)
GenomeInfoDb     * 1.38.8   2024-03-15 [1] Bioconductor
3.18 (R 4.3.3)
GenomeInfoDbData   1.2.11   2024-02-02 [1] Bioconductor
GenomicRanges    * 1.54.1   2023-10-29 [1] Bioconductor
ggplot2            3.5.0    2024-02-23 [1] CRAN (R 4.3.3)
glue               1.6.2    2022-02-24 [1] CRAN (R 4.3.2)
gtable             0.3.5    2024-04-22 [1] CRAN (R 4.3.3)
htmltools          0.5.7    2023-11-03 [1] CRAN (R 4.3.2)
IRanges          * 2.36.0   2023-10-24 [1] Bioconductor
knitr              1.48     2024-07-07 [1] CRAN (R 4.3.3)
lattice            0.22-5   2023-10-24 [2] CRAN (R 4.3.3)
lifecycle          1.0.4    2023-11-07 [1] CRAN (R 4.3.2)
locfit             1.5-9.9  2024-03-01 [1] CRAN (R 4.3.3)
magrittr           2.0.3    2022-03-30 [1] CRAN (R 4.3.2)
Matrix             1.6-5    2024-01-11 [2] CRAN (R 4.3.3)
MatrixGenerics   * 1.14.0   2023-10-24 [1] Bioconductor
matrixStats      * 1.2.0    2023-12-11 [1] CRAN (R 4.3.2)
munsell            0.5.1    2024-04-01 [1] CRAN (R 4.3.3)
openxlsx           4.2.5.2  2023-02-06 [1] CRAN (R 4.3.2)
pillar             1.9.0    2023-03-22 [1] CRAN (R 4.3.2)
pkgconfig          2.0.3    2019-09-22 [1] CRAN (R 4.3.2)
R6                 2.5.1    2021-08-19 [1] CRAN (R 4.3.2)
Rcpp               1.0.12   2024-01-09 [1] CRAN (R 4.3.2)
RCurl              1.98-1.14 2024-01-09 [1] CRAN (R 4.3.2)
rlang              1.1.2    2023-11-04 [1] CRAN (R 4.3.2)
rmarkdown          2.27     2024-05-17 [1] CRAN (R 4.3.3)
rstudioapi         0.16.0   2024-03-24 [1] CRAN (R 4.3.3)
S4Arrays           1.2.1    2024-03-04 [1] Bioconductor 3.18 (R
4.3.2)
S4Vectors        * 0.40.2   2023-11-23 [1] Bioconductor
scales             1.3.0    2023-11-28 [1] CRAN (R 4.3.2)
sessioninfo      * 1.2.2    2021-12-06 [1] CRAN (R 4.3.3)
SparseArray        1.2.4    2024-02-11 [1] Bioconductor 3.18
(R 4.3.2)
stringi            1.8.3    2023-12-11 [1] CRAN (R 4.3.2)
SummarizedExperiment * 1.32.0   2023-10-24 [1]
Bioconductor
tibble             3.2.1    2023-03-20 [1] CRAN (R 4.3.2)
tidyselect         1.2.1    2024-03-11 [1] CRAN (R 4.3.3)
utf8               1.2.4    2023-10-22 [1] CRAN (R 4.3.2)
vctrs              0.6.5    2023-12-01 [1] CRAN (R 4.3.2)
xfun               0.46     2024-07-18 [1] CRAN (R 4.3.3)
XVector            0.42.0   2023-10-24 [1] Bioconductor
yaml               2.3.8    2023-12-11 [1] CRAN (R 4.3.2)
zip                2.3.1    2024-01-27 [1] CRAN (R 4.3.2)
zlibbioc           1.48.0   2023-10-24 [1] Bioconductor

[1] C:/Users/WHJ/AppData/Local/R/win-library/4.3
[2] C:/Program Files/R/R-4.3.3/library
```

Figure S4: **Session Information from RStudio.** This session information from RStudio [2] details the computational environment utilized for data processing and analysis. The output provides specifics on the R version, operating system, and all loaded packages along with their respective versions.

A	Control	Salt	Hypoxia	Hypoxia Salt
S1	32733971	31488305	32075179	32650088
S2	34994880	25255162	28738079	36029164
S3	28931538	37293006	32199868	10971817
S4	29962147	34475604	32373280	34493738
R1	21115587	23482095	15508626	19984821
R2	22461467	26832376	18964709	21478318
R3	25659988	24771396	16512004	24284604
R4	23806589	24386876	17791671	20912246

B	Control	Salt	Hypoxia	Hypoxia Salt
S1	68.00%	68.56%	65.80%	67.17%
S2	63.81%	68.25%	65.84%	68.21%
S3	69.18%	66.69%	67.73%	67.93%
S4	67.72%	69.80%	65.82%	66.31%
R1	58.63%	59.40%	59.09%	54.78%
R2	61.45%	56.55%	52.80%	59.46%
R3	62.42%	63.53%	59.92%	62.24%
R4	63.09%	61.05%	57.85%	62.01%

Figure S5: **Alignment Information from Hisat2.** Hisat2 [3] facilitated the alignment of RNAseq reads to the reference transcriptome [1]. (**A**) Displays the number of reads per sample, and (**B**) shows overall alignment rates for shoot (S1-S4) and root (R1-R4) samples across different conditions (control: **C**; salt: **S**; hypoxia: **H**, hypoxia-salt: **HS**).
Abbreviations: S:= shoot, R:= root, 1-4:= replicate number

	Shoot			Root		
**A**	Salt	Hypoxia-Salt	Hypoxia	Salt	Hypoxia-Salt	Hypoxia
Total	13086	13086	13086	13220	13220	13220
p-val<0.01	4670	5833	2844	3701	6568	5264
p-val>0.01	8416	7253	10242	9519	6652	7956

	Shoot			Root		
**B**	Salt	Hypoxia-Salt	Hypoxia	Salt	Hypoxia-Salt	Hypoxia
Total	9405	9405	9405	9524	9524	9524
p-val<0.01	3712<br>39%	4551<br>48%	2199<br>23%	2903<br>30%	5137<br>53%	4117<br>43%
p-val>0.01	5693	4853	7206	6621	4387	5407
Sig/no-sig	0.65	0.93	0.30	0.44	1.17	0.76
log2FC>0	1819	2202	1130	1416	2612	2001
log2FC<0	1893	4551	2199	1487	2525	2116

Figure S6: **Figures from Differentially Gene Expression Analysis.** (**A**) Displays the number of differentially expressed genes, and (**B**) shows differentially expressed annotated genes. 'Total' represents the complete count of differentially expressed genes. The row p-val$<$0.01 indicates all significant differentially expressed genes (sDEGs) in both numbers and percentages, whereas p-val $>$ 0.01 denotes non-significant DEGs. Log2FC $>$ 0 captures up-regulated sDEGs, while Log2FC $<$ 0 encompasses down-regulated sDEGs.

Abbreviations: sDEGs:= significant differentially expressed genes, Log2FC:= logarithmic fold change, p-val:= significance value after correction, sig:= significant, no-sig:= not significant

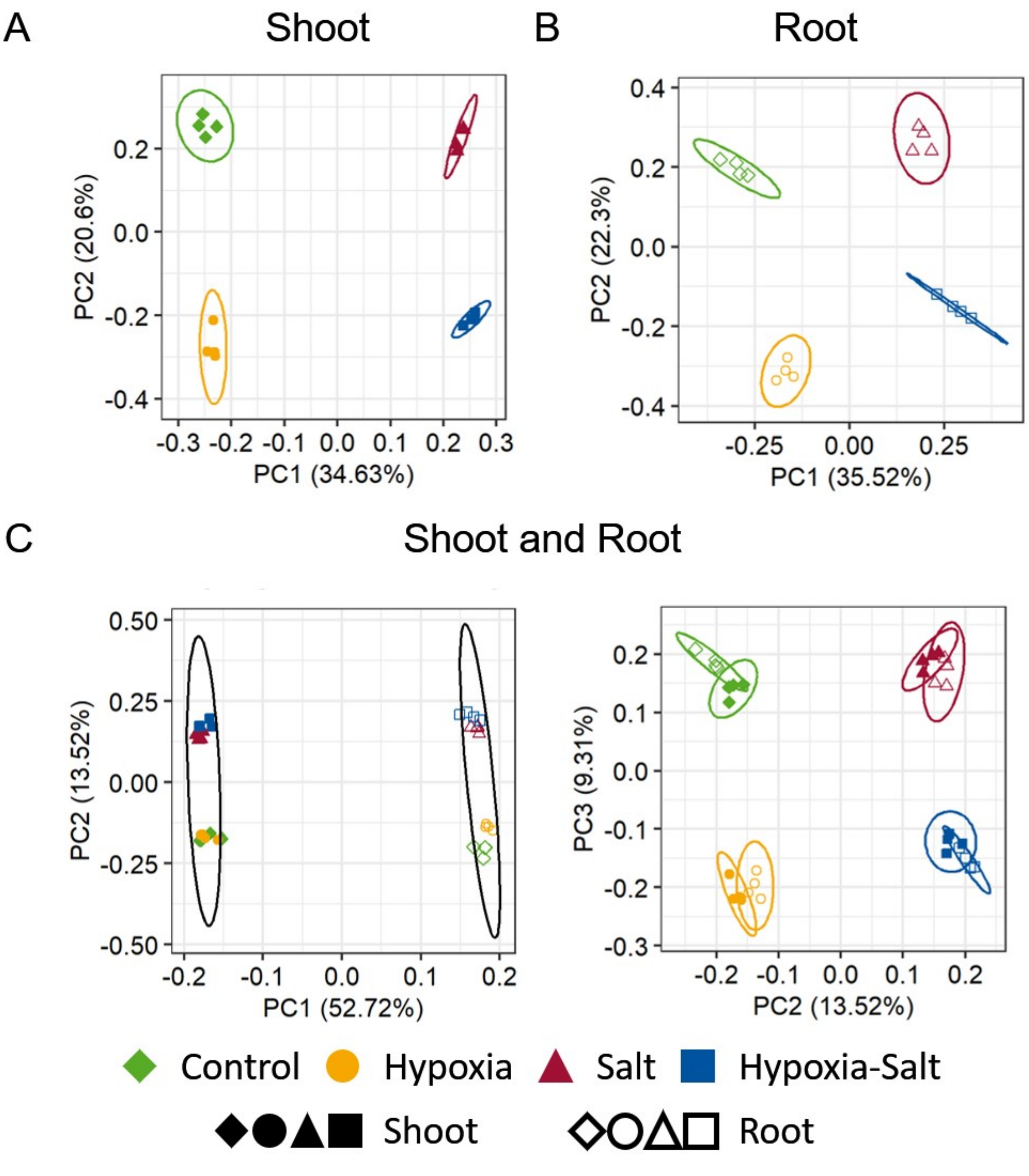

Figure S7: **Principle Component Analysis (PCA) of All Genes from RNA Sequencing Datasets.** Counts underwent regularized log (rlog) transformation before being analyzed separately for (**A**) shoot samples, (**B**) root samples, and (**C**) the combined dataset. Different experimental conditions are depicted by distinct symbols and colors. filled shapes: shoot samples; empty shapes: root samples; green-diamond: control, yellow-cycle: hypoxia; red-rectangle: salt; blue-square: hypoxia salt,

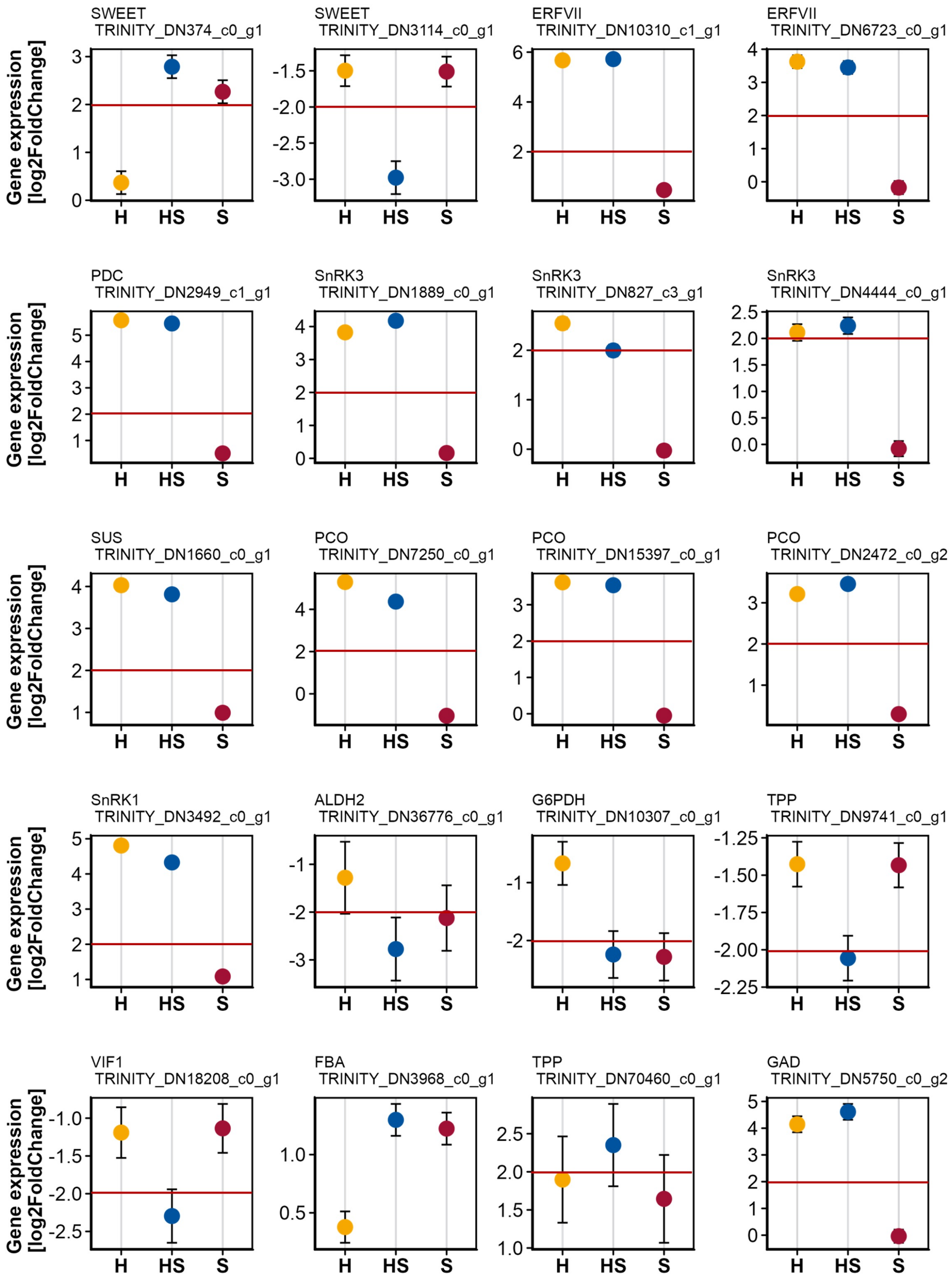

Figure S8: **Shoot-Specific Differential Gene Expression of Selected Highly sDEGs.** Example genes identified as highly differentially expressed in shoots and/or roots under hypoxia-salt (**HS**) stress (log2FoldChange). Red line marks the log2FC threshold of log2FC>2 and log2FC<-2. Gene names along with their corresponding TrinityIDs are provided above each graph. **H**: Hypoxia (yellow); **HS**: Hypoxia-salt (blue); **S**: Salt (red)

Abbreviations: SWEET:= Sugar will eventually be exported transporter; SUS:= Sucrose synthase; PCO:= Plant cysteine oxidase; ERFVII:= Ethylene responsive factors group VII; PDC:= Pyruvate decarboxylase; ALDH2:= Aldehyde dehydrogenase; TPP:= Trehalose-6-phosphate phosphatase; VIF2:= Vacuolar/cell wall invertase inhibitor; FBA1:= Fructose-1,6-bisphosphate aldolase 1; G6PDH:= Glucose-6-phosphate dehydrogenase; GAD:= Glutamate decarboxylase; SnRK:= Sucrose non-fermenting 1 related protein kinase

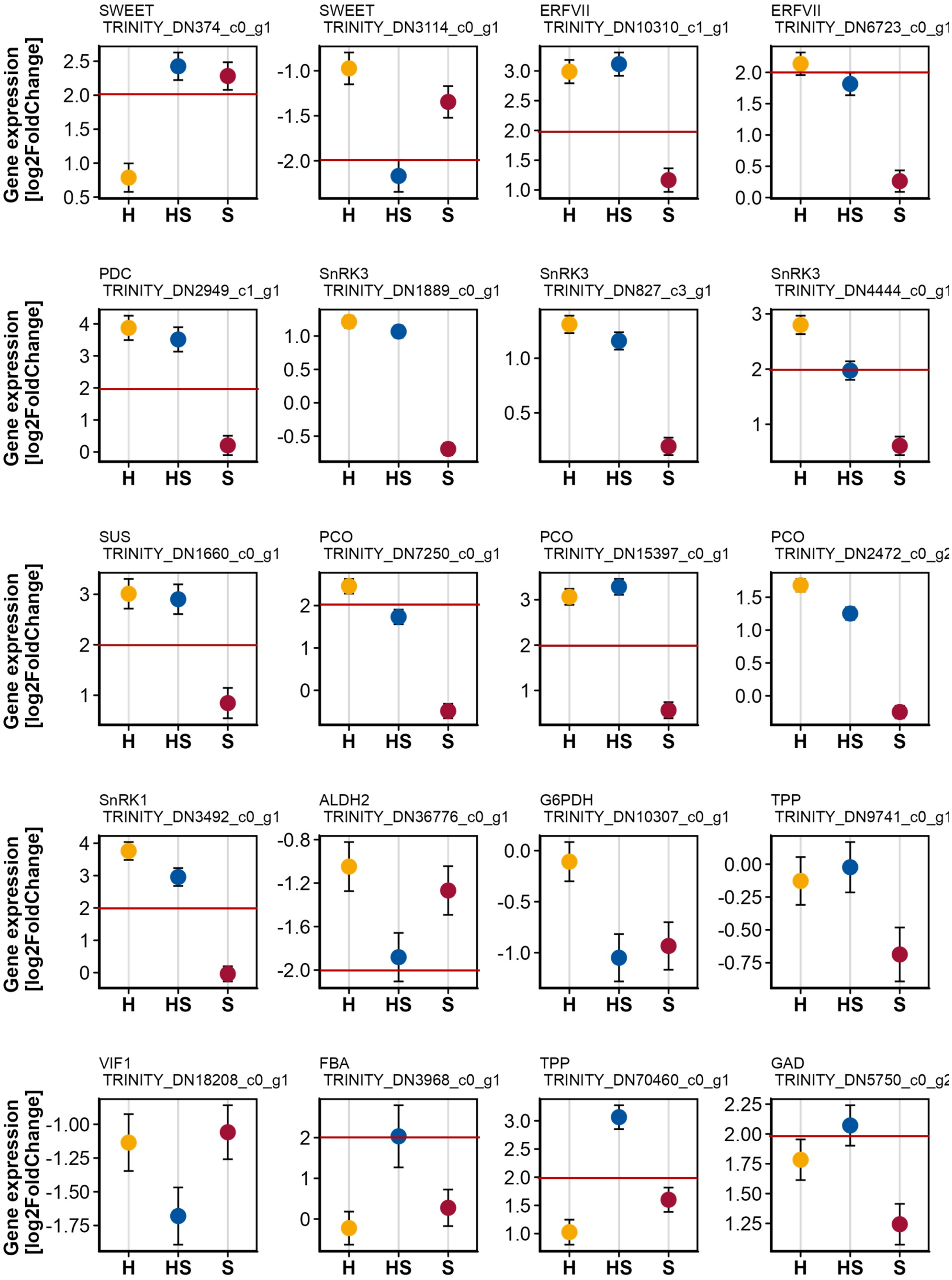

Figure S9: **Root-Specific Differential Gene Expression of Selected Highly sDEGs.** Example genes identified as highly differentially expressed in shoots and/or roots under hypoxia-salt (**HS**) stress (log2FoldChange). Red line marks the log2FC threshold of log2FC>2 and log2FC<-2. Gene names along with their corresponding TrinityIDs are provided above each graph. **C**: Control (green); **H**: Hypoxia (yellow); **HS**: Hypoxia-salt (blue); **S**: Salt (red)

Abbreviations: SWEET:= Sugar will eventually be exported transporter; SUS:= Sucrose synthase; PCO:= Plant cysteine oxidase; ERFVII:= Ethylene responsive factors group VII; PDC:= Pyruvate decarboxylase; ALDH2:= Aldehyde dehydrogenase; TPP:= Trehalose-6-phosphate phosphatase; VIF2:= Vacuolar/cell wall invertase inhibitor; FBA1:= Fructose-1,6-bisphosphate aldolase 1; G6PDH:= Glucose-6-phosphate dehydrogenase; GAD:= Glutamate decarboxylase; SnRK:= Sucrose non-fermenting 1 related protein kinase

BIN	Main categories	Total	Shoot S	Shoot HS	Shoot H	Root S	Root HS	Root H
4	Amino acid metabolism	175	0.791	0.840	0.791	0.846	0.813	0.564
3	Carbohydrate metabolism	160	0.886	0.958	0.886	0.849	0.865	0.835
12	Cell division	310	0.839	0.342	0.839	0.869	0.188	0.000
21	Cell wall organisation	294	0.815	0.947	0.815	0.000	0.000	0.835
2	Cellular respiration	130	0.094	0.052	0.094	0.874	0.900	0.922
11	Chromatin organisation	285	0.779	0.858	0.779	0.883	0.917	0.835
30	Clade-specific metabolism	6	0.850	0.990	0.850	0.846	0.865	0.945
7	Coenzyme metabolism	208	0.000	0.000	0.000	0.846	0.865	0.839
22	Cytoskeleton organisation	190	0.779	0.940	0.779	0.846	0.850	0.008
13	DNA damage response	78	0.779	0.900	0.779	0.514	0.883	0.899
26	External stimuli response	200	0.601	0.960	0.601	0.932	0.865	0.532
5	Lipid metabolism	305	0.964	0.939	0.964	0.846	0.866	0.875
27	Multi-process regulation	343	0.004	0.840	0.004	0.374	0.867	0.652
6	Nucleotide metabolism	89	0.779	0.840	0.779	0.969	0.917	0.962
25	Nutrient uptake	132	0.839	0.906	0.839	0.973	0.966	0.975
1	Photosynthesis	224	0.000	0.000	0.000	0.000	0.000	0.835
10	Phytohormone action	263	0.837	0.840	0.837	0.849	0.867	0.835
29	Plant organogenesis	104	0.526	0.853	0.526	0.966	0.878	0.962
28	Plant reproduction	40	0.929	0.943	0.929	0.954	0.981	0.994
17	Protein biosynthesis	499	0.030	0.000	0.030	0.846	0.887	0.003
20	Protein homeostasis	636	0.717	0.840	0.717	0.264	0.411	0.072
18	Protein modification	580	0.029	0.840	0.029	1.000	0.865	0.006
19	Protein physical control	316	0.779	0.495	0.779	0.846	0.412	0.010
9	Redox homeostasis	138	0.178	0.143	0.178	0.968	0.998	0.835
14	RNA biosynthesis	768	0.289	0.940	0.289	0.340	0.865	0.835
16	RNA homeostasis	189	0.985	0.840	0.985	0.846	0.964	0.835
15	RNA processing	346	0.563	0.180	0.563	0.927	0.908	0.857
8	Secondary metabolism	84	0.007	0.345	0.007	0.089	0.936	0.001
24	Solute transport	591	0.869	0.840	0.869	0.984	0.865	0.835
23	Vesicle trafficking	383	0.000	0.000	0.000	0.000	0.000	0.839

Figure S10: **Probability of Enriched Categories in Shoots and Roots.** Results of the Wilcoxon-Mann-Whitney test were extracted from MapMan's statistical tab. Displayed here are BINs, main categories, total element counts within each BIN, and their corresponding probabilities for each category under various conditions in shoot and root data. Enrichment probabilities were calculated according to Wilcoxon-Mann-Whitney; low probabilities are color-coded blue while high probabilities are red. **H**: Hypoxia (yellow); **HS**: Hypoxia-salt (blue); **S**: Salt (red)

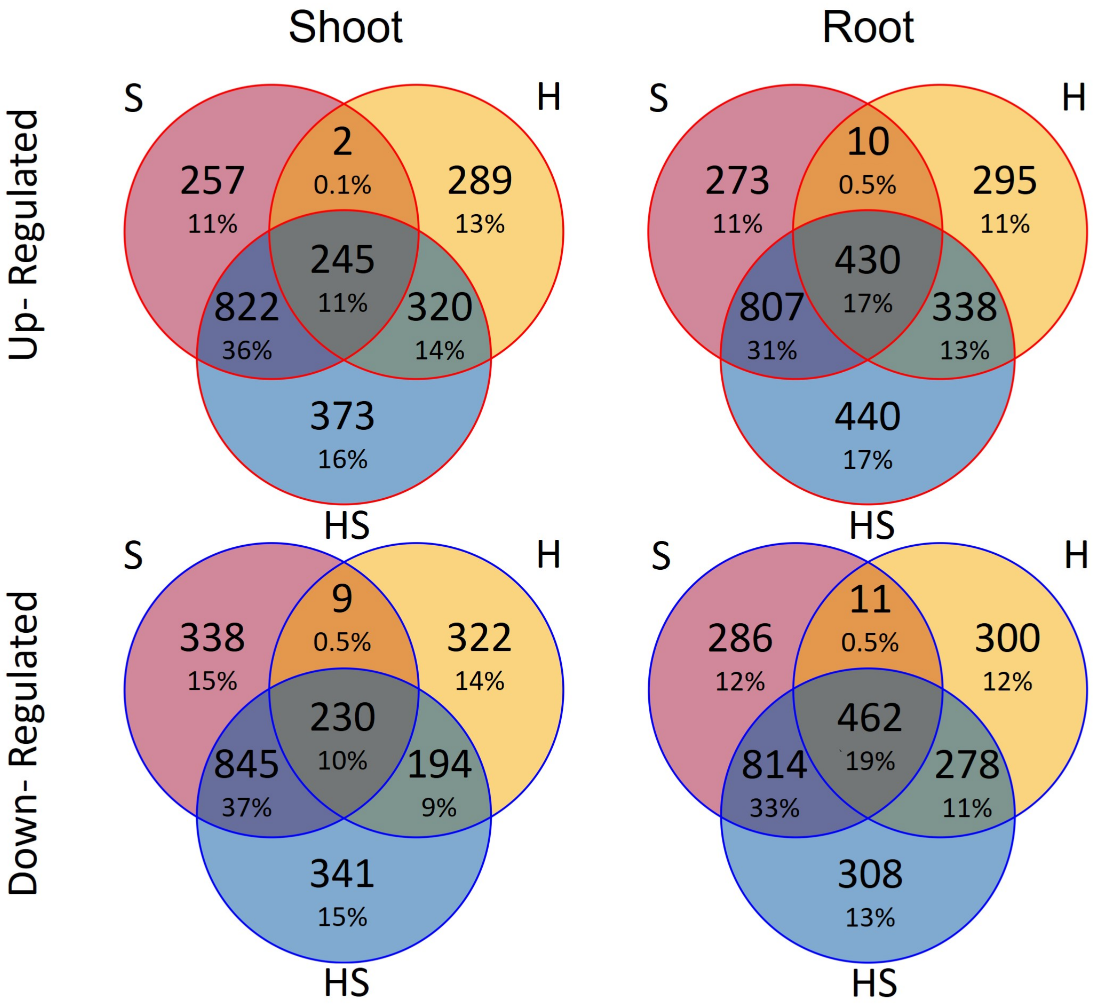

Figure S11: **Specificity of the Up- and Down-Regulated sDEGs for the Given Conditions and Their Overlaps.** The overlap of sDEGs (significant differentially expressed genes, p-value <0.01) across individual hypoxia, salt, and simultaneous hypoxia-salt stress for up-regulated (red border) and down-regulated (blue border) sDEGs. Each circle represents the sDEGs set for one condition, with overlapping areas indicating shared genes between conditions, while unique genes are shown in non-overlapping sections. **H**: Hypoxia (yellow); **HS**: Hypoxia-salt (blue); **S**: Salt (red)

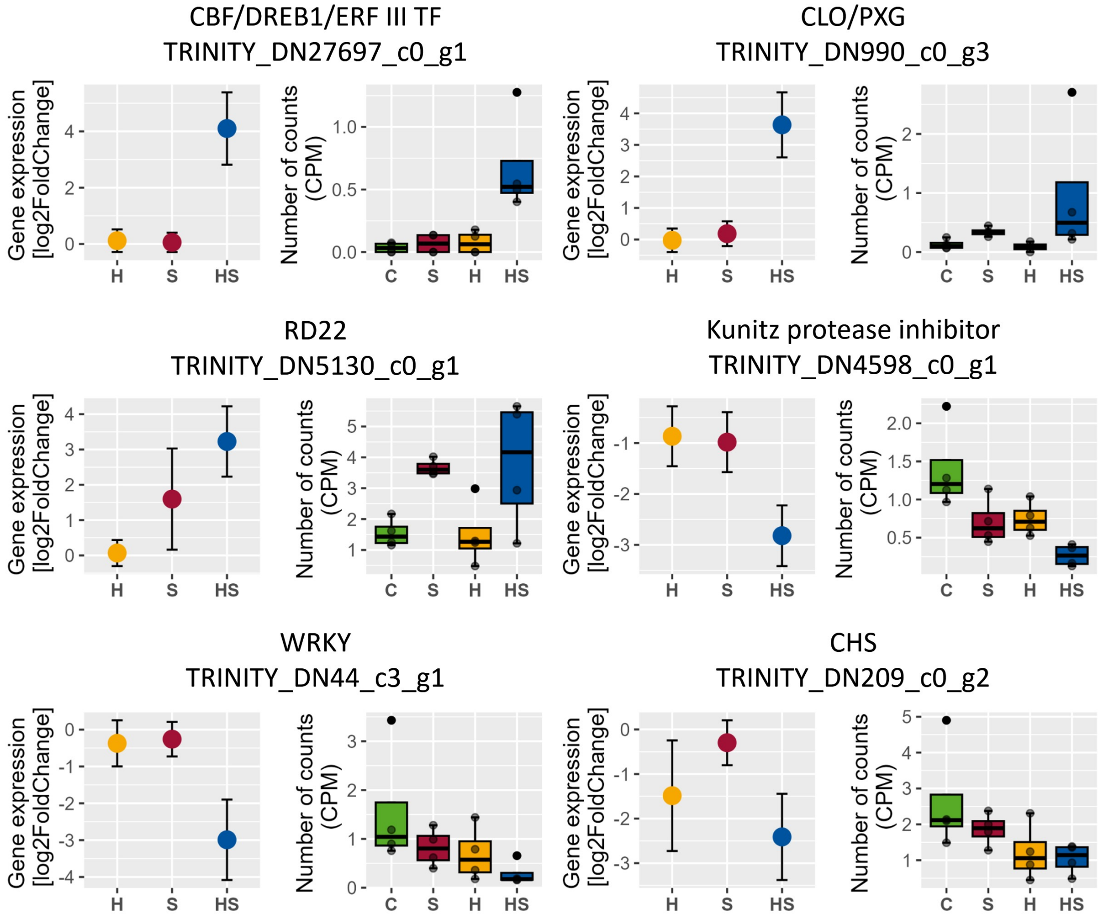

Figure S12: **Examples of Unique, Highly Differentially Expressed enhanced or divergent Hypoxia-Salt Genes of Shoots.** Example genes identified as unique, highly differentially expressed, enhanced or divergent under hypoxia-salt (**HS**) in shoots. Unique **HS** genes from Venn analysis were filtered for highly gene expression (log2FoldChange>2 or log2FoldChange<-2) and enhanced and divergent characteristics. For each gene, differential gene expression value (log2FoldChange) and standard error were extracted from the DESeq analysis and displayed as dotplot with the respective standard error. The number of counts was extracted from the raw read count table. The four replicate values were displayed for each condition in a boxplot. **C**: Control (green); **H**: Hypoxia (yellow); **HS**: Hypoxia-salt (blue); **S**: Salt (red)

Abbreviations: CBF/DREB1/ERFIII TF:= Ethylene responsive factors III transcription factor; CLO/PXG:= Caleosin-type peroxygenase; PIP5K:= Phosphatidylinositol 4-phosphate 5-kinase; WRKY:= WRKY amino acid sequence; CHS:= Chalcone synthase

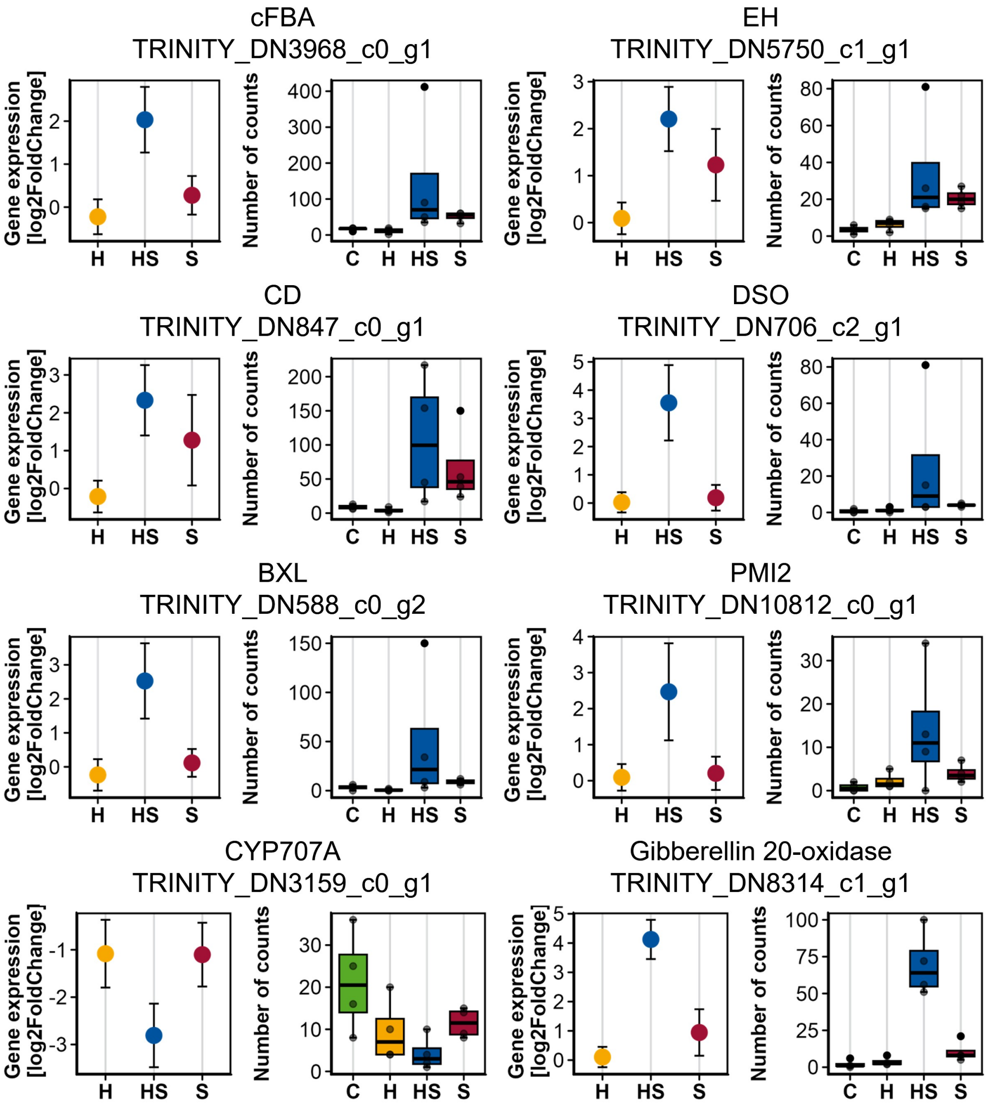

Figure S13: **Selection of Unique, Highly Differentially Expressed enhanced or divergent Hypoxia-Salt Genes of Roots.** Example genes identified as unique, highly differentially expressed, enhanced or divergent under hypoxia-salt (**HS**) in roots. Unique **HS** genes from Venn analysis were filtered for high gene expression (log2FoldChange>2 or log2FoldChange<-2) and enhanced and divergent characteristics. For each gene, differential gene expression value (log2FoldChange) and standard error were extracted from the DESeq analysis and displayed as dotplot with the respective standard error. The number of counts was extracted from the raw read count table. The four replicate values were displayed for each condition in a boxplot. Differential expression of photosynthesis genes can be explained by either contamination with algae in the hydroponic system, or unusual alteration of gene expression in the roots by light irradiance at the upper sections of the roots due to the cultivation setup. **C**: Control (green); **H**: Hypoxia (yellow); **HS**: Hypoxia-salt (blue); **S**: Salt (red)

Abbreviations: FBA:= Fructose-bisphosphate aldolase; EH:= Epoxide hydrolase; CD:= Cutin synthase; DSO:= Suberin/cutin lipid exporter; BXL:= Bifunctional alpha-L-arabinofuranosidase and beta-D-xylosidase; PMI:= WEB1-PMI2 actin filament reorganisation complex; CYP707A:= Abscisic acid 8'-hydroxylase 1; FKBP:= FKBP prolyl isomerase; ABCG:= ATP-binding cassette transporter

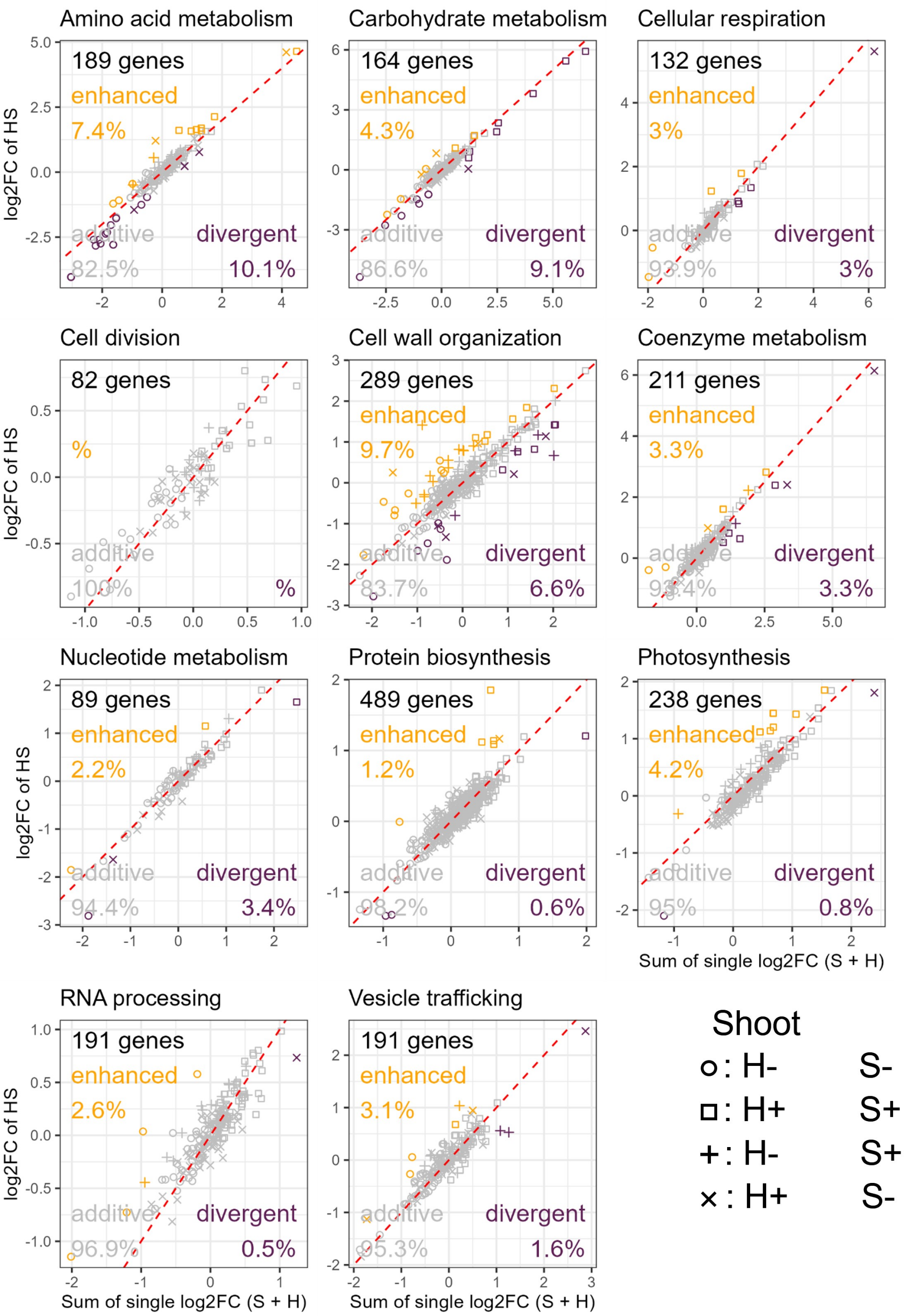

Figure S14: **Deviation of Significant Gene Expression Responses from Additive Effects Under Combined Hypoxia-Salt Stress.** The relationship between the summed log2FoldChanges (log2FC) of individual stress responses (salt and hypoxia) and the log2FC under simultaneous hypoxia-salt (**HS**) stress in shoots for all enriched functional categories (Suppl. Fig. S10, red). Grey markers denote genes with additive effects, while orange and violet markers indicate genes with enhanced or divergent effects, respectively. Additive effects were defined as follows: when the sum of individual stress responses matches the **HS** response (FC(**HS**) = FC(**H**+**S**) ±FC(0.5) confidence interval). enhanced effects were defined when **HS** expression levels exceed the sum by at least 0.5 FC, and divergent effects, when **HS** was below the sum by 0.5 FC threshold. The red diagonal denote the expected trend for additive responses. Symbols represent the sign of log2FC of the individual stress (circle: both (**H** and **S**) negative; square: both (**H** and **S**) positive; +: **H** negative and **S** positive; ×: **H** positive and **S** negative)
Abbreviations: FC:= fold change

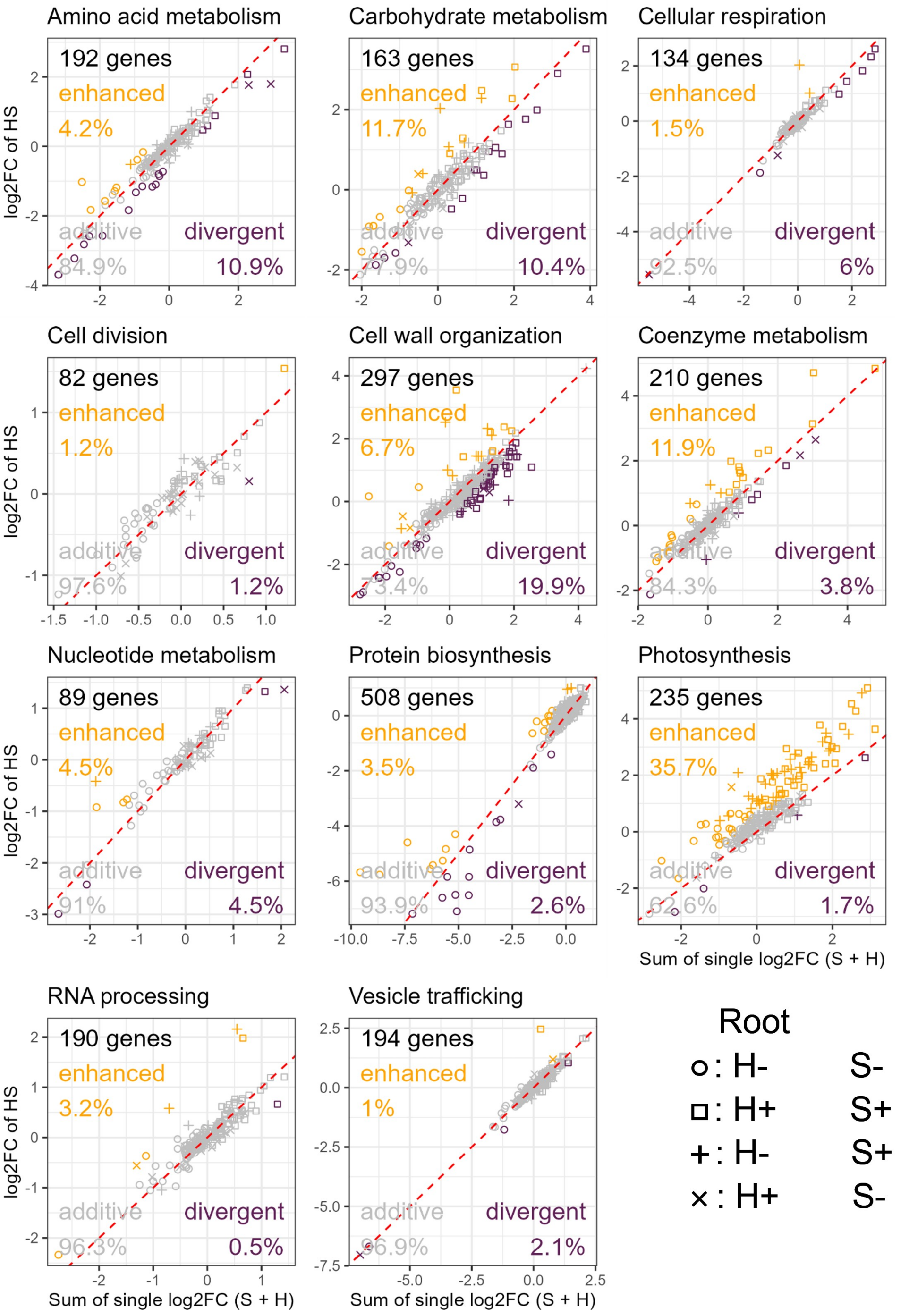

Figure S15: **Deviation of Significant Gene Expression Responses from Additive Effects Under Combined Hypoxia-Salt Stress** The relationship between the summed log2FoldChanges (log2FC) of individual stress responses (salt and hypoxia) and the log2FC under simultaneous hypoxia-salt (**HS**) stress in roots for all enriched functional categories (Suppl. Fig. S10, red). Grey markers denote genes with additive effects, while orange and violet markers indicate genes with enhanced or divergent effects, respectively. Additive effects were defined as follows: when the sum of individual stress responses matches the **HS** response (FC(**HS**) = FC(**H**+**S**) ±FC(0.5) confidence interval). enhanced effects were defined when **HS** expression levels exceed the sum by at least 0.5 FC, and divergent effects, when **HS** was below the sum by 0.5 FC threshold. The red diagonal denote the expected trend for additive responses. Symbols represent the sign of log2FC of the individual stress (circle: both (**H** and **S**) negative; square: both (**H** and **S**) positive; +: **H** negative and **S** positive; ×: **H** positive and **S** negative).

Abbreviations: FC:= fold change

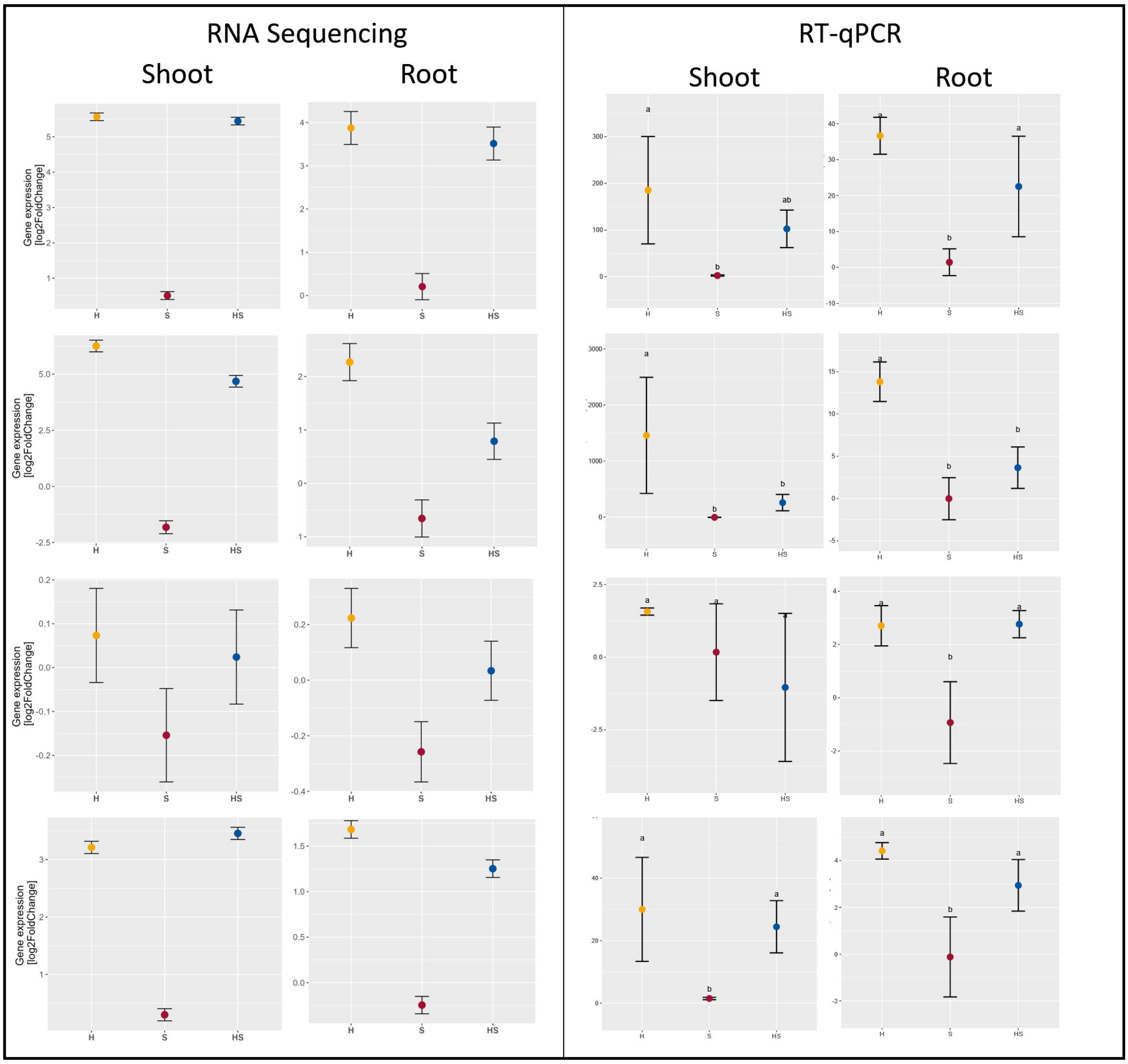

Figure S16: **Validation of RNA sequencing pattern *via* RT-qPCR** RNA sequencing pattern of hypoxia and salt responsive genes were validated using RT-qPCR. For shoots and roots log2 fold expression changes were displayed in dotplots with their respective standard error. For both analyses, the same plant material was used. Different letters above the dotplots indicate a significant difference according to one-way ANOVA followed by Tukey's *post hoc* test. **H**: Hypoxia (yellow); **S**: Salt (red); **HS**: Hypoxia-Salt (blue).

Abbreviations: PDC:= Pyruvate decarboxylase; AHB := Hemoglobin; NHX:= Na+/H+ Antiporter; PCO:= Plant cysteine oxidase

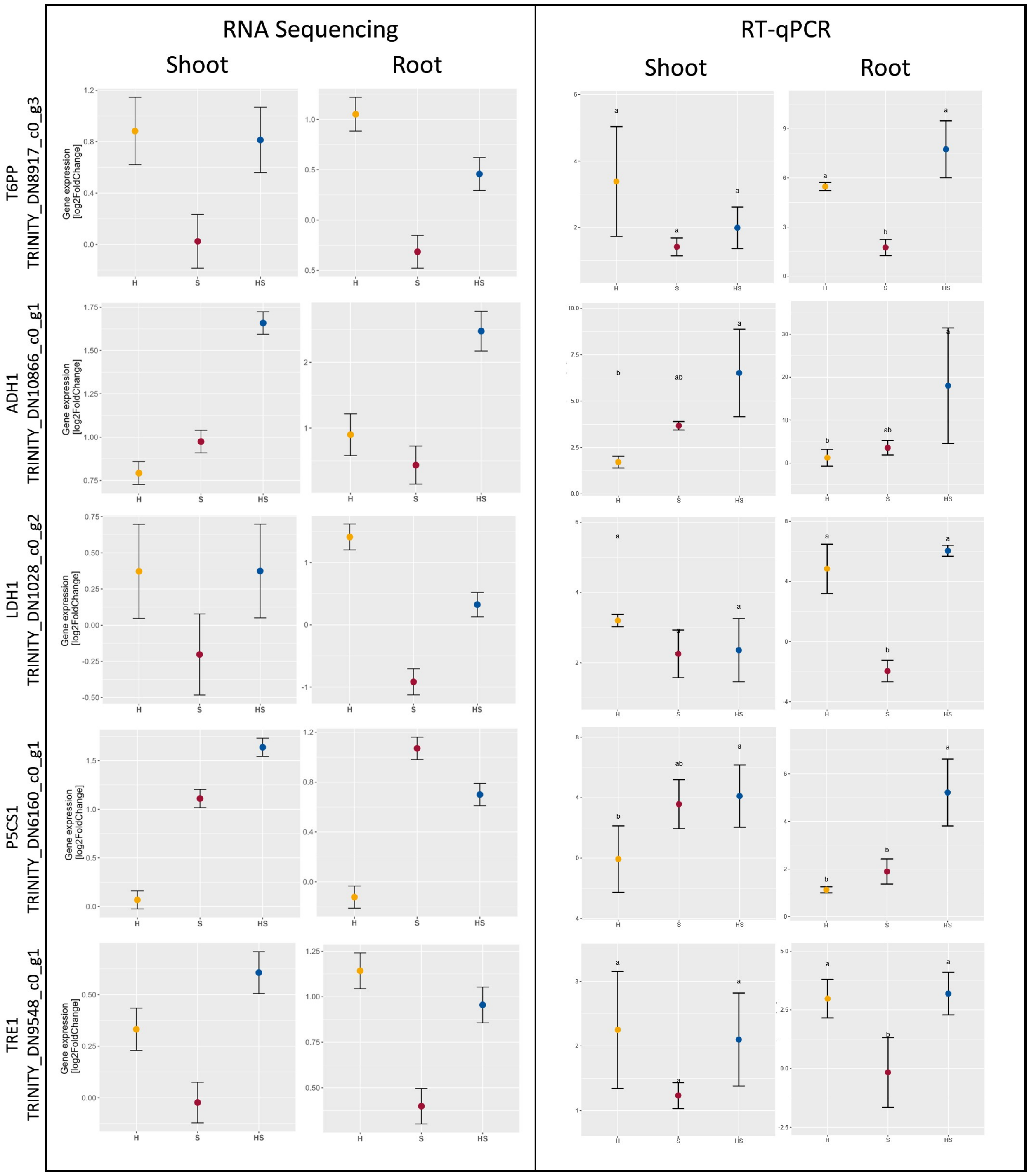

Figure S17: **Validation of RNA sequencing pattern *via* RT-qPCR** RNA sequencing pattern of hypoxia and salt responsive genes were validated using RT-qPCR. For shoots and roots log2 fold expression changes were displayed in dotplots with their respective standard error. For both analyses, the same plant material was used. Different letters above the dotplots indicate a significant difference according to one-way ANOVA followed by Tukey's *post hoc* test. **H**: Hypoxia (yellow); **S**: Salt (red); **HS**: Hypoxia-Salt (blue).

Abbreviations: T6PP:= Trehalose 6P phosphatase; ADH := Alcohol dehydrogenase; LDH:= Lactate dehydrogenase; P5CS:= Pyrroline-5-carboxylate synthase; TRE:= Trehalase
