# Supplementary figures and images for: Insights into enhanced, divergent, and additive responses to single and combined hypoxia-salt stress
Source: BMC Plant Biol. 2026 Mar 27;26:642. doi: 10.1186/s12870-026-08595-7 (PMC13063762; doi:10.1186/s12870-026-08595-7)

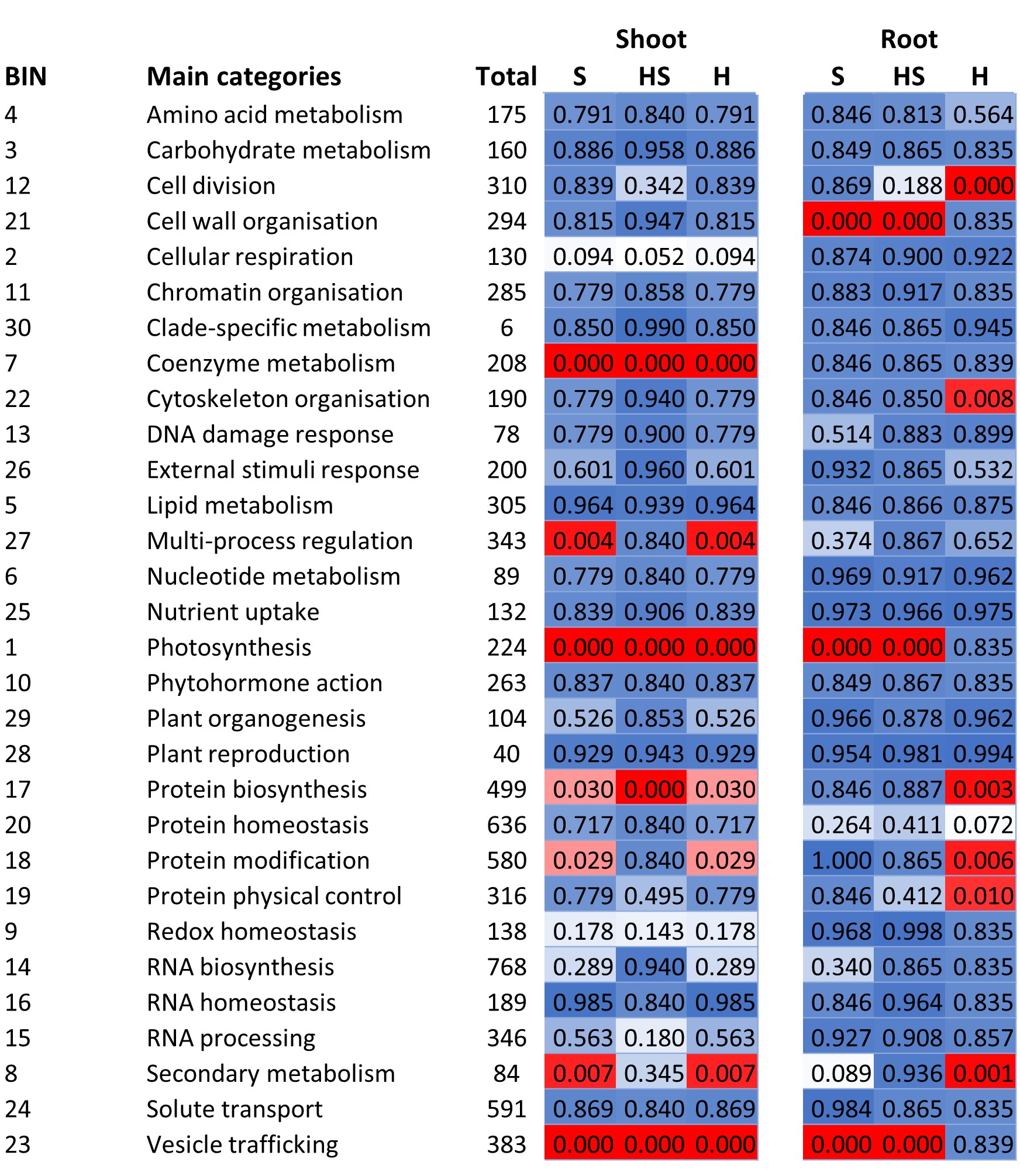

Supplement: Supplementary file 1 — Supplementary Material 1. [file 12870_2026_8595_MOESM1_ESM.zip › Supplementary Material/Supplement/Fig_S10_Mapman_Cat_Map.jpg]

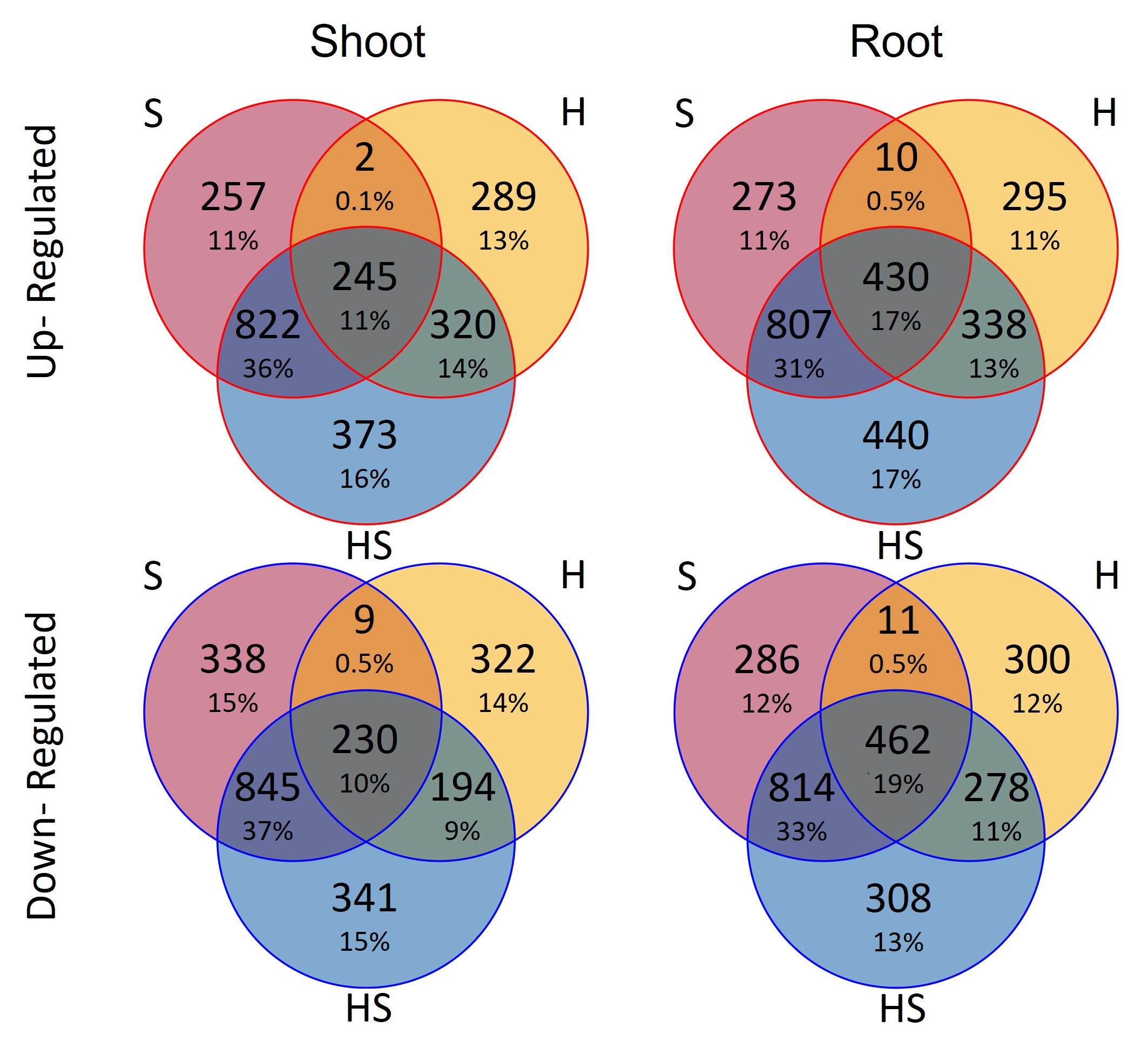

Supplement: Supplementary file 1 — Supplementary Material 1. [file 12870_2026_8595_MOESM1_ESM.zip › Supplementary Material/Supplement/Fig_S11_Venn_UpandDown.jpg]

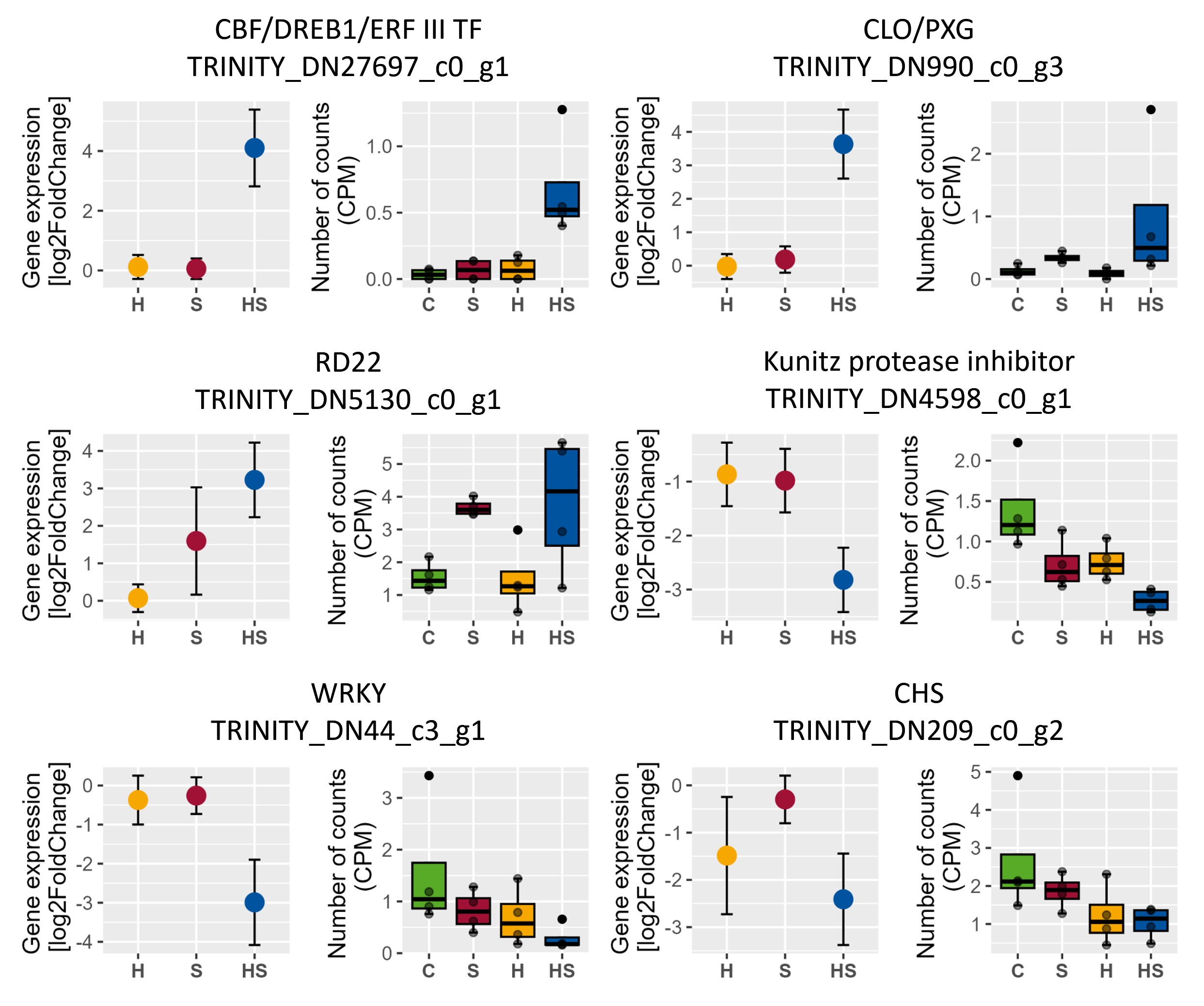

Supplement: Supplementary file 1 — Supplementary Material 1. [file 12870_2026_8595_MOESM1_ESM.zip › Supplementary Material/Supplement/Fig_S12_VolVennOverlap_Shoot.jpg]

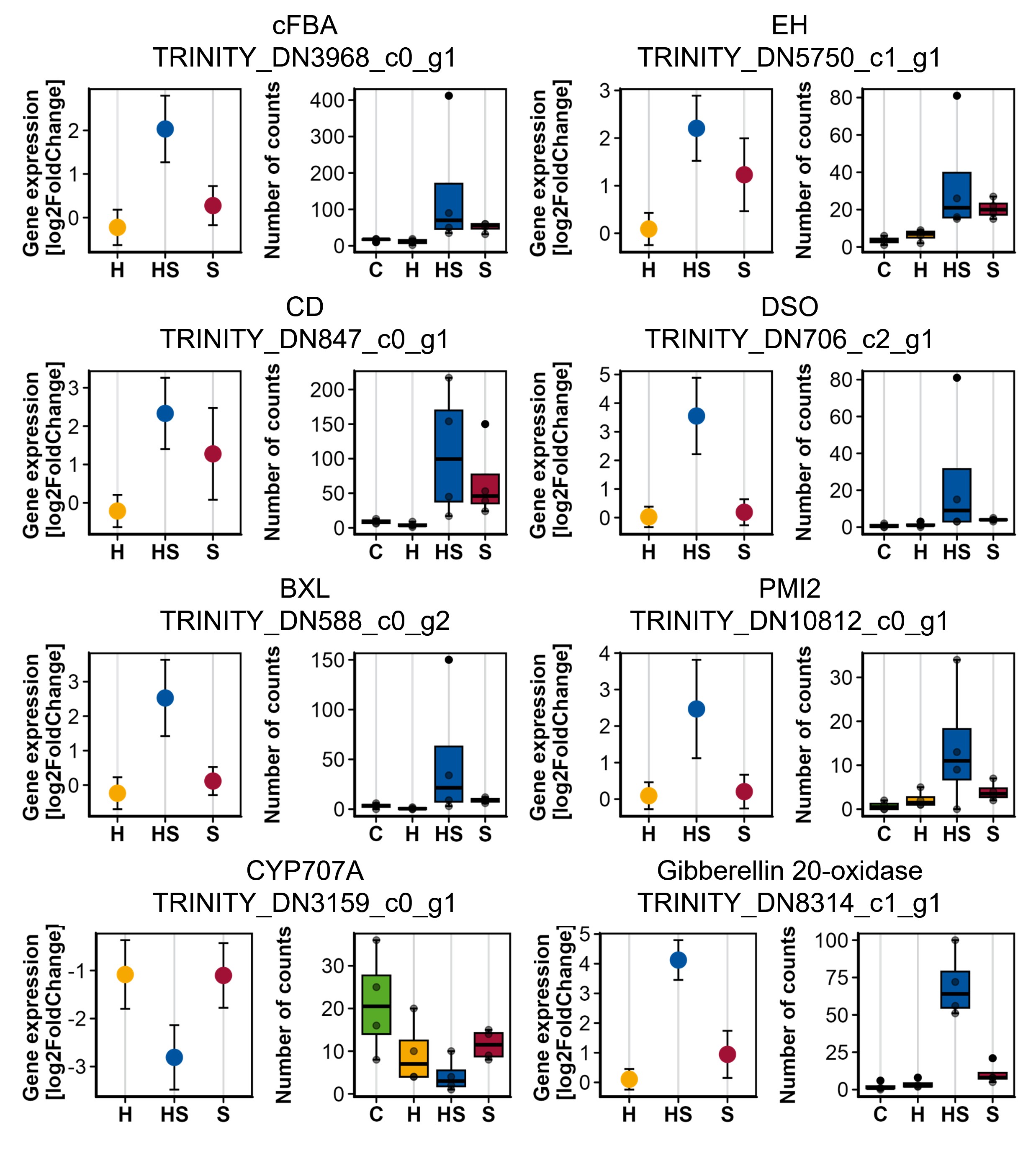

Supplement: Supplementary file 1 — Supplementary Material 1. [file 12870_2026_8595_MOESM1_ESM.zip › Supplementary Material/Supplement/Fig_S13_VolVennOverlap_Root.jpg]

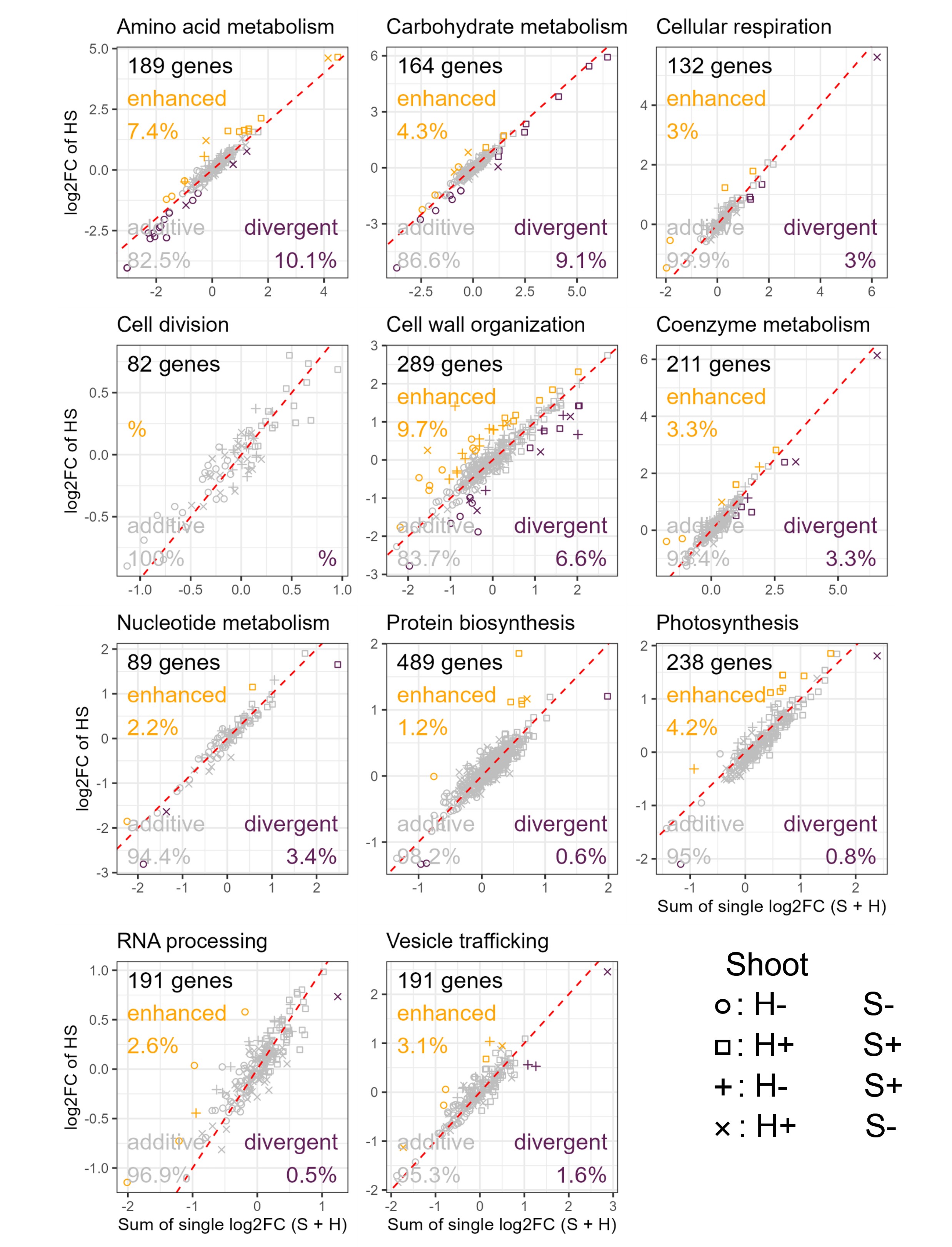

Supplement: Supplementary file 1 — Supplementary Material 1. [file 12870_2026_8595_MOESM1_ESM.zip › Supplementary Material/Supplement/Fig_S14_Add_Effect_All_Shoot.jpg]

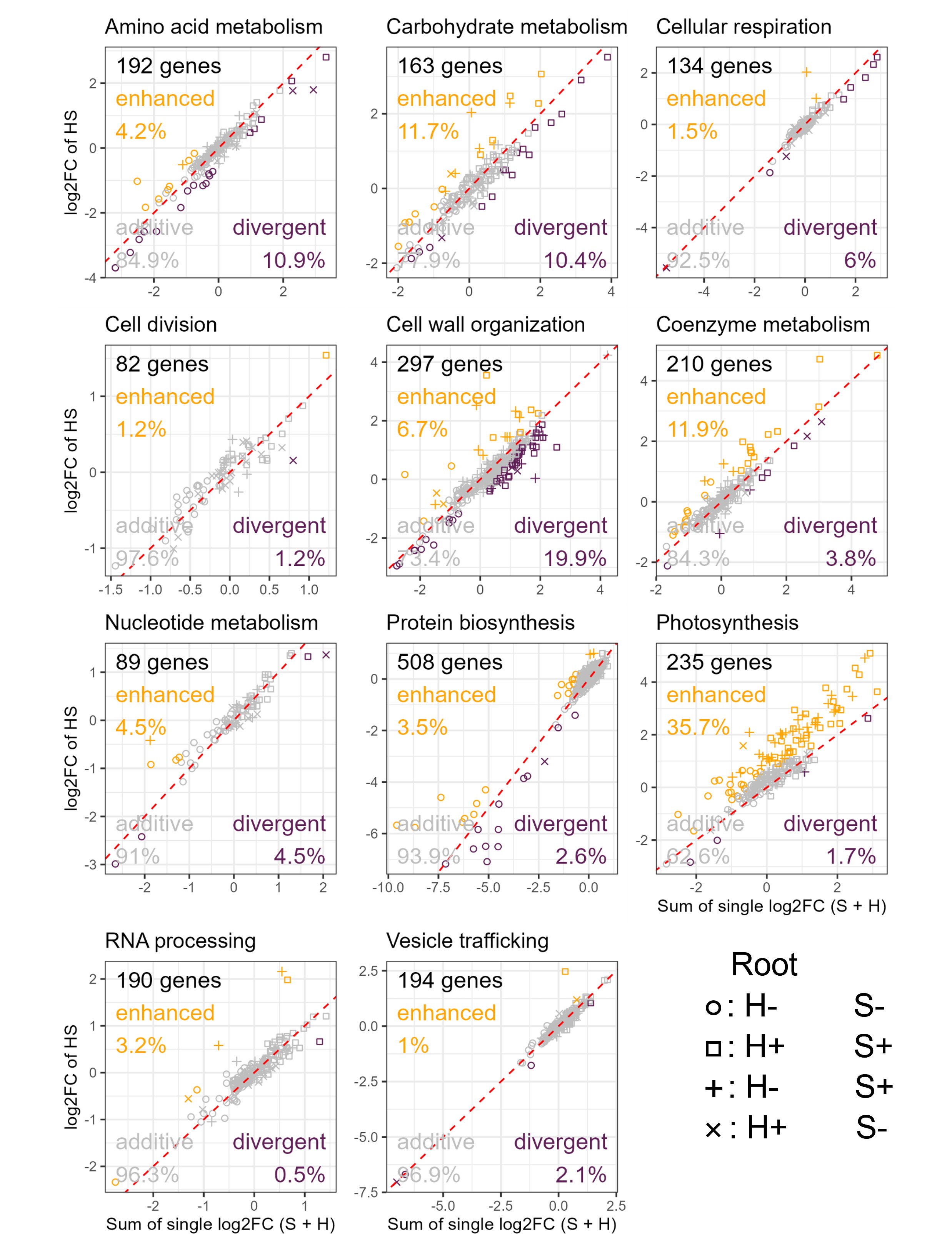

Supplement: Supplementary file 1 — Supplementary Material 1. [file 12870_2026_8595_MOESM1_ESM.zip › Supplementary Material/Supplement/Fig_S15_Add_Effect_All_Root.jpg]

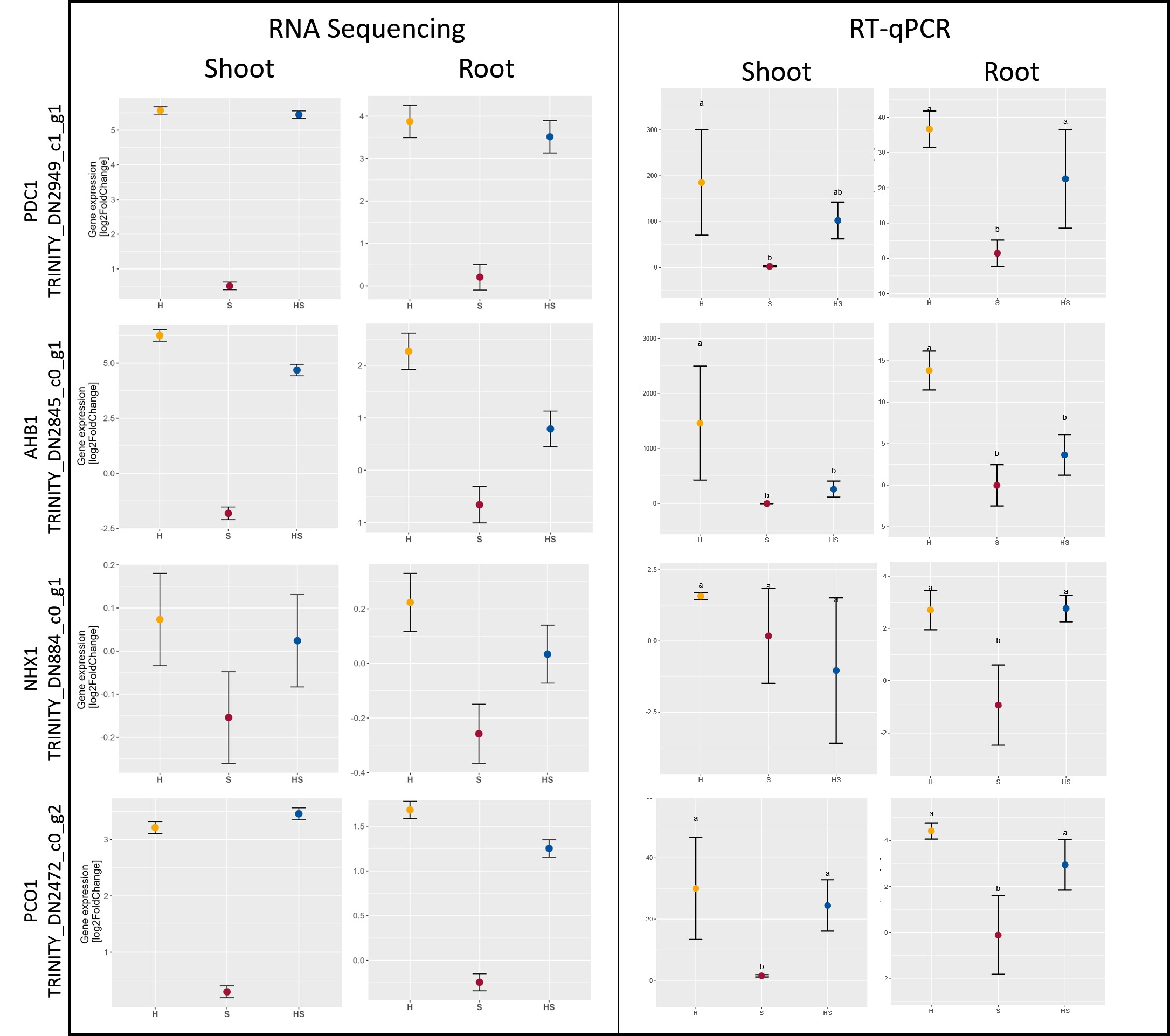

Supplement: Supplementary file 1 — Supplementary Material 1. [file 12870_2026_8595_MOESM1_ESM.zip › Supplementary Material/Supplement/Fig_S16_HRG_SRG_qPCR_1.jpg]

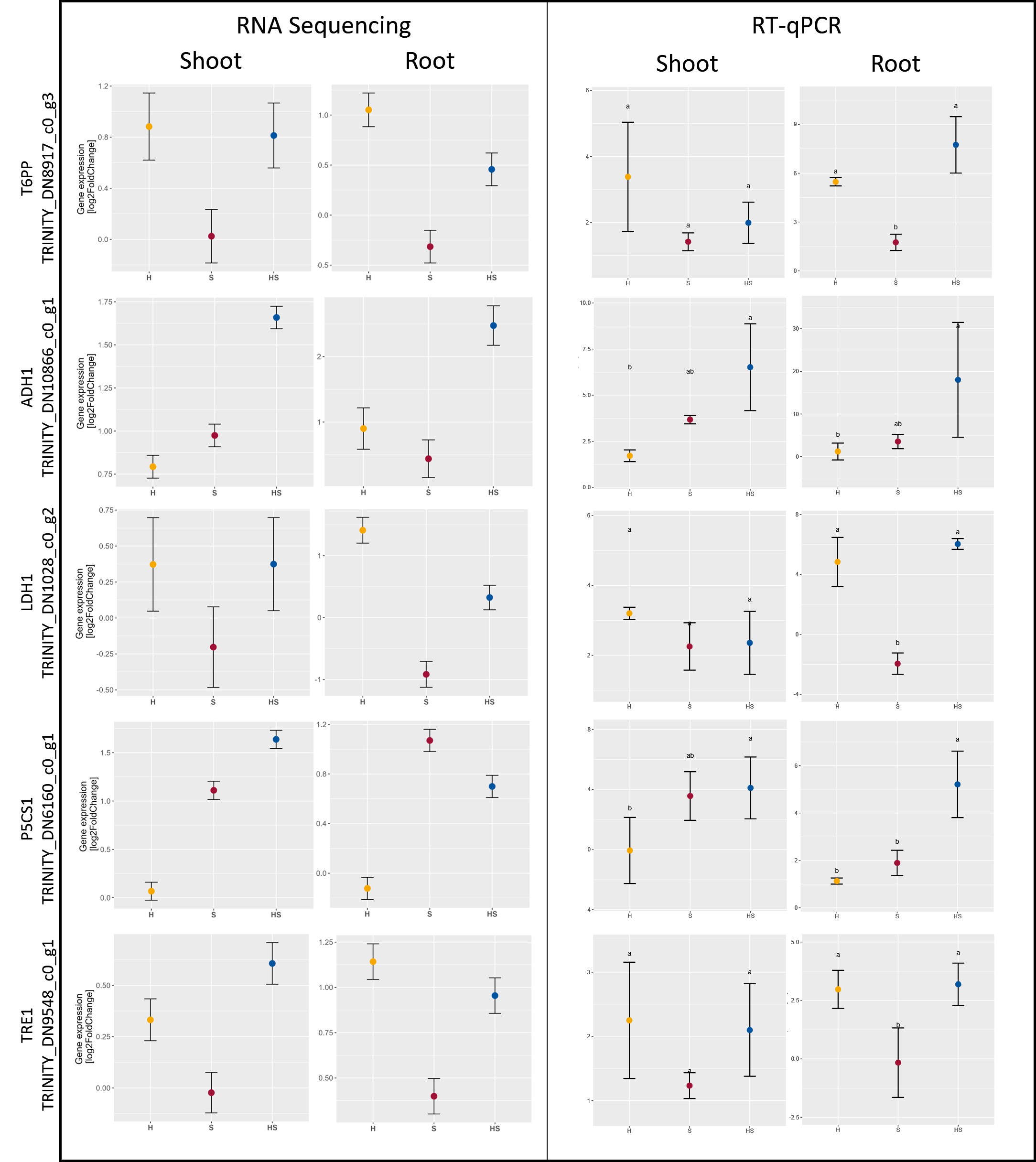

Supplement: Supplementary file 1 — Supplementary Material 1. [file 12870_2026_8595_MOESM1_ESM.zip › Supplementary Material/Supplement/Fig_S17_HRG_SRG_qPCR_2.jpg]

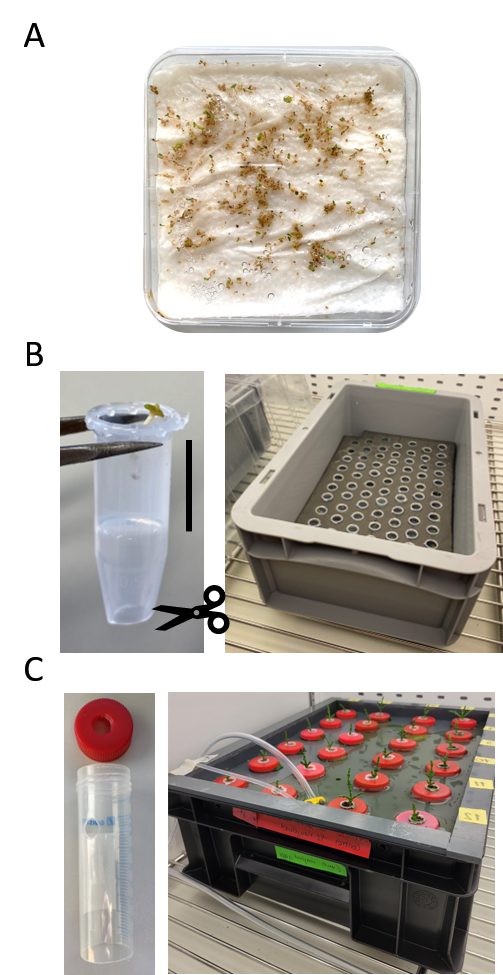

Supplement: Supplementary file 1 — Supplementary Material 1. [file 12870_2026_8595_MOESM1_ESM.zip › Supplementary Material/Supplement/Fig_S1_Cultivation.png]

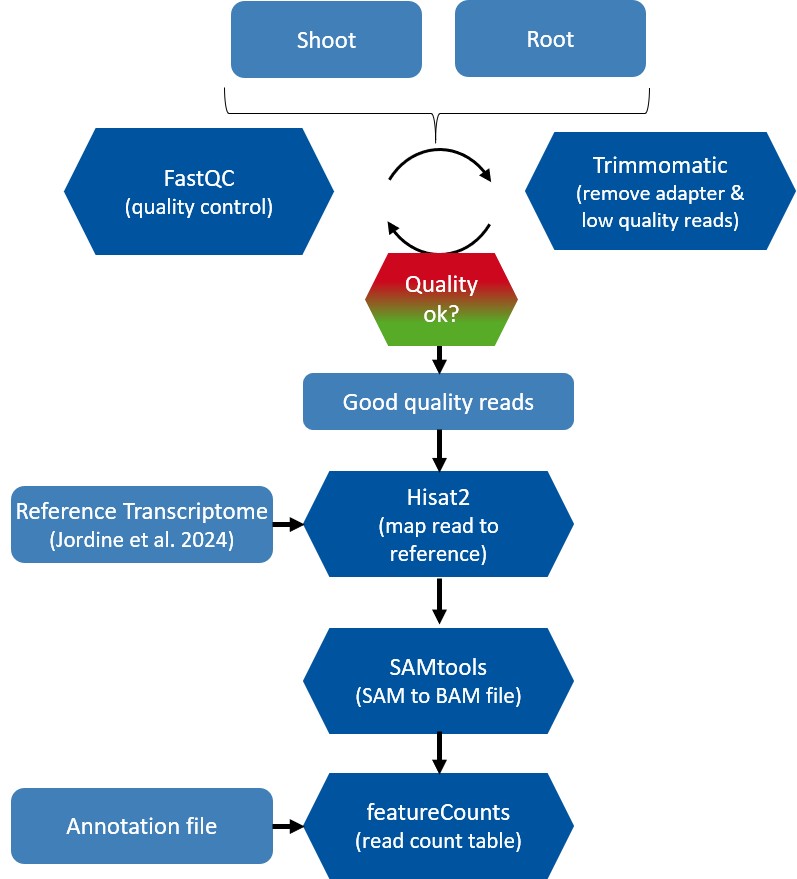

Supplement: Supplementary file 1 — Supplementary Material 1. [file 12870_2026_8595_MOESM1_ESM.zip › Supplementary Material/Supplement/Fig_S2_Anaconda.jpg]

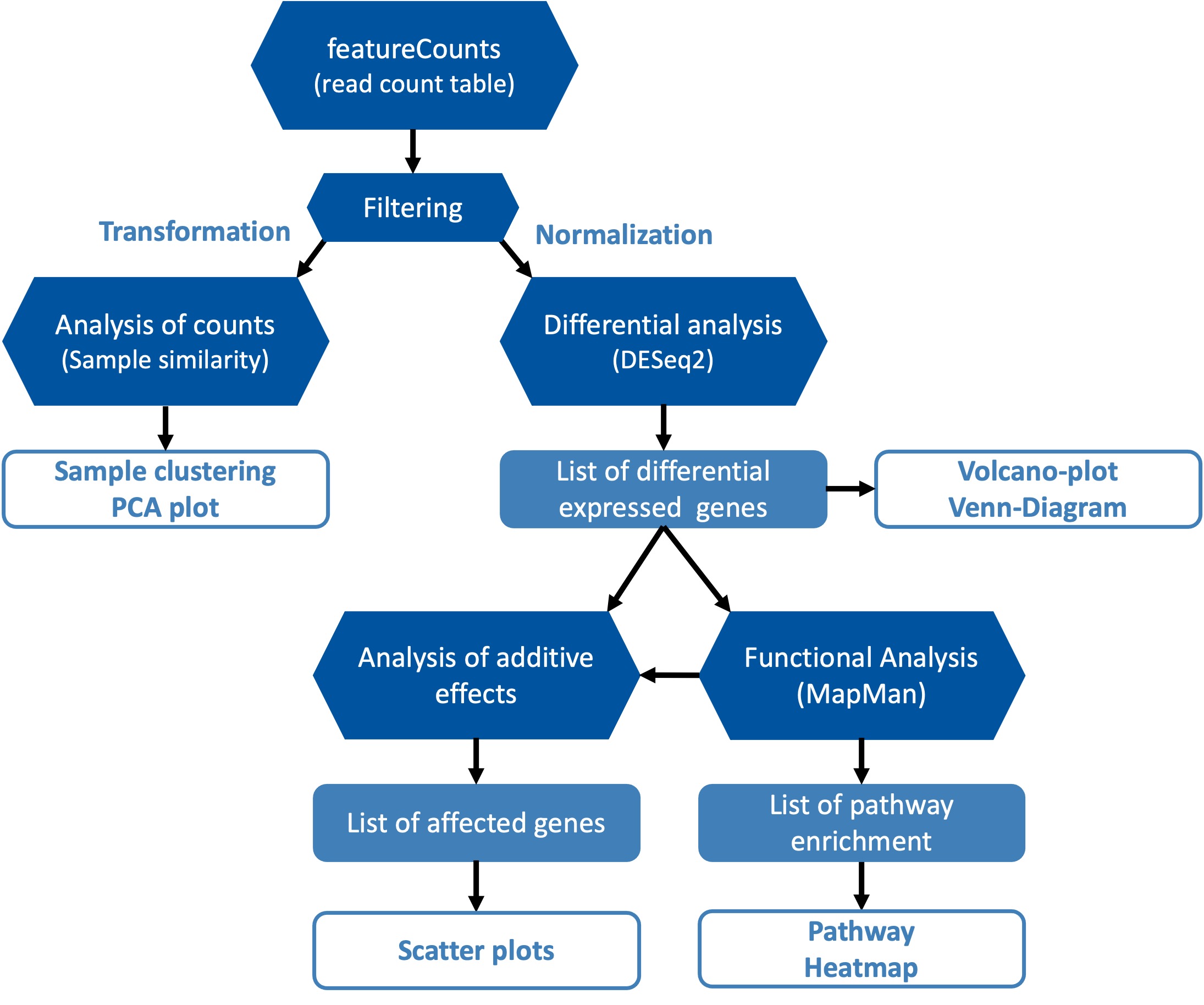

Supplement: Supplementary file 1 — Supplementary Material 1. [file 12870_2026_8595_MOESM1_ESM.zip › Supplementary Material/Supplement/Fig_S3_R_workflow.jpg]

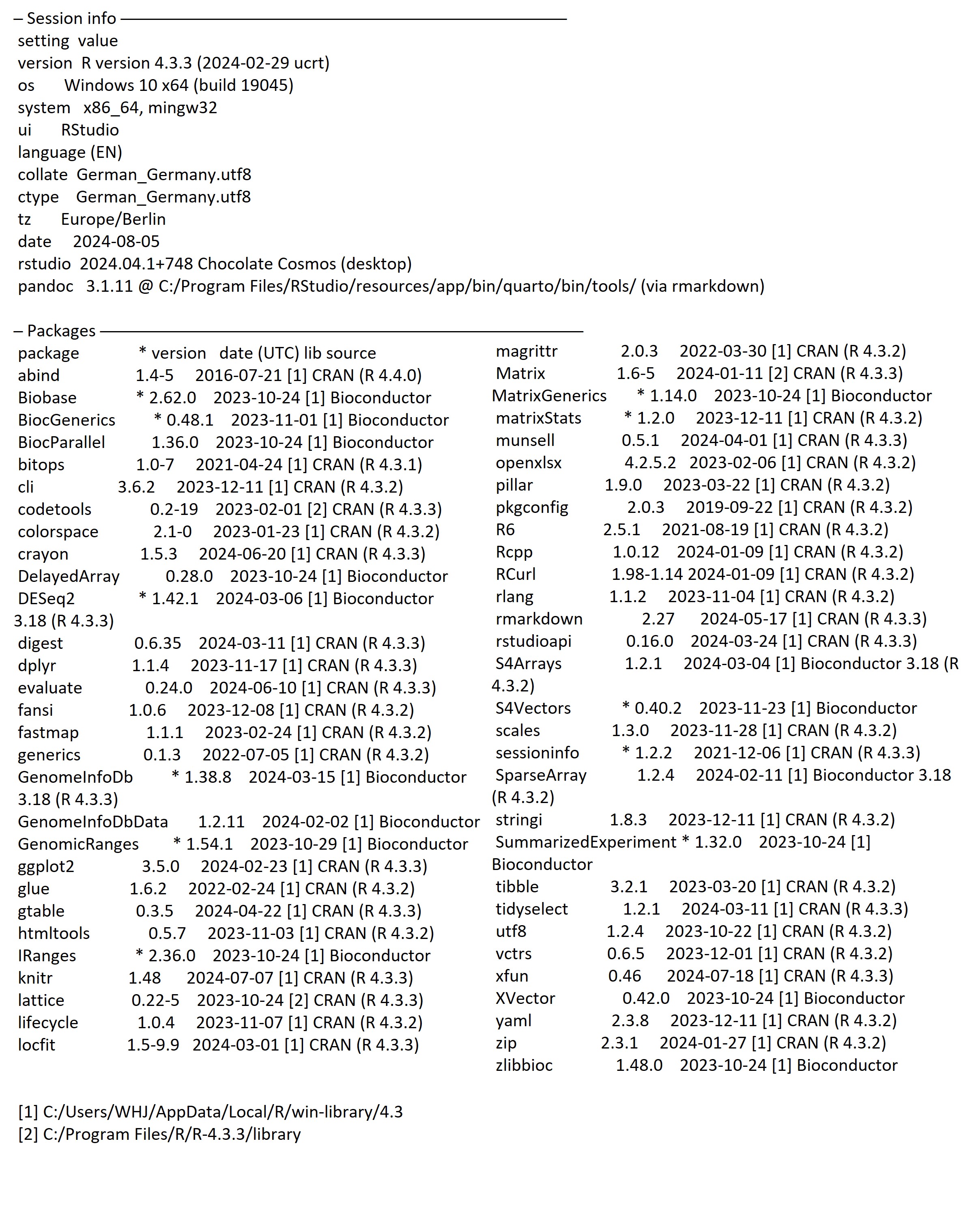

Supplement: Supplementary file 1 — Supplementary Material 1. [file 12870_2026_8595_MOESM1_ESM.zip › Supplementary Material/Supplement/Fig_S4_Session_Info_R.jpg]

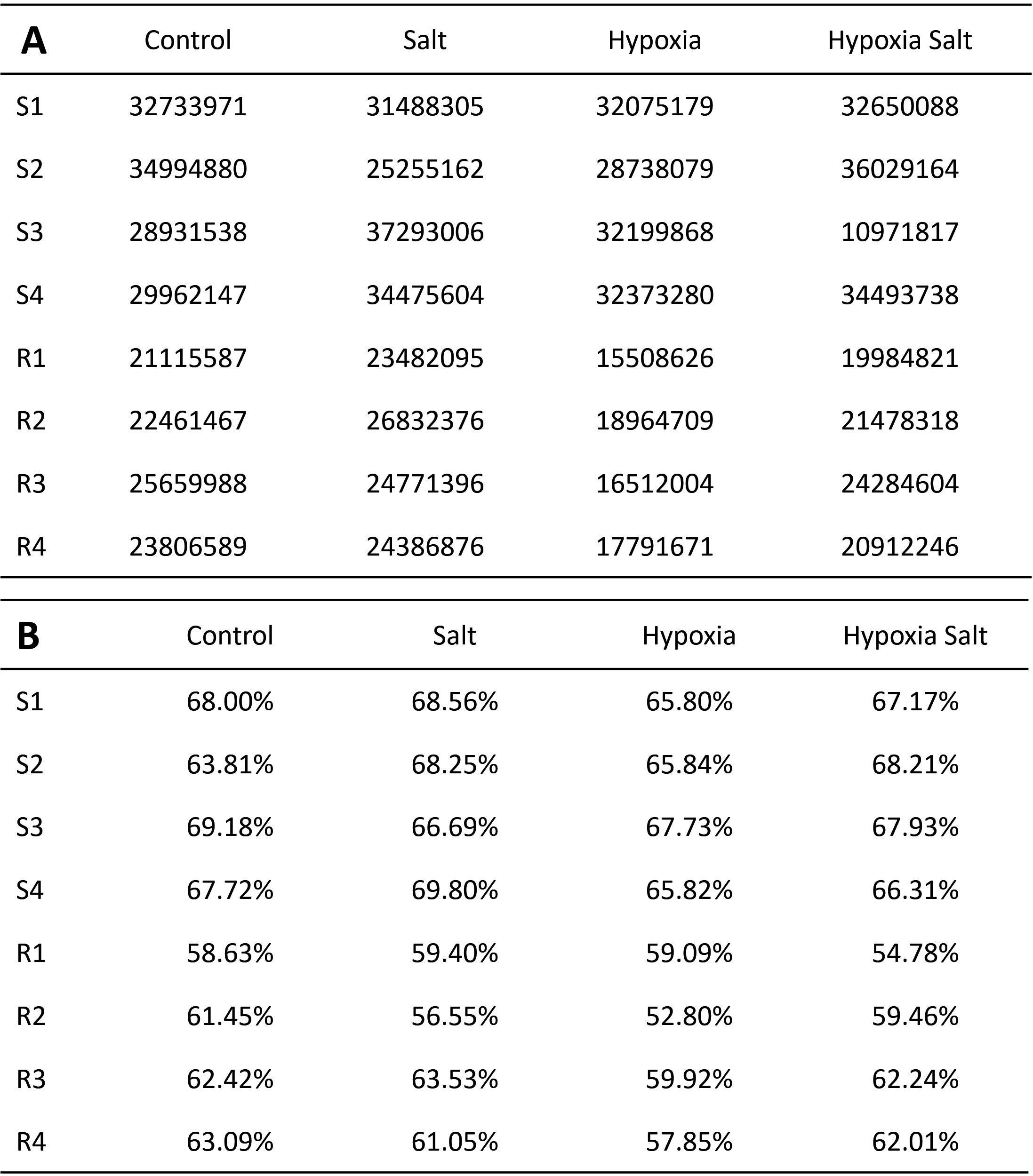

Supplement: Supplementary file 1 — Supplementary Material 1. [file 12870_2026_8595_MOESM1_ESM.zip › Supplementary Material/Supplement/Fig_S5_Alignment_data.jpg]

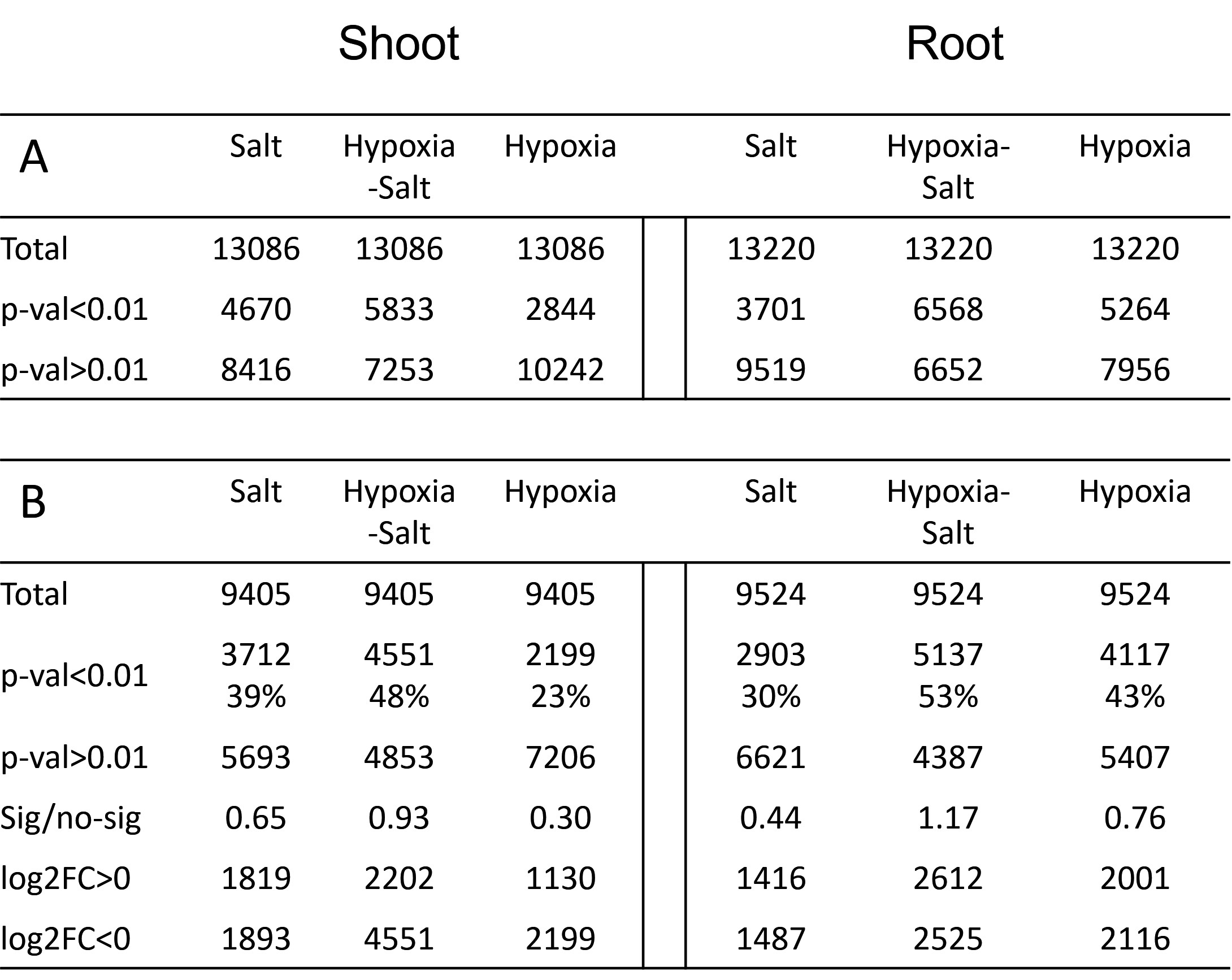

Supplement: Supplementary file 1 — Supplementary Material 1. [file 12870_2026_8595_MOESM1_ESM.zip › Supplementary Material/Supplement/Fig_S6_DEG_data.jpg]

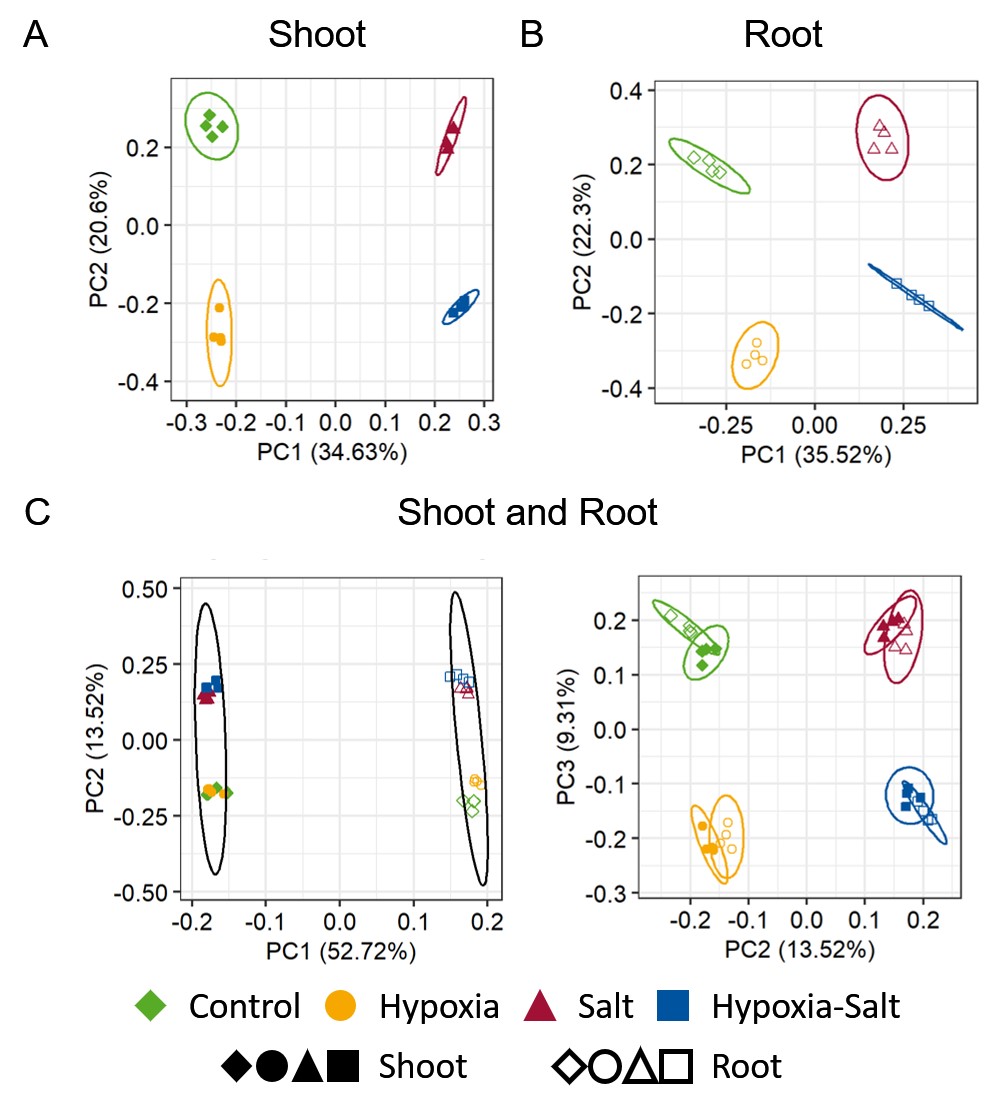

Supplement: Supplementary file 1 — Supplementary Material 1. [file 12870_2026_8595_MOESM1_ESM.zip › Supplementary Material/Supplement/Fig_S7_PCA.jpg]

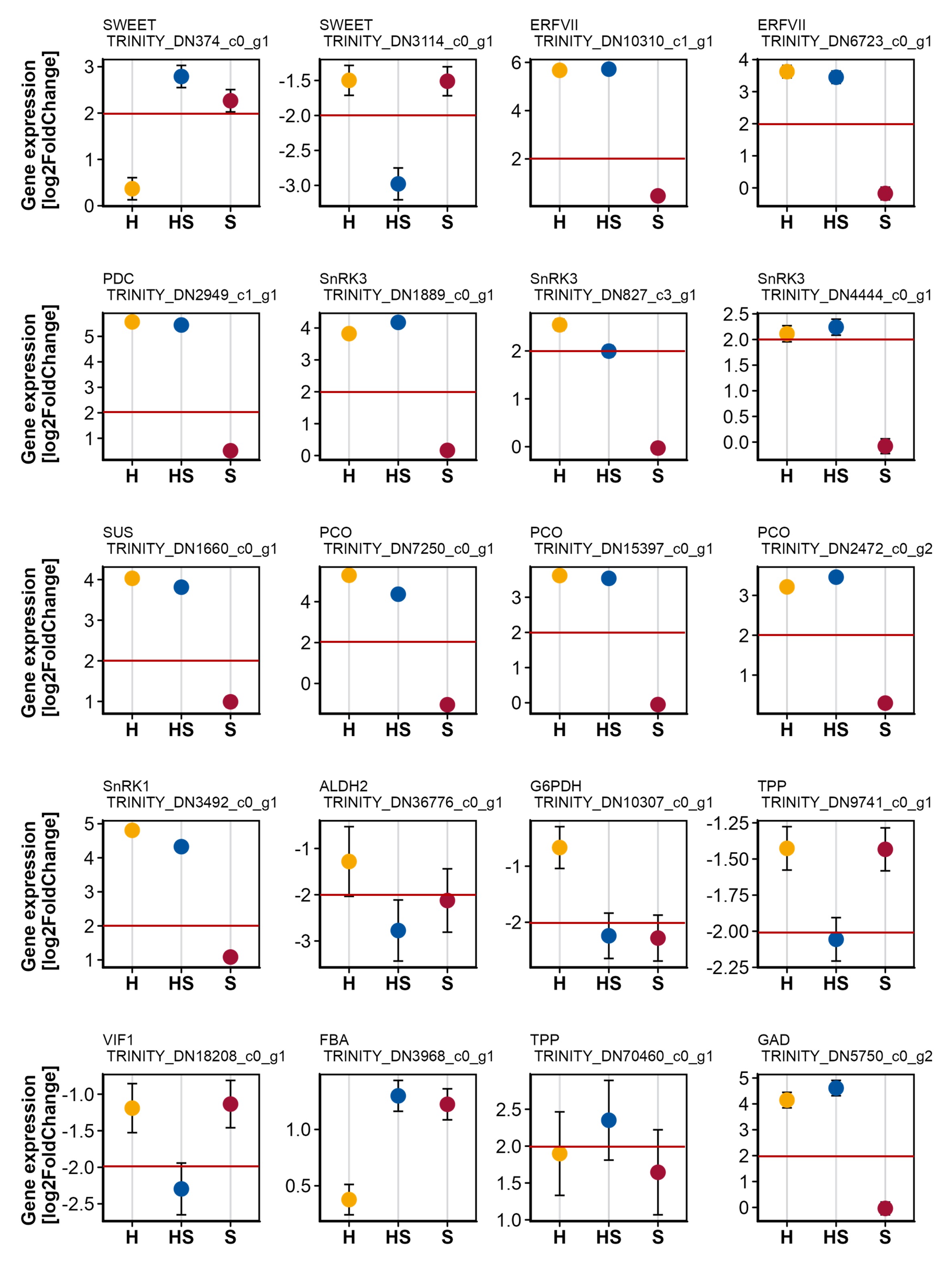

Supplement: Supplementary file 1 — Supplementary Material 1. [file 12870_2026_8595_MOESM1_ESM.zip › Supplementary Material/Supplement/Fig_S8_hVolcano_Shoot.jpg]

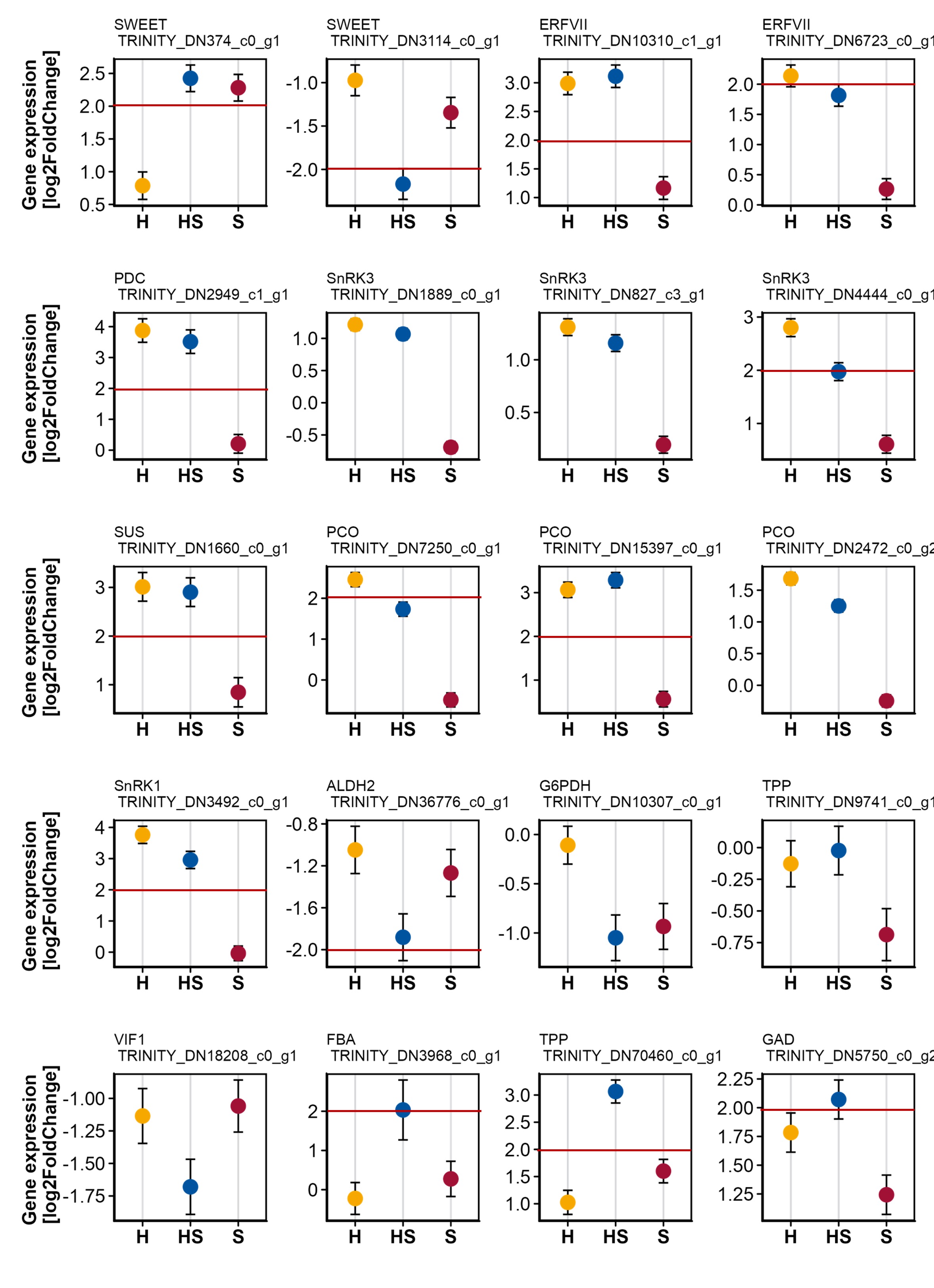

Supplement: Supplementary file 1 — Supplementary Material 1. [file 12870_2026_8595_MOESM1_ESM.zip › Supplementary Material/Supplement/Fig_S9_hVolcano_Roots.jpg]
